# Synthesis of new piperazinyl-pyrrolo[1,2-*a*]quinoxaline derivatives as inhibitors of *Candida albicans* multidrug transporters by a Buchwald–Hartwig cross-coupling reaction†

**DOI:** 10.1039/c9ra09348f

**Published:** 2020-01-15

**Authors:** Jean Guillon, Shweta Nim, Stéphane Moreau, Luisa Ronga, Solène Savrimoutou, Elisabeth Thivet, Mathieu Marchivie, Attilio Di Pietro, Rajendra Prasad, Marc Le Borgne

**Affiliations:** Univ. Bordeaux, INSERM U1212 – UMR CNRS 5320, ARNA Laboratory, UFR des Sciences Pharmaceutiques F-33076 Bordeaux Cedex France; School of Life Sciences, Jawaharlal Nehru University 110067 New Delhi India; CNRS, Univ. Bordeaux, Bordeaux INP, ICMCB, UMR 5026 F-33608 Pessac Cedex France; DRMP Group, IBCP, UMR 5086 (MMSB), CNRS/Lyon I University 69367 Lyon France; Amity Institute of Integrative Sciences and Health, Amity University Education Valley Gurgaon 122413 India; Université de Lyon, Université Claude Bernard Lyon 1, Faculté de Pharmacie – ISPB, EA 4446 Bioactive Molecules and Medicinal Chemistry, SFR Santé Lyon-Est CNRS UMS3453 – INSERM US7 Lyon France marc.le-borgne@univ-lyon1.fr

## Abstract

Two series of piperazinyl-pyrrolo[1,2-*a*]quinoxaline derivatives were prepared *via* a Buchwald–Hartwig cross-coupling reaction and then evaluated for their ability to inhibit the drug efflux activity of CaCdr1p and CaMdr1p transporters of *Candida albicans* overexpressed in a *Saccharomyces cerevisiae* strain. In the initial screening of twenty-nine piperazinyl-pyrrolo[1,2-*a*]quinoxaline derivatives, twenty-three compounds behaved as dual inhibitors of CaCdr1p and CaMdr1p. Only four compounds showed exclusive inhibition of CaCdr1p or CaMdr1p. Further biological investigations were developed and for example, their antifungal potential was evaluated by measuring the growth of control yeast cells (AD1-8u^−^) and efflux pump-overexpressing cells (AD-CDR1 and AD-MDR1) after exposition to variable concentrations of the tested compounds. The MIC_80_ values of nineteen compounds ranging from 100 to 901 μM for AD-CDR1 demonstrated that relative resistance index (RI) values were between 8 and 274. In comparison, only seven compounds had RI values superior to 4 in cells overexpressing Mdr1p. These results indicated substrate behavior for nineteen compounds for CaCdr1p and seven compounds for CaMdr1p, as these compounds were transported *via* MDR transporter overexpressing cells and not by the AD1-8u^−^ cells. Finally, in a combination assay with fluconazole, two compounds (1d and 1f) have shown a synergistic effect (fractional inhibitory concentration index (FICI) values ≤ 0.5) at micromolar concentrations in the AD-MDR1 yeast strain overexpressing CaMdr1p-protein, indicating an excellent potency toward chemosensitization.

## Abstract 2

Two series of piperazinyl-pyrrolo[1,2-*a*]quinoxaline derivatives were prepared *via* a Buchwald–Hartwig cross-coupling reaction and then evaluated for their ability to inhibit the drug efflux activity of two *Candida albicans* transporters.

## Graphical abstract

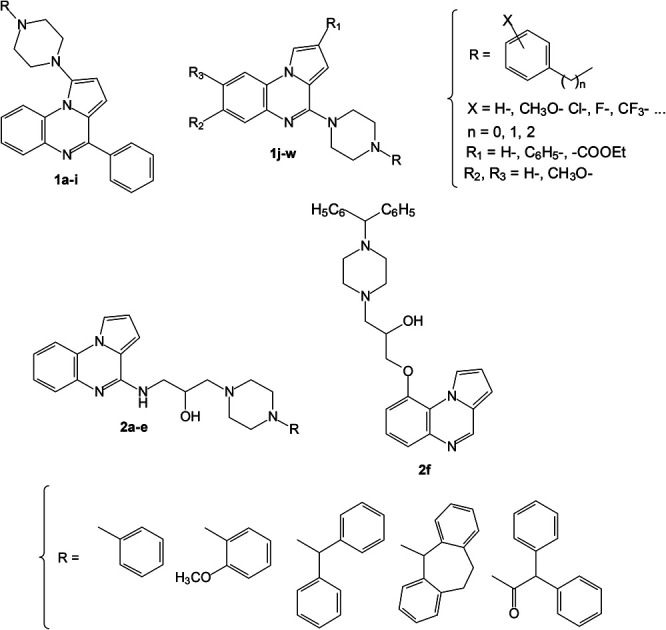

## Introduction

Transplantation surgery, cancer chemotherapy, and HIV infections have led to a worldwide rise of the immunocompromised population, and hence also of bacterial and fungal opportunistic infections.^1^ The fungal genera most often associated with invasive fungal infections include *Candida*, *Aspergillus*, and *Cryptococcus*,^2^ with opportunistic strains of *Candida albicans* accounting for approximately 50–60% causes of candidiasis, particularly in immunocompromised patients. The treatment of these *Candida* infections relies heavily on azole antifungal agents,^3^ for which widespread and prolonged use has led to the rapid emergence of multidrug resistant (MDR) isolates of *C. albicans* as well as of non-*albicans* species.^4^ Various mechanisms potentially contributing to the development of MDR have been identified, and the induction of genes encoding drug-efflux pumps, like the primary ATP binding cassette (ABC) transporter genes CaCDR1 and CaCDR2 and the secondary major-facilitator superfamily (MFS) transporter gene CaMDR1, has been shown to play a prominent role in the development of resistance to antifungal drugs.^5–7^ Overexpression of these pump proteins may lead to an increased efflux of drug substrates in MDR clinical isolates.^4,8,9^

The potent modulators of multidrug transporter CaCdr1p such as the immunosuppressants cyclosporin, FK520 and FK506, the natural polyphenol curcumin, the quorum-sensing molecule farnesol, the antabuse drug disulfiram, the antibiotic milbemycin, some synthetic-d-octapeptides, the anti-inflammatory drug ibuprofen and the antibacterial unnarmicins have been displayed to prevent drug extrusion and restore fungicidal synergism with the azoles and other drugs.^10–14^ Unlike CaCdr1p, there is only a handful number of chemosensitizers in case of CaMdr1p such as verapamil and enniantin B.^15,16^ Recently, a further screening from a library of synthetic aromatic compounds sharing a cyclobutene-dione moiety was investigated for the discovery of new inhibitors of MFS and ABC transporters of *C. albicans*. A few specific inhibitors of MFS transporter CaMdr1p were then identified.^17^ Therefore, the search for novel inhibitors able to block the drug extrusion mediated by these efflux proteins represents an attractive approach to reverse MDR.

The pyrrolo[1,2-*a*]quinoxaline heterocyclic framework constitutes the basis of an important class of compounds possessing interesting biological activities. These compounds have been reported as key intermediates for the assembly of several heterocycles including antipsychotic agent,^18^ anti-HIV agent,^19^ adenosine A_3_ receptor modulator,^20^ antiparasitic agents,^21–25^ and antitumor agents.^26–31^ We also previously demonstrated that the pyrrolo[1,2-*a*]quinoxaline heterocyclic scaffold could lead to the preparation of bacterial multidrug resistance pump inhibitors.^32,33^

**Fig. 1:**
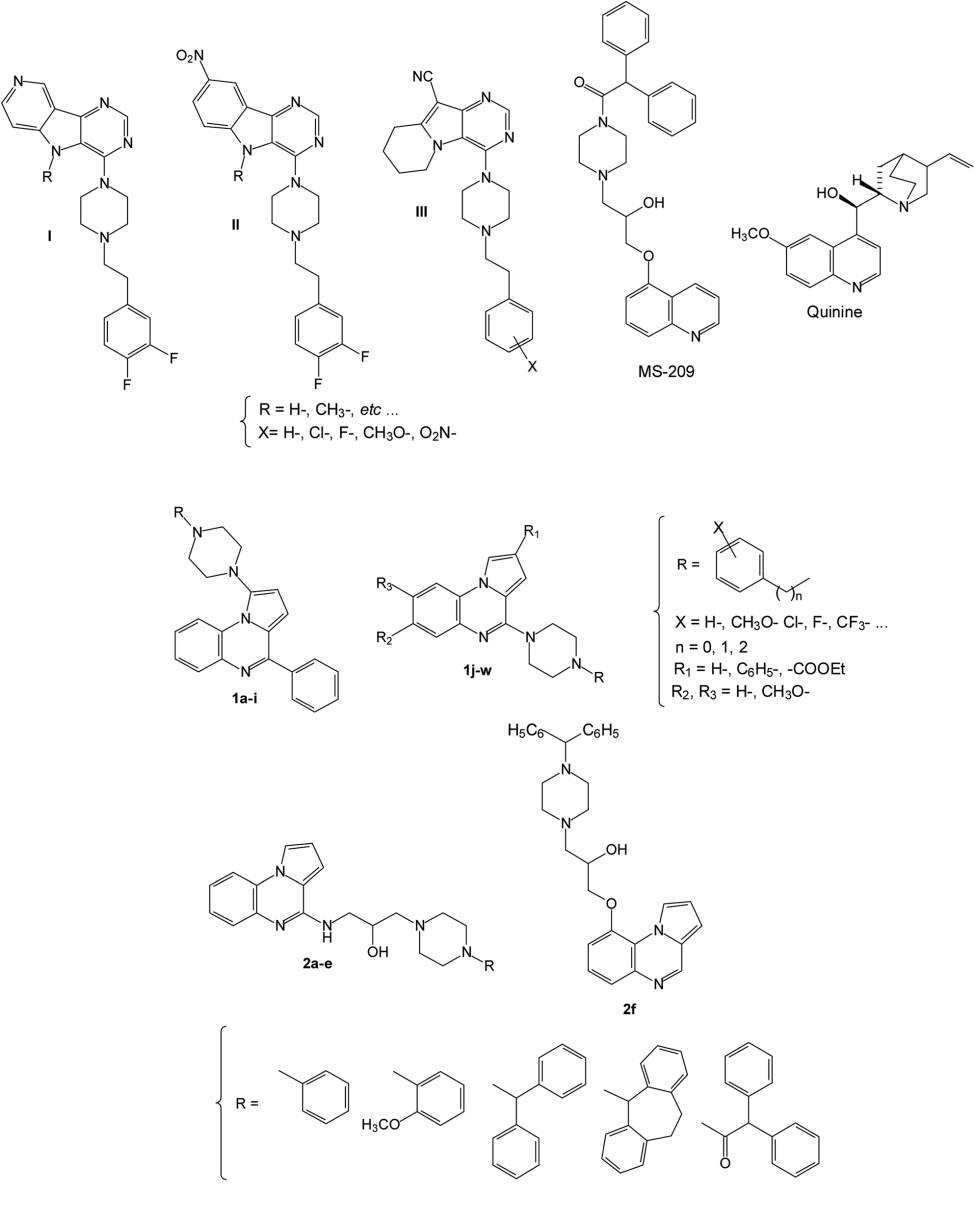
Structures of previously described EPIs I–III, quinine and MS-209, and new synthesized substituted pyrrolo[1,2-*a*]quinoxaline derivatives 1–2.

In this context and as part of a programme on the development of new efflux pump inhibitors (EPIs), we decided to broaden the structural diversity and used the pyrrolo[1,2-*a*]quinoxaline moiety as a template for the design of new derivatives 1 and 2 in which a piperazine is incorporated in position 1, 4 or 9 of the heterocyclic core in analogy with the EPI pyrimidine and quinoline derivatives I–III, quinine and MS-209 used in the various multidrug resistance therapies (Fig. 1).^34–38^

## Results and discussion

### Chemistry

**Scheme 1:**
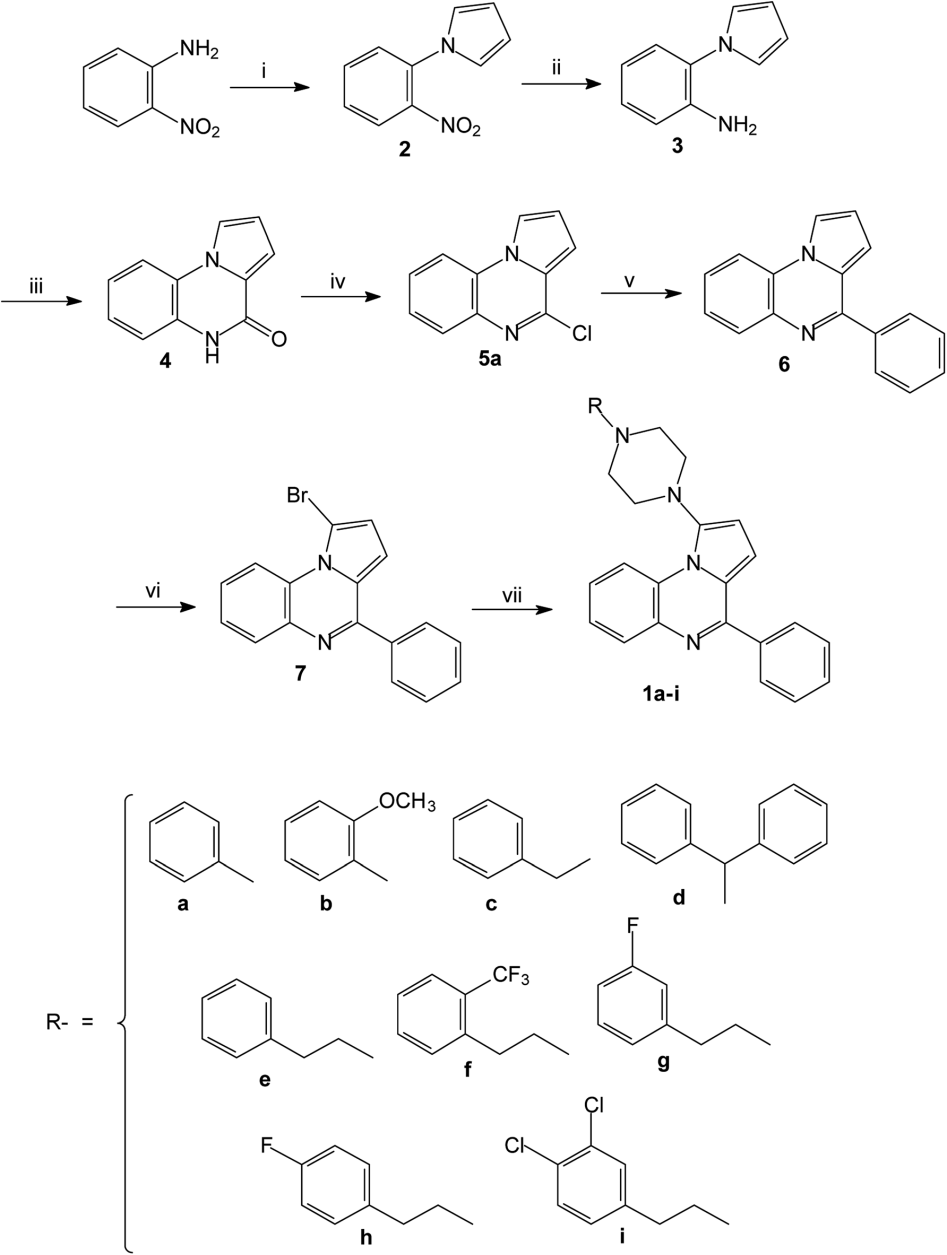
Synthesis of 1-(4-substituted-piperazinyl)-4-phenylpyrrolo[1,2-*a*]quinoxalines 1a–i; reagents and conditions: (i) 2,5-diMeOTHF, AcOH, Δ; (ii) CuSO_4_/NaBH_4_, EtOH, RT; (iii) (Cl_3_CO)_2_CO, toluene, Δ; (iv) POCl_3_, Δ; (v) (OHC–C_6_H_4_)–BF_3_K, PdCl_2_(dppf)·CH_2_Cl_2_, Cs_2_CO_3_, THF–H_2_O, Δ; (vi) NBS, CH_2_Cl_2_, RT; (vii) *R*-piperazine, Pd_2_(dba)_3_, BINAP, *t*-BuONa, toluene, 100 °C.

The reported piperazinyl-pyrrolo[1,2-*a*]quinoxaline derivatives 1a–i were synthesized in five steps from 2-nitroaniline (Scheme 1). Preparation of 1-(2-nitrophenyl)pyrrole 2 was performed according to the Clauson–Kaas reaction run under micro-wave irradiation starting from 2-nitroaniline and 2,5-dimethoxytetrahydrofuran in acetic acid. This pathway partially involved synthetic methodologies already described by our group.^21–24,28^ The resulting 1-(2-nitrophenyl)pyrrole intermediate 2 was subsequently reduced into the attempted 1-(2-aminophenyl)pyrrole 3 using a sodium borohydride-copper(ii) sulfate treatment in ethanol at room temperature. This NaBH_4_–CuSO_4_ system was found to be quite powerful in reducing our aromatic nitro group with excellent yield (85%). The reaction of 3 with triphosgene in toluene gave the lactam 4, which was subsequently chlorodehydroxylated with phosphorous oxychloride, leading to the 4-chloropyrrolo[1,2-*a*]quinoxaline 5a. The 4-phenylpyrrolo[1,2-*a*]quinoxaline 6 was easily prepared by a direct Suzuki–Miyaura cross-coupling reaction of 4-chloropyrroloquinoxalines 5a with potassium phenyltrifluoroborate performed in the presence of PdCl_2_(dppf)·CH_2_Cl_2_ as a catalyst, cesium carbonate as the base, and THF–H_2_O as the solvent system.^23^ Reaction of 6 and one equivalent of *N*-bromosuccinimide (NBS) afforded the 1-bromo-4-phenylpyrrolo[1,2-*a*]quinoxaline 7 as the sole reaction product.^28^ The Buchwald–Hartwig Pd-catalyzed amination of the 1-bromo-4-phenylpyrrolo[1,2-*a*]quinoxaline 7 using Pd_2_(dba)_3_ as catalyst with BINAP as the ligand was then investigated. Under these conditions, various substituted piperazines were successfully coupled with 7 to give the desired piperazinyl-pyrrolo[1,2-*a*]quinoxaline derivatives 1a–i by using *t*-BuONa as a base and toluene as the solvent at 100 °C.^39–41^

**Fig. 2:**
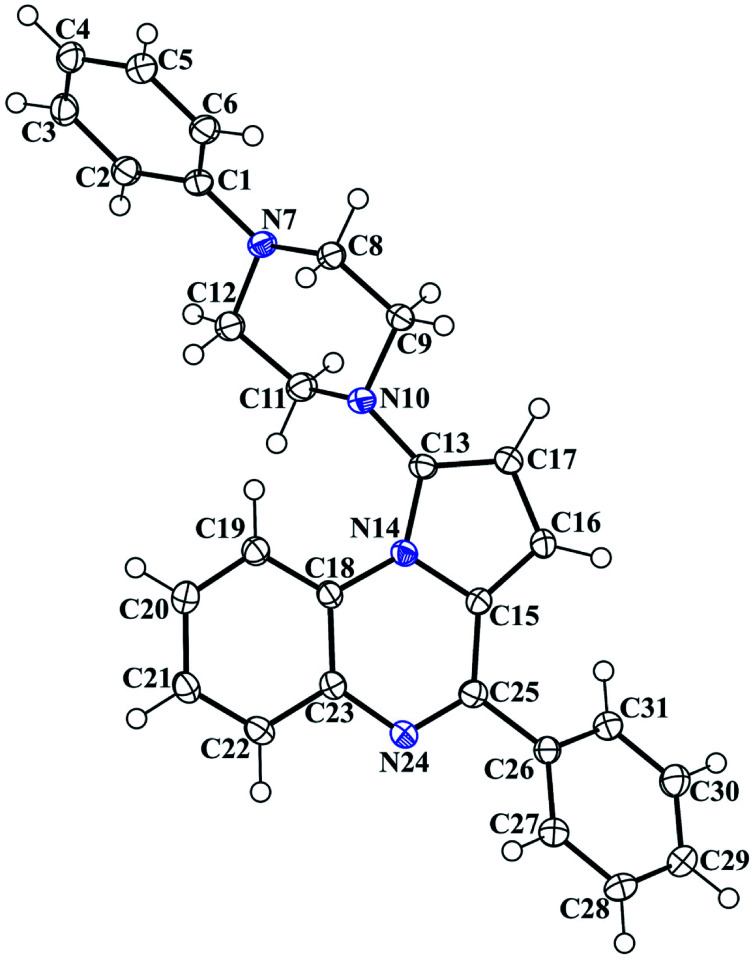
The ORTEP drawing of pyrrolo[1,2-*a*]quinoxaline 1a with thermal ellipsoids at 30% level.

**Fig. 3:**
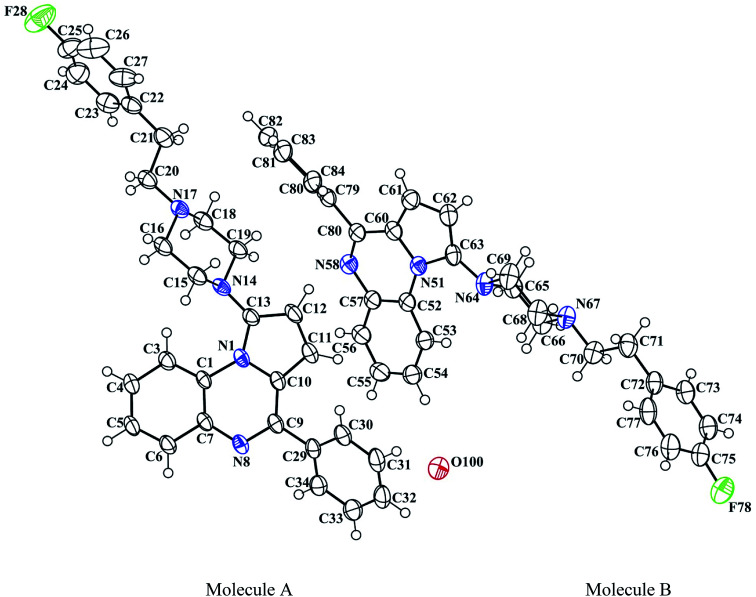
The ORTEP drawing of pyrrolo[1,2-*a*]quinoxaline 1h (molecules A and B) with thermal ellipsoids at 30% level.

The 3D structural determinations of 1a and 1h were established by X-ray crystallography (Fig. 2 and 3), and confirmed the structures in the solid state as anticipated on the basis of NMR data.

**Scheme 2:**
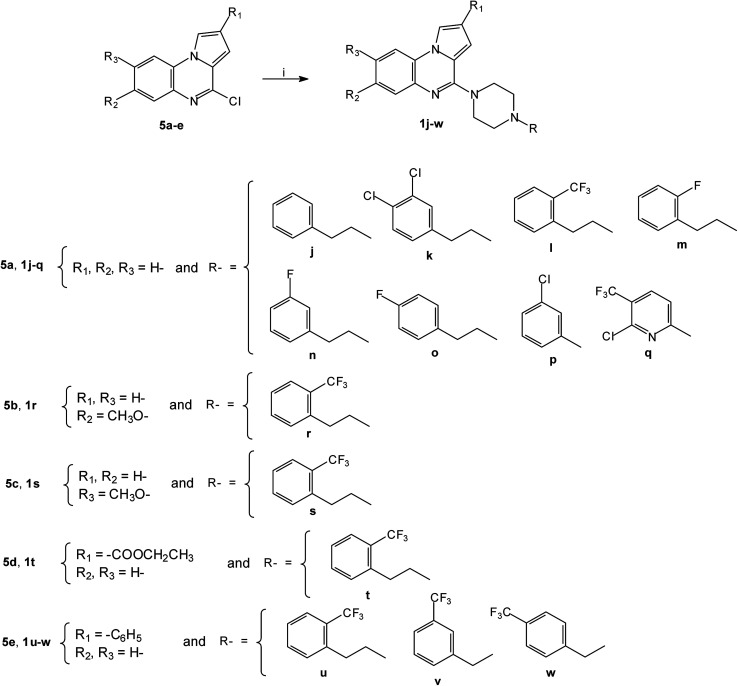
Synthesis of 4-(4-substituted-piperazinyl)-4-phenylpyrrolo[1,2-*a*]quinoxalines 1j–w; reagents and conditions: (i) *R*-piperazine, Pd_2_(dba)_3_, BINAP, *t*-BuONa, toluene, 100 °C.

By using the same cross-coupling catalyzed methodology, the Pd-catalyzed coupling of substituted piperazines with the 4-chloropyrrolo[1,2-*a*]quinoxalines 5a–e^30^ led to the new 4-(4-substituted-piperazinyl)-4-phenylpyrrolo[1,2-*a*]quinoxaline 1j–w (Scheme 2). These compounds 1a–w were then converted into their hydrochloride or oxalate salts (Table 1).

**Table 1:** Physical properties of the final amines 1a–w

Compound	a ^,^ b ^,^ cSalt	dmp (°C)	eYield (%)
1a	3HCl	114	71
1b	3HCl	88	68
1c	(COOH)_2_, H_2_O	172	58
1d	(COOH)_2_, H_2_O	109	61
1e	(COOH)_2_, H_2_O	175	52
1f	(COOH)_2_, H_2_O	156	59
1g	(COOH)_2_, H_2_O	241	53
1h	(COOH)_2_, H_2_O	238	64
1i	(COOH)_2_, H_2_O	93	59
1j	2(COOH)_2_	203	66
1k	2(COOH)_2_	144	61
1l	2(COOH)_2_	189	72
1m	2(COOH)_2_	236	74
1n	2(COOH)_2_	228	67
1n	2(COOH)_2_	215	82
1o	2(COOH)_2_	194	59
1p	2(COOH)_2_	190	63
1q	2(COOH)_2_	189	67
1r	2(COOH)_2_	212	64
1s	2(COOH)_2_	225	71
1t	2(COOH)_2_	190	60
1u	2(COOH)_2_	180	75
1v	2(COOH)_2_	251	62
1w	2(COOH)_2_	253	60

a The amines 1a, b were dissolved in 20 mL of anhydrous diethyl ether, and treated with HCl gas. After crystallization, the ammonium chlorides were collected by filtration and were washed with Et_2_O, then dried under reduced pressure.

b The amines 1c–w were dissolved in 30 mL of 2-propanol, heated to boiling, and treated with oxalic acid (4 or 5 equiv., based on the amount of the starting material). The oxalate salts crystallized upon cooling were collected by filtration, and were washed with 2-propanol and Et_2_O.

c The stoichiometry and composition of the salts were determined by elemental analyses (within ± 0.4% of the theoretical values).

d Crystallization solvent: 2-PrOH–H_2_O.

e The yields only included the conversions into the ammonium chlorides or oxalates.

**Scheme 3:**
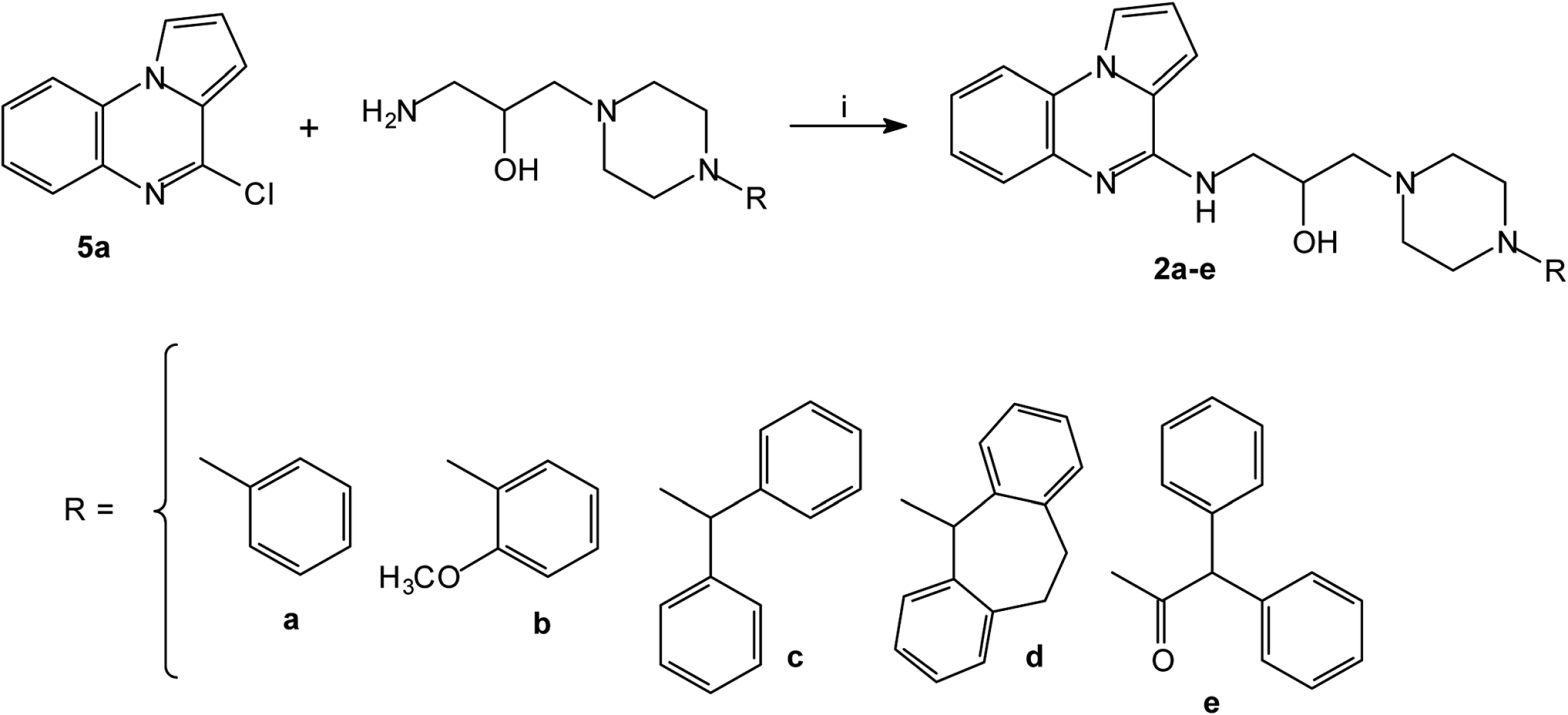
Synthesis of piperazinylalcohol-pyrrolo[1,2-*a*]quinoxalines 2a–e; reagents and conditions: (i) K_2_CO_3_, DMF, 120 °C.

**Scheme 4:**
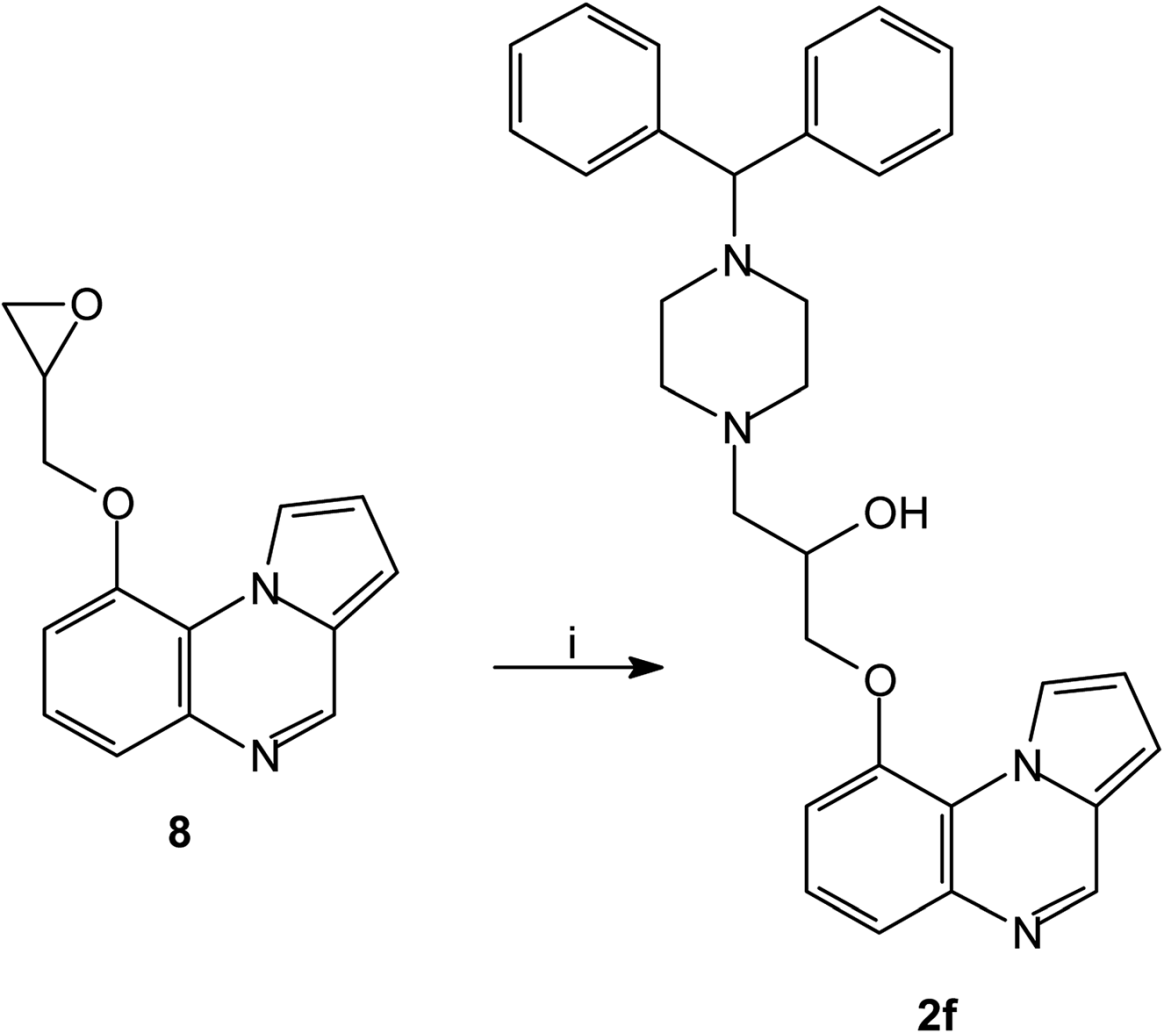
Synthesis of piperazinylalcohol-pyrrolo[1,2-*a*]quinoxaline 2f; reagents and conditions: (i) (C_6_H_5_)_2_CH-piperazine, isopropanol, Δ.

The series of piperazinylalcohol pyrrolo[1,2-*a*]quinoxaline derivatives 2a–f was synthesized as previously described by the authors starting from 5a or 8 (Schemes 3 and 4).^42^

### Biological assays

**Fig. 4:**
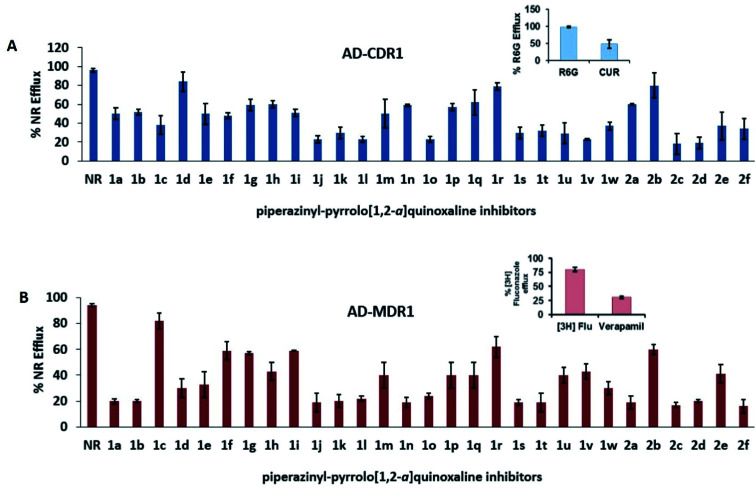
Effects of piperazinyl-pyrrolo[1,2-*a*]quinoxaline derivatives 1a–w and 2a–f on Nile Red (NR) efflux in *S. cerevisiae* cells overexpressing (A) the CaCdr1p ABC-transporter (AD-CDR1) and (B) the CaMdr1p MFS-transporter (AD-MDR1). Inhibition of rhodamine 6G (R6G) efflux in the presence of curcumin (70 μM) (CUR) was taken as positive control for CaCdr1p. Inhibition of [^3^H] fluconazole ([^3^H] Flu) efflux in the presence of verapamil (90 μM) was taken as a positive control for CaMdr1p. Values are the means ± standard deviations (error bars) for three independent experiments. Nile Red was used as the transport substrate at 7 μM, and its efflux was measured by fluorescence. Each inhibitor was used individually at a 10-fold excess over substrate (70 μM). The first column of each graph shows the Nile Red efflux in absence of any inhibitor.

Piperazinyl-pyrrolo[1,2-*a*]quinoxaline derivatives were evaluated for their ability to inhibit the drug-efflux activity of CaCdr1p and CaMdr1p transporters of *C. albicans* overexpressed in a *Saccharomyces cerevisiae* strain. The transport assay was performed by monitoring Nile Red (NR) efflux in cells overexpressing the referred efflux pumps. In this assay, compounds were compared to wild-type control cells, and when the Nile Red efflux was lower than 60%, the compound was indeed considered to have inhibitory activity (Fig. 4). The efflux study performed on twenty-nine compounds revealed the total of twenty-three compounds (1a, 1b, 1e, 1g, 1h, 1i, 1j, 1k, 1l, 1m, 1n, 1o, 1p, 1s, 1t, 1u, 1v, 1w, 2a, 2c, 2d, 2e, 2f) that behaved as dual inhibitors of CaCdr1p and CaMdr1p. Compounds 2c > 2d > 1j = 1l = 1o = 1v revealed the strongest inhibitory activity of Cdr1p efflux pump, ranging from 77 to 82%. Regarding *S. cerevisiae* cells overexpressing Mdr1p, the most active compounds were 2f > 2c > 1a = 1b = 1j = 1k = 1n = 1s = 1t = 2a = 2d > 1l > 1o ranging from 78 to 84% efflux inhibition. This initial screening identified compounds 1u, 1w and 2e as dual inhibitors with intermediate potency. Compounds 1e, 1g, 1h, 1i, 1m, 1p finally revealed a weak inhibitory activity in both cell lines overexpressing the two types of efflux pumps. Compound 1c and 1f showed exclusive but weak inhibition of CaCdr1p, whereas compounds 1d and 1q demonstrated their exclusive impact on CaMdr1p with efflux inhibition ranged from 29 to 50%. Compounds 1r and 2b are not active on both efflux pumps.

The antifungal potential of all piperazinyl-pyrrolo[1,2-*a*]quinoxaline derivatives was also evaluated by measuring the growth of control yeast cells (AD1-8u^−^) and efflux pump-overexpressing cells (AD-CDR1 and AD-MDR1) when exposed to variable concentrations of the tested compounds for 48 h. The yeast growth in the absence of inhibitor was considered as 100%. The results were expressed as MIC_80_, the concentration needed to decrease 80% of cells growth (ESI Table S1†). In case of the control yeast cells (AD1-8u^−^), compounds did not reveal any significant antifungal activity, as demonstrated by their relative resistance index (RI) values close to 1. However the MIC_80_ values of nineteen selected compounds (Table 2) ranging from 100 to 901 μM for AD-CDR1 demonstrated that RI values were comprised between 8 and 274. In comparison, only seven compounds (1c, 1e, 1g, 1j, 1k, 1m, 1o) had RI values superior to 4 in cells overexpressing Mdr1p. These results indicate the substrate behavior for both 19 compounds for CaCdr1p and seven compounds for CaMdr1p, as these compounds are transported *via* MDR transporter overexpressing cells and not by the AD1-8u^−^ cells. Interestingly, all the nineteen compounds were observed to inhibit the Nile Red transport from the AD-CDR1 cells and simultaneously behaved as substrate of CaCdr1p (Table 2). Then it could be suggested that the Nile Red and these compounds seem to share the same drug binding pocket of CDR1, undergoing the kinetics of competitive inhibition. By contrast, in the case of AD-MDR1 cells, the route of efflux transport for Nile Red and these compounds did not overlap as only seven compounds showed substrate behavior. Here the results suggest the presence of an allosteric drug binding pocket for MDR1 and thus following the path of non-competitive kinetics.

**Table 2:** Effects of compounds 1 and 2 on Nile Red (NR) efflux by CaCdr1p and CaMdr1p in *S. cerevisiae* cells either overexpressing the Cdr1p ABC-transporter (AD-CDR1) or the Mdr1p MFS-transporter (AD-MDR1)

Compounds	Yeast strains	aMIC_80_ (μM)	bRI
1c	AD1-8u^−^	99 ± 10	1
	CDR1	817 ± 87	8.25
	MDR1	400 ± 55	4.04
1e	AD1-8u^−^	12 ± 1.3	1
	CDR1	810 ± 86	67.5
	MDR1	789 ± 85	65.75
1g	AD1-8u^−^	50 ± 6	1
	CDR1	803 ± 83	16
	MDR1	221 ± 31	4.2
1j	AD1-8u^−^	3 ± 0.4	1
	CDR1	100 ± 12	33.3
	MDR1	25 ± 3	8.33
1k	AD1-8u^−^	3 ± 0.2	1
	CDR1	400 ± 49	133.3
	MDR1	12 ± 1	4
1l	AD1-8u^−^	6 ± 0.5	1
	CDR1	803 ± 102	133.8
	MDR1	12 ± 2	2
1m	AD1-8u^−^	12 ± 1.6	1
	CDR1	408 ± 52	34
	MDR1	53 ± 6.2	4.41
1n	AD1-8u^−^	25 ± 3.1	1
	CDR1	400 ± 53	16
	MDR1	50 ± 6.2	2
1o	AD1-8u^−^	6 ± 0.71	1
	CDR1	200 ± 31	33.3
	MDR1	25 ± 3.4	4.1
1r	AD1-8u^−^	6 ± 0.5	1
	CDR1	776 ± 81	129.3
	MDR1	12 ± 1.7	2
1s	AD1-8u^−^	28 ± 1.7	1
	CDR1	387 ± 47	13.8
	MDR1	59 ± 4.7	2.1
1t	AD1-8u^−^	94 ± 7.3	1
	CDR1	821 ± 75	8.7
	MDR1	102 ± 9.3	1.08
1w	AD1-8u^−^	104 ± 8.4	1
	CDR1	901 ± 77	8.6
	MDR1	187 ± 16	1.7
2a	AD1-8u^−^	47 ± 2.9	1
	CDR1	412 ± 27	8
	MDR1	91 ± 7.9	1.9
2b	AD1-8u^−^	94 ± 110	1
	CDR1	811 ± 119	8.6
	MDR1	104 ± 121	1.1
2c	AD1-8u^−^	3 ± 0.1	1
	CDR1	817 ± 77	272.3
	MDR1	6 ± 0.2	2
2d	AD1-8u^−^	6 ± 0.2	1
	CDR1	831 ± 88	138.5
	MDR1	5 ± 0.2	0.83
2e	AD1-8u^−^	3 ± 0.1	1
	CDR1	821 ± 86	273.6
	MDR1	6 ± 0.2	2
2f	AD1-8u^−^	6 ± 0.1	1
	CDR1	804 ± 71	134
	MDR1	14 ± 1.2	2.33

a The MIC_80_ values of cytotoxicity were determined by measuring the optical density of cultures of each strain in the absence and the presence of a range of concentrations of the different compounds. Yeast growth in the absence of inhibitor was considered as 100%, and the concentration where the growth was decreased to 80% was taken as MIC_80_. The values are the means ± standard deviations of three independent experiments.

b The resistance index (RI) was calculated as the ratio between the MIC_80_ value determined for the strain overexpressing the transporter relatively to that of the control strain (AD1-8u^−^).

The ability of the compounds to sensitize yeast growth to the antifungal agent fluconazole was evaluated by the checkerboard method.^43^ In this assay, the control (AD1-8u^−^) cells and the CaCdr1p- and CaMdr1p-overexpressing cells were grown in the presence of either fluconazole alone or a combination therapy (efflux pump inhibitor plus fluconazole). The results, expressed as the fractional inhibitory concentration index (FICI), are summarized in ESI Table S2.† FICI values ≤ 0.5 indicate synergistic interaction between the inhibitor and the substrate.^44^ It was observed that the two piperazinyl-pyrrolo[1,2-*a*]quinoxaline derivatives 1d and 1f with FIC = 0.0076 and 0.25, respectively, displayed strong synergistic effects (FICI = 0.15 and 0.4, respectively) when both are combined with fluconazole (FIC = 0.15) in the AD-MDR1 yeast strain overexpressing the MFS CaMdr1p, decreasing 129-fold the MIC_80_ of the antifungal agent. High FICI values (≥1) were found for the remaining compounds (ESI Table S2†). Similarly, high FICI values were also found in the AD-CDR1.

**Fig. 5:**
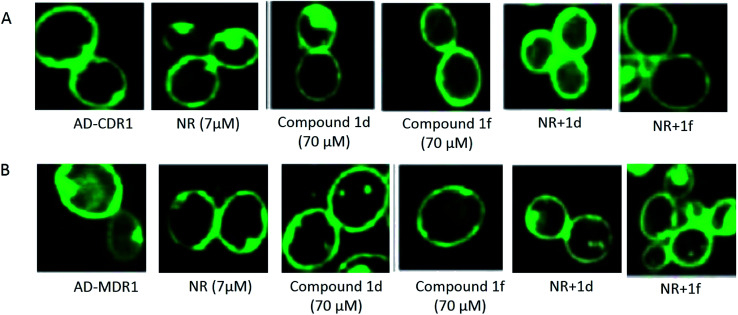
Effect of compounds 1d and 1f on the expression of the GFP-tagged Cdr1p and Mdr1p. Panels (A) AD-CDR1 and (B) AD-MDR1 were evaluated in the presence or in the absence of compounds 1d and 1f.

The effect of the compounds 1d and 1f was examined by confocal imaging of GFP-tagged Cdr1p and Mdr1p, and revealed the non-effect of these compounds on the intactness of the overexpressing strains AD-CDR1 and AD-MDR1 (Fig. 5).

Finally, compounds 1d and 1f were further evaluated for their ability to chemosensitize the azole-resistant clinical isolate (F5) of *C. albicans* together with the azole-susceptible strain (F2).^45,46^ As can be observed in Table 3, when combined with fluconazole, compounds 1d and 1f (FICI = 0.6 and 0.78, respectively) were able to reduce the effective concentration of fluconazole.

**Table 3:** Ability of compounds 1d and 1f to sensitize yeast growth to fluconazole cytotoxicity

Strain	Compound	aFIC of fluconazole	bFIC of compound	cFICI
AD1-8u^−^	1d	1 (1.5/1.5)	1 (781/781)	2 (1 + 1)
	1f	1 (1.5/1.5)	1 (811/811)	2 (1 + 1)
AD1-CDR1	1d	0.3 (81/209)	1 (791/791)	1.3 (0.3 + 1)
	1f	0.1 (40/209)	1 (791/791)	1.1 (0.1 + 1)
AD1-MDR1	1d	0.15 (10/65)	0.0076 (6.25/812)	0.15 (0.15 + 0.0076)
	1f	0.15 (10/65)	0.25 (200/799)	0.4 (0.15 + 0.25)
F2	1d	1 (13/13)	1 (618/618)	2 (1 + 1)
	1f	0.5 (7.5/13)	1 (400/400)	1.5 (0.5 + 1)
F5	1d	0.4 (200/418)	0.2 (150/720)	0.6 (0.2 + 0.4)
	1f	0.35 (150/418)	0.43 (350/799)	0.78 (0.35 + 0.43)

a Evaluated by the checkerboard method, and expressed as the fractional inhibitory concentration (FIC) values for the fluconazole (=MIC_80_ of fluconazole in combination/MIC_80_ of fluconazole alone).

b Each compound (=MIC_80_ of compound in combination/MIC_80_ of compound alone). The values in brackets are expressed in μM.

c FIC index (FICI) value ≤0.5 indicates synergistic interaction between the compound and the fluconazole.

About the structure–activity relationships on both series of piperazinyl-pyrrolo[1,2-*a*]quinoxaline derivatives, the three best dual inhibitors belonging to the series 2 (2c, 2d, 2f) has a common structural feature, namely the presence of a spacer (oxy-propan-2-ol or amino-propan-2-ol) between the tricyclic scaffold and the piperazinyl moiety. On the other hand, exclusive inhibitors of CaCdr1p (1c, 1f) or CaMdr1p (1d, 1q) have the piperazinyl moiety directly linked to the pyrroloquinoxaline. Four of these compounds (2c, 2f, 1d and 2d) have a benzhydryl substituent or related on the piperazine ring. Further pharmacomodulation works will be carried out to extend and deepen our knowledge.

## Conclusions

The chemical approach by Buchwald–Hartwig Pd-catalyzed amination of the 1-bromo-4-phenylpyrrolo[1,2-*a*]quinoxaline 7 or the 4-chloropyrrolo[1,2-*a*]quinoxalines 5a–e was successfully used to access to new piperazinyl-pyrrolo[1,2-*a*]quinoxaline derivatives 1a–w. In parallel, six other derivatives containing as well a quinoxaline moiety (compounds 2a–f, previously synthetized^42^) were added to this study. Then twenty-nine compounds have been selected on their potential to inhibit fungal multidrug resistance pumps as pyrrolo[1,2-*a*]quinoxaline template was already used and efficient for the inhibition of bacterial efflux pumps.^32,33^

Currently, we broadened our horizon to look into the role of these compounds to inhibit the CaCdr1p and CaMdr1p transporters in pathogenic yeast *C. albicans*. Our study based on the biological assays corroborated with the piperazinyl-pyrrolo[1,2-*a*]quinoxaline derivatives to be the putative and promising modulators of efflux pumps in the pathogenic yeast *C. albicans*. The results of this work have demonstrated that most of the compounds could inhibit the efflux of Nile Red mediated by both the ABC transporter CaCdr1p and the MFS pump CaMdr1p. Some compounds were able to inhibit specifically the efflux of Nile Red without being themselves substrates of the efflux-pump proteins CaMdr1p and CaMdr1p. This assumption was corroborated by the relative resistance index values close to 1 obtained from the cytotoxicity assays, showing that the presence of efflux-pump proteins did not affect the growth and the viability of yeast cells.

In *S. cerevisiae* cells expressing CaCdr1p and CaMdr1p, the greater inhibitory effect on Nile Red efflux was obtained with compounds 2c–f (5 < MIC_80_ (μM) < 14) on CaMdr1p. For compounds 2c–e, the best three compounds (MIC_80_ = 5–6 μM), the main structural feature is the presence of a bulky group *R* (*e.g.* compound 2c with a diphenylmethyl moiety).

In the combination assay with fluconazole, the two compounds 1d and 1f have shown a synergistic effect (FICI values ≤ 0.5) at micromolar concentrations in the AD-MDR1 yeast strain overexpressing CaMdr1p-protein, indicating an excellent potency toward chemosensitization. Interestingly, compound 1d showed exclusive and maximum Nile Red efflux inhibition on AD-MDR1 strain and showed excellent chemosensitization in the presence of fluconazole, whereas this was not observed with compound 1f. In this context, it is important to mention that each drug/compound may interact differently with different amino acid residues within the binding pocket of CaMdr1p, which could explain the different behavior of Nile Red and fluconazole with these compounds. As no synergy has been found in the clinical isolate F5 overexpressing CaMdr1p, a significant decreasing of the effective concentration of the antifungal agent was also observed, corroborating the results obtained in the AD-MDR1 strain. It is also interesting to note that compound 1d is the unique active compound bearing a benzhydryl moiety in the sub-series 1.

Finally, this study has shown that piperazinyl-pyrrolo[1,2-*a*]quinoxaline derivatives are able to reverse antifungal resistance, mediated by efflux pumps belonging to both ABC and MFS superfamilies of transporters of the pathogenic yeast *C. albicans*. Therefore, at non-inhibitory concentrations, these compounds stand as wise candidates chosen to be potential modulators in MDR reversal. For example, compound 1d could offer a new treatment strategy known as combo-therapy in the use of new azole antifungals recently designed.^47,48^ Nevertheless further chemical modifications will be carried out to synthetize a second generation of piperazinyl-pyrrolo[1,2-*a*]quinoxaline derivatives designed specifically as EPIs of the pathogenic yeast *C. albicans*. Once again, compound 1d will be investigated for assessing the structural importance of its benzhydryl moiety (*e.g.* nature and position of additional substituents). By similarity, a pharmacomodulation study around compound 2c will be also managed, using rational drug design tools such as 3D structural characteristics of efflux pumps^49^ and recent chemical features^50^ to design new piperazinyl-pyrrolo[1,2-*a*]quinoxaline derivatives as specific EPIs of *C. albicans*.

## Experimental section

### Chemistry

#### General information

Commercially reagents were used as received without additional purification. Melting points were determined with an SM-LUX-POL Leitz hot-stage microscope and are uncorrected. NMR spectra were recorded with tetramethylsilane as an internal standard using a Bruker Avance 300 spectrometer. Splitting patterns have been designated as follows: s = singlet; bs = broad singlet; d = doublet; t = triplet; q = quartet; dd = double doublet; ddd = double double doublet; dt = double triplet; m = multiplet. For all compounds 1a–w, all NMR spectra are available in the ESI (Fig. S1–S45†). Analytical TLC were carried out on 0.25 precoated silica gel plates (POLYGRAM SIL G/UV254) and visualization of compounds after UV light irradiation. Silica gel 60 (70–230 mesh) was used for column chromatography. Elemental analyses were found within ±0.4% of the theoretical values.

#### General procedure: synthesis of 1-piperazinyl-4-phenylpyrrolo[1,2-*a*]quinoxalines 1a–w

To a solution of 2.5 mmol of 1-bromo-4-phenylpyrrolo[1,2-*a*]quinoxaline 7 or 4-chloropyrrolo[1,2-*a*]quinoxaline 5a–e in 18 mL of anhydrous toluene were added 3 mmol (1.2 equiv.) of substituted piperazine, 0.333 g (3.5 mmol, 1.4 equiv.) of *t*-BuONa, 0.046 g (0.05 mmol, 0.02 equiv.) of tris(dibenzylideneacetone)dipalladium (0) [Pd_2_(dba)_3_] and 0.062 g (0.1 mmol, 0.04 equiv.) of 2,2′-bis(diphenylphosphino)-1,1′-binaphthalene [(±)-BINAP]. The reaction mixture was then heated at 100 °C during 15 h. After cooling, the mixture was diluted with methylene chloride. The reaction mixture was then filtered on Celite and then diluted with water. The organic layer was separated and the aqueous layer was extracted with methylene chloride (20 mL). The organic layers were collected, dried over magnesium sulfate, filtered and evaporated to dryness. Column chromatography of the residue on silica gel using ethyl acetate–methanol (8/2) as eluent gave the final product 1.

##### 1-(4-Phenylpiperazinyl)-4-phenylpyrrolo[1,2-*a*]quinoxaline (1a)

Yellow crystals (48%) mp 203 °C. ^1^H NMR (CDCl_3_) *δ* 9.21 (dd, 1H, *J* = 8.10 and 2.80 Hz, H-9), 8.02 (dd, 1H, *J* = 8.10 and 2.80 Hz, H-6), 7.96–7.93 (m, 2H, H-2′ and H-6′), 7.57–7.52 (m, 3H, H-3′, H-4′ and H-5′), 7.47–7.43 (m, 2H, H-7 and H-8), 7.35 (t, 2H, *J* = 7.70 Hz, H-3′′ and H-5′′), 7.07 (d, 2H, *J* = 7.70 Hz, H-2′′ and H-6′′), 6.96 (t, 1H, *J* = 7.70 Hz, H-4′′), 6.92 (d, 1H, *J* = 4.30 Hz, H-2), 6.59 (d, 1H, *J* = 4.30 Hz, H-3), 3.78–3.74 (m, 2H, CH_2_-pip), 3.51–3.47 (m, 2H, CH_2_-pip), 3.31–3.26 (m, 2H, CH_2_-pip), 3.18–3.09 (m, 2H, CH_2_-pip). ^13^C NMR (CDCl_3_) *δ* 156.1 (C-4), 152.6 (C-1′′), 145.1 (C-3a), 139.4 (C-5a), 138.4 (C-1′), 131.2 (C-7), 130.7 (C-8, C-3′′ and C-5′′), 130.2 (C-3′ and C-5′), 130.1 (C-9a), 130.0 (C-2′ and C-6′), 127.7 (C-9), 126.5 (C-6), 123.5 (C-1), 121.8 (C-4′), 117.9 (C-4′′), 117.8 (C-2′′ and C-6′′), 109.9 (C-2), 104.7 (C-3), 53.8 (CH_2_-pip), 50.6 (CH_2_-pip). Anal. calcd for C_27_H_24_N_4_: C, 80.17; H, 5.98; N, 13.85. Found: C, 81.04; H, 6.02; N, 13.94.

##### 1-[4-(2-Methoxyphenyl)piperazinyl]-4-phenylpyrrolo[1,2-*a*]quinoxaline (1b)

Yellow crystals (21%) mp 38 °C. ^1^H NMR (CDCl_3_) *δ* 9.23 (dd, 1H, *J* = 7.85 and 2.40 Hz, H-9), 8.01 (dd, 1H, *J* = 7.85 and 2.40 Hz, H-6), 7.96–7.94 (m, 2H, H-2′ and H-6′), 7.56–7.53 (m, 3H, H-3′, H-4′ and H-5′), 7.52–7.42 (m, 2H, H-7 and H-8), 7.13–7.06 (m, 2H, H-4′′ and H-5′′), 7.05–7.01 (m, 1H, H-6′′), 6.96 (dd, 1H, *J* = 8.50 and 2.15 Hz, H-3′′), 6.92 (d, 1H, *J* = 4.30 Hz, H-2), 6.61 (d, 1H, *J* = 4.30 Hz, H-3), 3.93 (s, 3H, CH_3_O), 3.65–3.61 (m, 2H, CH_2_-pip), 3.49–3.43 (m, 2H, CH_2_-pip), 3.31–3.12 (m, 4H, CH_2_-pip). ^13^C NMR (CDCl_3_) *δ* 156.2 (C-4), 153.7 (C-2′′), 145.2 (C-1′′), 142.4 (C-3a), 139.7 (C-5a), 138.7 (C-1′), 131.0 (C-7), 130.8 (C-8), 130.2 (C-9a), 130.1 (C-3′ and C-5′), 129.9 (C-2′ and C-6′), 127.5 (C-9), 126.3 (C-6), 124.8 (C-5′′), 123.5 (C-1), 122.4 (C-4′), 119.7 (C-4′′), 117.9 (C-6′′), 112.7 (C-3′′), 109.5 (C-2), 104.7 (C-3), 56.8 (CH_3_O), 54.0 (CH_2_-pip), 52.0 (CH_2_-pip). Anal. calcd for C_28_H_26_N_4_O: C, 77.39; H, 6.03; N, 12.89. Found: C, 77.41; H, 5.94; N, 13.04.

##### 1-(4-Benzylpiperazinyl)-4-phenylpyrrolo[1,2-*a*]quinoxaline (1c)

Yellow oil (41%). ^1^H NMR (CDCl_3_) *δ* 9.15 (dd, 1H, *J* = 8.00 and 1.70 Hz, H-9), 8.00 (dd, 1H, *J* = 8.00 and 1.70 Hz, H-6), 7.96–7.92 (m, 2H, H-2′ and H-6′), 7.54–7.51 (m, 3H, H-3′, H-4′ and H-5′), 7.48–7.45 (m, 2H, H-7 and H-8), 7.43–7.41 (m, 2H, H-2′′ and H-6′′), 7.41–7.28 (m, 3H, H-3′′, H-4′′ and H-5′′), 6.88 (d, 1H, *J* = 4.30 Hz, H-2), 6.54 (d, 1H, *J* = 4.30 Hz, H-3), 3.68 (sl, 2H, CH_2_), 3.33–3.29 (m, 2H, CH_2_-pip), 3.06–2.97 (m, 4H, CH_2_-pip), 2.60–2.55 (m, 2H, CH_2_-pip). ^13^C NMR (CDCl_3_) *δ* 156.2 (C-4), 145.2 (C-3a), 139.8 (C-5a), 139.3 (C-1′′), 138.8 (C-1′), 130.9 (C-7), 130.8 (C-8), 130.6 (C-3′ and C-5′), 130.2 (C-9a), 130.1 (C-2′′ and C-6′′), 129.9 (C-3′′ and C-5′′), 129.8 (C-2′ and C-6′), 128.7 (C-9), 127.5 (C-6), 126.2 (C-4′), 123.4 (C-1), 118.0 (C-4′′), 109.3 (C-2), 104.4 (C-3), 64.6 (NCH_2_), 54.4 (CH_2_-pip), 53.7 (CH_2_-pip). Anal. calcd for C_28_H_26_N_4_: C, 80.35; H, 6.26; N, 13.39. Found: C, 80.09; H, 6.34; N, 13.57.

##### 1-[4-(Diphenylmethyl)piperazinyl]-4-phenylpyrrolo[1,2-*a*]quinoxaline (1d)

Yellow crystals (68%) mp 69 °C. ^1^H NMR (CDCl_3_) *δ* 9.12 (dd, 1H, *J* = 7.40 and 2.20 Hz, H-9), 8.00–7.92 (m, 3H, H-6, H-2′ and H-6′), 7.55–7.52 (m, 7H, H-3′, H-4′, H-5′, H-2′′ and H-6′′), 7.45–7.41 (m, 2H, H-7 and H-8), 7.37–7.31 (m, 4H, H-3′′ and H-5′′), 7.28–7.21 (m, 2H, H-4′′), 6.90 (d, 1H, *J* = 4.30 Hz, H-2), 6.55 (d, 1H, *J* = 4.30 Hz, H-3), 4.41 (s, 1H, CH), 3.09–3.00 (m, 4H, CH_2_-pip), 2.48–2.41 (m, 2H, CH_2_-pip). ^13^C NMR (CDCl_3_) *δ* 156.1 (C-4), 145.3 (C-3a), 144.0 (2 C-1′′), 139.8 (C-5a), 138.7 (C-1′), 130.9 (C-7), 130.7 (C-8), 130.2 (C-9a), 130.1 (2 C-3′′, 2 C-5′′, C-3′ and C-5′), 129.9 (C-2′′ and C-6′′), 129.2 (C-2′ and C-6′), 128.7 (C-9), 127.5 (C-6), 126.2 (C-4′), 123.4 (C-1), 118.0 (2 C-4′′), 109.4 (C-2), 104.3 (C-3), 77.9 (NCH), 54.0 (CH_2_-pip), 53.3 (CH_2_-pip). Anal. calcd for C_34_H_30_N_4_: C, 82.56; H, 6.11; N, 11.33. Found: C, 83.27; H, 5.82; N, 10.91.

##### 1-(4-Phenethylpiperazinyl)-4-phenylpyrrolo[1,2-*a*]quinoxaline (1e)

Yellow oil (56%). ^1^H NMR (CDCl_3_) *δ* 9.15 (dd, 1H, *J* = 7.90 and 2.35 Hz, H-9), 7.99 (dd, 1H, *J* = 7.90 and 2.35 Hz, H-6), 7.96–7.92 (m, 2H, H-2′ and H-6′), 7.56–7.50 (m, 3H, H-3′, H-4′ and H-5′), 7.48–7.45 (m, 2H, H-7 and H-8), 7.37–7.32 (t, 2H, *J* = 7.20 Hz, H-2′′ and H6′′), 7.33–7.25 (m, 3H, H-3′′, H-4′′ and H-5′′), 6.89 (d, 1H, *J* = 4.30 Hz, H-2), 6.55 (d, 1H, *J* = 4.30 Hz, H-3), 3.38–3.34 (m, 2H, CH_2_-pip), 3.14–3.01 (m, 4H, CH_2_-pip), 2.95–2.90 (ddd, 2H, *J* = 12.20, 8.55 and 7.40 Hz, CH_2_), 2.82–2.76 (ddd, 2H, *J* = 12.20, 8.55 and 7.40 Hz, CH_2_), 2.65–2.58 (m, 2H, CH_2_-pip). ^13^C NMR (CDCl_3_) *δ* 156.1 (C-4), 145.2 (C-3a), 141.3 (C-5a), 139.8 (C-1′′), 138.8 (C-1′), 131.0 (C-7), 130.9 (C-8), 130.2 (C-3′ and C-5′), 130.1 (C-9a), 130.0 (C-2′′ and C-6′′), 129.9 (C-3′′, C-5′′, C-2′ and C-6′), 127.6 (C-9), 127.5 (C-6), 126.2 (C-4′), 123.5 (C-1), 117.9 (C-4′′), 109.4 (C-2), 104.5 (C-3), 61.8 (NCH_2_), 54.4 (CH_2_-pip), 53.6 (CH_2_-pip), 35.0 (CH_2_). Anal. calcd for C_29_H_28_N_4_: C, 80.52; H, 6.52; N, 12.95. Found: C, 79.96; H, 6.12; N, 12.92.

##### 1-{4-[(2-Trifluoromethyl)phenethyl]piperazinyl}-4-phenylpyrrolo[1,2-*a*]quinoxaline (1f)

Yellow crystals (63%); mp 34 °C. ^1^H NMR (CDCl_3_) *δ* 9.16 (dd, 1H, *J* = 8.20 and 2.00 Hz, H-9), 8.01 (dd, 1H, *J* = 8.20 and 2.00 Hz, H-6), 7.97–7.93 (m, 2H, H-2′ and H-6′), 7.68 (d, 1H, *J* = 7.80 Hz, H-3′′), 7.57–7.48 (m, 5H, H-4′, H-3′, H-5′, H-7 and H-8), 7.46–7.41 (m, 2H, H-4′′ and H-6′′), 7.35 (t, 1H, *J* = 7.60 Hz, H-5′′), 6.90 (d, 1H, *J* = 4.25 Hz, H-2), 6.55 (d, 1H, *J* = 4.25 Hz, H-3), 3.38–3.34 (m, 2H, CH_2_), 3.13–3.00 (m, 6H, CH_2_-pip and CH_2_), 2.81–2.76 (m, 2H, CH_2_-pip), 2.68–2.61 (m, 2H, CH_2_-pip). ^13^C NMR (CDCl_3_) *δ* 156.2 (C-4), 145.1 (C-3a), 140.1 (q, *J* = 1.6 Hz, C-1′′), 139.8 (C-1′), 138.8 (C-5a), 133.2 (C-5′′), 133.1 (C-6′′), 133.0 (C-4′′), 131.0 (C-7), 130.9 (C-8), 130.3 (q, *J* = 29.5 Hz, C-2′′), 130.2 (C-9a), 130.1 (C-3′ and C-5′), 129.9 (C-2′ and C-6′), 127.5 (C-9), 127.4 (q, *J* = 5.6 Hz, C-3′′), 126.2 (C-6), 126.0 (q, *J* = 272.2 Hz, CF_3_), 123.4 (C-1), 117.9 (C-4′), 109.3 (C-2), 104.4 (C-3), 61.6 (NCH_2_), 54.3 (CH_2_-pip), 53.7 (CH_2_-pip), 31.7 (CH_2_). Anal. calcd for C_30_H_27_F_3_N_4_: C, 71.98; H, 5.44; N, 11.19. Found: C, 72.23; H, 4.98; N, 11.02.

##### 1-[4-(3-Fluorophenethyl)piperazinyl]-4-phenylpyrrolo[1,2-*a*]quinoxaline (1g)

Yellow crystals (49%) mp 56 °C. ^1^H NMR (CDCl_3_) *δ* 9.15 (dd, 1H, *J* = 7.80 and 2.10 Hz, H-9), 8.01 (dd, 1H, *J* = 7.80 and 2.10 Hz, H-6), 7.96–7.93 (m, 2H, H-2′ and H-6′), 7.55–7.52 (m, 2H, H-3′ and H-5′), 7.48–7.43 (m, 2H, H-7 and H-8), 7.31–7.21 (m, 2H, H-5′′ and H-6′′), 7.08–6.88 (m, 2H, H-2′′ and H-4′′), 6.90 (d, 1H, *J* = 4.20 Hz, H-2), 6.56 (d, 1H, *J* = 4.20 Hz, H-3), 3.39–3.35 (m, 2H, CH_2_-pip), 3.14–3.07 (m, 4H, CH_2_-pip), 2.94–2.89 (m, 2H, CH_2_), 2.83–2.80 (m, 2H, CH_2_-), 2.64–2.61 (m, 2H, CH_2_-pip). Anal. calcd for C_29_H_27_FN_4_: C, 77.31; H, 6.04; N, 12.43. Found: C, 77.46; H, 6.15; N, 12.61.

##### 1-[4-(4-Fluorophenethyl)piperazinyl]-4-phenylpyrrolo[1,2-*a*]quinoxaline (1h)

Yellow crystals (55%) mp 150 °C. ^1^H NMR (CDCl_3_) *δ* 9.15 (dd, 1H, *J* = 7.70 and 2.10 Hz, H-9), 8.00 (dd, 1H, *J* = 7.70 and 2.10 Hz, H-6), 7.96–7.93 (m, 2H, H-2′ and H-6′), 7.55–7.51 (m, 2H, H-3′ and H-5′), 7.48–7.44 (m, 2H, H-7 and H-8), 7.30–7.21 (m, 3H, H-2′′, H-6′′ and H4′), 7.05 (t, 2H, *J* = 5.55 Hz, H-3′′ and H-5′′), 6.90 (d, 1H, *J* = 4.20 Hz, H-2), 6.56 (d, 1H, *J* = 4.20 Hz, H-3), 3.39–3.35 (m, 2H, CH_2_-pip), 3.12–3.07 (m, 4H, CH_2_-pip), 2.92–2.88 (m, 2H, CH_2_), 2.81–2.76 (m, 2H, CH_2_), 2.63–2.60 (m, 2H, CH_2_-pip). ^13^C NMR (CDCl_3_) *δ* 162.9 (d, *J* = 242.6 Hz, C-4′′), 156.2 (C-4), 144.8 (C-3a), 139.7 (C-5a), 138.8 (C-1′), 136.7 (d, *J* = 3.5 Hz, C-1′′), 131.5 (d, *J* = 7.8 Hz, C-2′′ and C-6′′), 131.0 (C-7), 130.9 (C-8), 130.1 (C-9a), 130.2 (C-3′ and C-5′), 130.0 (C-2′ and C-6′), 127.5 (C-9), 126.3 (C-6), 123.5 (C-1), 117.8 (C-4′), 116.7 (d, *J* = 20.9 Hz, C-3′′ and C-5′′), 109.4 (C-2), 104.5 (C-3), 61.7 (NCH_2_), 54.3 (CH_2_-pip), 53.4 (CH_2_-pip), 34.0 (CH_2_). Anal. calcd for C_29_H_27_FN_4_: C, 77.31; H, 6.04; N, 12.43. Found: C, 77.52; H, 6.17; N, 12.38.

##### 1-[4-(3,4-Dichlorophenylethyl)piperazinyl]-4-phenylpyrrolo[1,2-*a*]quinoxaline (1i)

Yellow oil (47%). ^1^H NMR (CDCl_3_) *δ* 9.13 (dd, 1H, *J* = 8.35 and 2.30 Hz, H-9), 8.00 (dd, 1H, *J* = 8.35 and 2.30 Hz, H-6), 7.96–7.93 (m, 2H, H-2′ and H-6′), 7.56–7.51 (m, 3H, H-2′ and H-6′), 7.56–7.51 (m, 3H, H-3′, H-4′ and H-5′), 7.49–7.43 (m, 2H, H-7 and H-8), 7.42–7.37 (m, 2H, H-3′′ and H-6′′), 7.10 (dd, 1H, *J* = 8.35 and 2.30 Hz, H-2′′), 6.89 (d, 1H, *J* = 4.30 Hz, H-2), 6.54 (d, 1H, *J* = 4.30 Hz, H-3), 3.37–3.33 (m, 2H, CH_2_), 3.08–2.98 (m, 2H, CH_2_-pip and CH_2_), 2.88–2.72 (m, 2H, CH_2_-pip), 2.64–2.56 (m, 2H, CH_2_-pip). ^13^C NMR (CDCl_3_) *δ* 156.2 (C-4), 144.9 (C-3a), 141.8 (C-5a), 139.8 (C-1′′), 138.8 (C-1′), 133.7 (C-3′′), 132.1 (C-5′′), 131.7 (C-7), 131.5 (C-4′′), 131.0 (C-8), 130.9 (C-2′′), 130.1 (C-9a), 130.0 (C-3′and C-5′), 129.9 (C-2′ and C-6′), 129.6 (C-6′′), 127.5 (C-9), 126.2 (C-6), 123.5 (C-1), 117.9 (C-4′), 109.3 (C-2), 104.4 (C-3), 61.2 (NCH_2_), 54.4 (CH_2_-pip), 53.7 (CH_2_-pip), 34.1 (CH_2_). Anal. calcd for C_29_H_26_Cl_2_N_4_: C, 69.46; H, 5.22; N, 11.17. Found: C, 69.41; H, 5.47; N, 10.95.

##### 4-(4-Phenethylpiperazinyl)pyrrolo[1,2-*a*]quinoxaline (1j)

Yellow oil (81%). ^1^H NMR (CDCl_3_) *δ* 7.84 (dd, 1H, *J* = 2.70 and 1.30 Hz, H-1), 7.76 (dd, 1H, *J* = 7.80 and 1.60 Hz, H-9), 7.72 (dd, 1H, *J* = 7.80 and 1.60 Hz, H-6), 7.37–7.25 (m, 7H, H-7, H-8, H-2′, H-3′, H-4′, H-5′ and H-6′), 6.83 (dd, 1H, *J* = 4.00 and 1.30 Hz, H-3), 6.78 (dd, 1H, *J* = 4.00 and 2.70 Hz, H-2), 3.92–3.88 (m, 4H, CH_2_-pip), 2.94–2.88 (m, 2H, CH_2_), 2.78–2.70 (m, 6H, CH_2_ and CH_2_-pip). ^13^C NMR (CDCl_3_) *δ* 154.0 (C-4), 141.8 (C-1′), 137.6 (C-5a), 130.2 (C-3′ and C-5′), 129.9 (C-2′ and C-6′), 128.9 (C-7), 127.6 (C-9), 127.2 (C-3a), 126.6 (C-6), 125.4 (C-4′), 121.6 (C-9a), 115.8 (C-8), 114.7 (C-1), 113.9 (C-2), 108.3 (C-3), 62.0 (NCH_2_), 54.7 (CH_2_-pip), 49.5 (CH_2_-pip), 35.1 (CH_2_). Anal. calcd for C_23_H_24_N_4_: C, 77.49; H, 6.78; N, 15.72. Found: C, 77.61; H, 6.92; N, 15.94.

##### 4-[4-(3,4-Dichlorophenethyl)piperazinyl]pyrrolo[1,2-*a*]quinoxaline (1k)

Yellow oil (75%). ^1^H NMR (CDCl_3_) *δ* 7.82 (dd, 1H, *J* = 2.70 and 1.40 Hz, H-1), 7.74 (dd, 1H, *J* = 7.60 and 1.40 Hz, H-9), 7.70 (dd, 1H, *J* = 7.60 and 1.40 Hz, H-6), 7.38–7.25 (m, 4H, H-7, H-8, H-2′ and H-5′), 7.09 (dd, 1H, *J* = 8.10 and 1.80 Hz, H-6′), 6.80 (dd, 1H, *J* = 4.00 and 1.30 Hz, H-3), 6.77 (dd, 1H, *J* = 4.00 and 2.70 Hz, H-2), 3.87–3.83 (m, 4H, CH_2_-pip), 2.85–2.80 (m, 2H, CH_2_), 2.76–2.64 (m, 6H, CH_2_ and CH_2_-pip). ^13^C NMR (CDCl_3_) *δ* 153.9 (C-4), 142.0 (C-1′), 137.5 (C-5a), 133.5 (C-3′), 132.0 (C-5′), 131.6 (C-2′), 131.4 (C-4′), 129.6 (C-6′), 128.9 (C-7), 127.2 (C-3a), 126.6 (C-9), 125.4 (C-6), 121.5 (C-9a), 115.8 (C-8), 114.7 (C-1), 113.9 (C-2), 108.2 (C-3), 61.2 (NCH_2_), 54.5 (CH_2_-pip), 49.3 (CH_2_-pip), 34.0 (CH_2_). Anal. calcd for C_23_H_22_Cl_2_N_4_: C, 64.94; H, 5.21; N, 13.17. Found: C, 65.08; H, 5.36; N, 13.35.

##### 4-[4-(2-Trifluoromethylphenethyl)piperazinyl]pyrrolo[1,2-*a*]quinoxaline (1l)

Yellow oil (79%). ^1^H NMR (CDCl_3_) *δ* 7.84 (dd, 1H, *J* = 2.70 and 1.30 Hz, H-1), 7.75 (dd, 1H, *J* = 7.80 and 1.40 Hz, H-9), 7.71 (dd, 1H, *J* = 7.80 and 1.40 Hz, H-6), 7.66 (d, 1H, *J* = 7.60 Hz, H-3′), 7.50 (t, 1H, *J* = 7.60 Hz, H-5′), 7.42 (d, 1H, *J* = 7.60 Hz, H-6′), 7.36–7.26 (m, 3H, H-7, H-8 and H-4′), 6.81 (dd, 1H, *J* = 4.00 and 1.30 Hz, H-3), 6.78 (dd, 1H, *J* = 4.00 and 2.70 Hz, H-2), 3.91–3.87 (m, 4H, CH_2_-pip), 3.11–3.06 (m, 2H, CH_2_), 2.81–2.70 (m, 6H, CH_2_ and CH_2_-pip). ^13^C NMR (CDCl_3_) *δ* 153.9 (C-4), 140.2 (q, *J* = 1.6 Hz, C-1′), 137.6 (C-5a), 133.2 (q, *J* = 0.8 Hz, C-5′), 132.1 (C-6′), 130.0 (q, *J* = 29.4 Hz, C-2′), 128.9 (C-7), 127.6 (C-4′), 127.3 (q, *J* = 5.6 Hz, C-3′), 127.2 (C-3a), 126.5 (C-9), 126.1 (q, *J* = 272.1 Hz, CF_3_), 125.3 (C-6), 121.6 (C-9a), 115.8 (C-8), 114.7 (C-1), 113.9 (C-2), 108.2 (C-3), 61.6 (NCH_2_), 54.5 (CH_2_-pip), 49.4 (CH_2_-pip), 31.4 (CH_2_). Anal. calcd for C_24_H_23_F_3_N_4_: C, 67.91; H, 5.46; N, 13.20. Found: C, 68.05; H, 5.22; N, 13.38.

##### 4-[4-(2-Fluorophenethyl)piperazinyl]pyrrolo[1,2-*a*]quinoxaline (1m)

Yellow oil (74%). ^1^H NMR (CDCl_3_) *δ* 7.85–7.82 (m, 1H, H-1), 7.76 (dd, 1H, *J* = 7.80 and 1.40 Hz, H-9), 7.70 (dd, 1H, *J* = 7.80 and 1.40 Hz, H-6), 7.36–7.04 (m, 6H, H-7, H-8, H-3′, H-4′, H-5′ and H-6′), 6.81–6.77 (m, 2H, H-2 and H-3), 3.97–3.94 (m, 4H, CH_2_-pip), 3.01–2.98 (m, 2H, CH_2_), 2.87–2.83 (m, 6H, CH_2_ and CH_2_-pip). ^13^C NMR (CDCl_3_) *δ* 162.6 (d, *J* = 243.3 Hz, C-2′), 153.9 (C-4), 137.6 (C-5a), 132.4 (d, *J* = 5 Hz, C-6′), 129.3 (d, *J* = 8 Hz, C-4′), 128.9 (C-7), 128.5 (d, *J* = 15.8 Hz, C-1′), 127.2 (C-3a), 126.5 (C-9), 125.4 (d, *J* = 3.2 Hz, C-5′), 125.3 (C-6), 121.6 (C-9a), 116.7 (d, *J* = 22.1 Hz, C-3′), 115.8 (C-8), 114.7 (C-1), 113.8 (C-2), 108.2 (C-3), 60.2 (NCH_2_), 54.5 (CH_2_-pip), 49.4 (CH_2_-pip), 28.2 (CH_2_). Anal. calcd for C_23_H_23_FN_4_: C, 73.77; H, 6.19; N, 14.96. Found: C, 73.85; H, 6.26; N, 15.12.

##### 4-[4-(3-Fluorophenethyl)piperazinyl]pyrrolo[1,2-*a*]quinoxaline (1n)

Yellow oil (81%). ^1^H NMR (CDCl_3_) *δ* 7.83 (dd, 1H, *J* = 2.70 and 1.30 Hz, H-1), 7.73 (dd, 1H, *J* = 8.00 and 1.50 Hz, H-9), 7.70 (dd, 1H, *J* = 8.00 and 1.50 Hz, H-6), 7.38–7.23 (m, 3H, H-7, H-8 and H-5′), 7.05–6.89 (m, 3H, H-2′, H-4′ and H-6′), 6.81 (dd, 1H, *J* = 3.90 and 1.30 Hz, H-3), 6.77 (dd, 1H, *J* = 3.90 and 2.70 Hz, H-2), 3.91–3.86 (m, 4H, CH_2_-pip), 2.93–2.87 (m, 2H, CH_2_), 2.78–2.69 (m, 6H, CH_2_ and CH_2_-pip). ^13^C NMR (CDCl_3_) *δ* 164.3 (d, *J* = 244.1 Hz, C-3′), 153.7 (C-4), 143.5 (d, *J* = 7.35 Hz, C-1′), 137.3 (C-5a), 131.3 (d, *J* = 8.25 Hz, C-5′), 128.9 (C-7), 127.2 (C-3a), 126.6 (C-9), 125.8 (d, *J* = 2.80 Hz, C-6′), 125.6 (C-6), 121.4 (C-9a), 117.0 (d, *J* = 20.85 Hz, C-2′), 115.9 (C-8), 114.7 (C-1), 114.5 (d, *J* = 21.5 Hz, C-4′), 113.9 (C-2), 108.1 (C-3), 61.2 (NCH_2_), 54.3 (CH_2_-pip), 48.8 (CH_2_-pip), 34.1 (CH_2_). Anal. calcd for C_23_H_23_FN_4_: C, 73.77; H, 6.19; N, 14.96. Found: C, 73.71; H, 6.33; N, 15.16.

##### 4-[4-(4-Fluorophenethyl)piperazinyl]pyrrolo[1,2-*a*]quinoxaline (1o)

Yellow oil (85%). ^1^H NMR (CDCl_3_) *δ* 7.83 (dd, 1H, *J* = 2.70 and 1.20 Hz, H-1), 7.75 (dd, 1H, *J* = 8.00 and 1.50 Hz, H-9), 7.70 (dd, 1H, *J* = 8.00 and 1.50 Hz, H-6), 7.37–7.26 (m, 2H, H-7 and H-8), 7.21 (dd, 2H, *J* = 8.70 and 5.40 Hz, H-2′ and H-6′), 7.04–6.97 (m, 2H, H-3′ and H-5′), 6.81 (dd, 1H, *J* = 3.90 and 1.20 Hz, H-3), 6.77 (dd, 1H, *J* = 3.90 and 2.70 Hz, H-2), 3.91–3.86 (m, 4H, CH_2_-pip), 2.89–2.84 (m, 2H, CH_2_), 2.77–2.66 (m, 6H, CH_2_ and CH_2_-pip). ^13^C NMR (CDCl_3_) *δ* 162.8 (d, *J* = 242.6 Hz, C-4′), 153.8 (C-4), 137.4 (C-5a), 137.0 (d, *J* = 3.2 Hz, C-1′), 131.5 (d, *J* = 7.7 Hz, C-2′ and C-6′), 128.9 (C-7), 127.2 (C-3a), 126.6 (C-9), 125.5 (C-6), 121.5 (C-9a), 116.6 (d, *J* = 21.2 Hz, C-3′ and C-5′), 115.8 (C-8), 114.7 (C-1), 113.9 (C-2), 108.1 (C-3), 61.8 (NCH_2_), 54.5 (CH_2_-pip), 49.1 (CH_2_-pip), 33.9 (CH_2_). Anal. calcd for C_23_H_23_FN_4_: C, 73.77; H, 6.19; N, 14.96. Found: C, 73.94; H, 6.05; N, 15.09.

##### 4-[4-(3-Chlorophenyl)piperazinyl]pyrrolo[1,2-*a*]quinoxaline (1p)

Pale-yellow oil (73%). ^1^H NMR (CDCl_3_) *δ* 7.90–7.88 (m, 1H, H-1), 7.82–7.76 (m, 2H, H-9 and H-6), 7.43–7.28 (m, 2H, H-7 and H-8), 7.26 (t, 1H, *J* = 8.10 Hz, H-5′), 7.00 (dd, 1H, *J* = 1.50 and 1.50 Hz, H-2′), 6.93–6.83 (m, 4H, H-4′, H-6′, H-2 and H-3), 4.01–3.97 (m, 4H, CH_2_-pip), 3.47–3.43 (m, 4H, CH_2_-pip). ^13^C NMR (CDCl_3_) *δ* 153.9 (C-4), 153.6 (C-1′), 137.3 (C-5a), 136.4 (C-3′), 131.5 (C-5′), 128.9 (C-7), 127.2 (C-3a), 126.7 (C-9), 125.7 (C-6), 121.5 (C-9a), 120.8 (C-8), 117.1 (C-4′), 116.0 (C-2′), 115.2 (C-6′), 114.8 (C-1), 114.0 (C-2), 108.2 (C-3), 49.9 (CH_2_-pip), 49.2 (CH_2_-pip), 33.9 (CH_2_). Anal. calcd for C_21_H_19_ClN_4_: C, 69.51; H, 5.28; N, 15.44. Found: C, 69.67; H, 5.07; N, 15.30.

##### 4-[4-(6-Chloro-5-trifluoromethylpyridin-2-yl)piperazinyl]pyrrolo[1,2-*a*]quinoxaline (1q)

White crystals (43%); mp 146 °C. ^1^H NMR (CDCl_3_) *δ* 7.88–7.86 (m, 1H, H-1), 7.77–7.73 (m, 2H, H-9 and H-6), 7.72 (d, 1H, *J* = 8.70 Hz, H-4′), 7.39–7.29 (m, 2H, H-7 and H-8), 6.86–6.80 (m, 2H, H-2 and H-3), 6.51 (d, 1H, *J* = 8.70 Hz, H-3′), 4.00–3.97 (m, 4H, CH_2_-pip), 3.89–3.86 (m, 4H, CH_2_-pip). ^13^C NMR (CDCl_3_) *δ* 160.6 (C-2′), 153.7 (C-4), 149.0 (q, *J* = 1.6 Hz, C-6′), 139.0 (q, *J* = 4.5 Hz, C-4′), 137.2 (C-5a), 128.9 (C-7), 127.2 (C-3a), 126.7 (C-9), 125.7 (C-6), 124.7 (q, *J* = 269.0 Hz, CF_3_), 121.3 (C-9a), 116.0 (C-8), 114.7 (C-1), 114.1 (C-2), 113.8 (q, *J* = 32.2 Hz, C-5′), 108.2 (C-3), 104.5 (C-3′), 48.6 (CH_2_-pip), 45.7 (CH_2_-pip). Anal. calcd for C_21_H_17_ClF_3_N_5_: C, 58.40; H, 3.97; N, 16.22. Found: C, 58.28; H, 4.07; N, 16.12.

##### 7-Methoxy-4-[4-(2-trifluoromethylphenethyl)piperazinyl]pyrrolo[1,2-*a*]quinoxaline (1r)

Yellow oil (79%). ^1^H NMR (CDCl_3_) *δ* 7.78–7.74 (m, 1H, H-1), 7.65 (d, 1H, *J* = 7.80 Hz, H-3′), 7.64 (d, 1H, *J* = 8.00 Hz, H-9), 7.51 (t, 1H, *J* = 7.80 Hz, H-5′), 7.43 (d, 1H, *J* = 7.80 Hz, H-6′), 7.33 (t, 1H, *J* = 7.80 Hz, H-4′), 7.20 (d, 1H, *J* = 2.60 Hz, H-6), 6.91 (dd, 1H, *J* = 8.00 and 2.60 Hz, H-8), 6.80–6.78 (m, 1H, H-3), 6.76–6.74 (m, 1H, H-2), 3.91–3.84 (m, 7H, CH_3_O and CH_2_-pip), 3.13–3.07 (m, 2H, CH_2_), 2.84–2.74 (m, 6H, CH_2_ and CH_2_-pip). ^13^C NMR (CDCl_3_) *δ* 158.7 (C-7), 154.3 (C-4), 140.3 (q, *J* = 3.2 Hz, C-1′), 138.7 (C-5a), 133.1 (q, *J* = 0.8 Hz, C-6′), 133.0 (C-5′), 130.0 (q, *J* = 29.4 Hz, C-2′), 127.6 (C-9), 127.3 (q, *J* = 5.6 Hz, C-3′), 126.0 (q, *J* = 272.2 Hz, CF_3_), 121.5 (C-3a), 121.1 (C-9a), 115.5 (C-4′), 115.4 (C-8), 113.9 (C-1), 113.5 (C-2), 110.7 (C-3), 107.9 (C-6), 61.6 (NCH_2_), 57.0 (CH_3_O), 54.5 (CH_2_-pip), 49.4 (CH_2_-pip), 31.4 (CH_2_). Anal. calcd for C_25_H_25_F_3_N_4_O: C, 66.07; H, 5.54; N, 12.33. Found: C, 65.94; H, 5.67; N, 12.20.

##### 8-Methoxy-4-[4-(2-trifluoromethylphenethyl)piperazinyl]pyrrolo[1,2-*a*]quinoxaline (1s)

Yellow oil (64%). ^1^H NMR (CDCl_3_) *δ* 7.73–7.71 (m, 1H, H-1), 7.66 (d, 1H, *J* = 8.70 Hz, H-6), 7.65 (d, 1H, *J* = 7.60 Hz, H-3′), 7.48 (t, 1H, *J* = 7.60 Hz, H-5′), 7.40 (d, 1H, *J* = 7.60 Hz, H-6′), 7.31 (t, 1H, *J* = 7.60 Hz, H-4′), 7.19 (d, 1H, *J* = 2.40 Hz, H-9), 6.95 (dd, 1H, *J* = 8.70 and 2.40 Hz, H-7), 6.79–6.77 (m, 2H, H-2 and H-3), 3.90 (s, 3H, CH_3_O), 3.79–3.78 (m, 4H, CH_2_-pip), 3.11–3.06 (m, 2H, CH_2_), 2.80–2.69 (m, 6H, CH_2_ and CH_2_-pip). ^13^C NMR (CDCl_3_) *δ* 158.2 (C-8), 152.9 (C-4), 140.2 (q, *J* = 1.65 Hz, C-1′), 133.2 (q, *J* = 0.9 Hz, C-6′), 133.1 (C-5′), 131.6 (C-5a), 130.1 (q, *J* = 29.3 Hz, C-2′), 130.0 (C-6), 127.8 (C-3a), 127.6 (C-4′), 127.3 (q, *J* = 5.6 Hz, C-3′), 126.0 (q, *J* = 272.1 Hz, CF_3_), 121.9 (C-9a), 115.4 (C-7), 114.0 (C-1), 113.6 (C-2), 107.8 (C-3), 99.3 (C-9), 61.7 (NCH_2_), 57.1 (CH_3_O), 54.5 (CH_2_-pip), 49.7 (CH_2_-pip), 31.4 (CH_2_). Anal. calcd for C_25_H_25_F_3_N_4_O: C, 66.07; H, 5.54; N, 12.33. Found: C, 66.20; H, 5.44; N, 12.46.

##### Ethyl 4-[4-(2-trifluoromethylphenethyl)piperazinyl]pyrrolo[1,2-*a*]quinoxaline-2-carboxylate (1t)

Pale-yellow crystals (66%); mp 118 °C. ^1^H NMR (CDCl_3_) *δ* 8.33 (s, 1H, H-1), 7.75 (d, 1H, *J* = 8.00 Hz, H-9), 7.67 (d, 1H, *J* = 8.00 Hz, H-6), 7.64 (d, 1H, *J* = 7.50 Hz, H-3′), 7.49 (t, 1H, *J* = 7.50 Hz, H-5′), 7.43 (d, 1H, *J* = 7.50 Hz, H-6′), 7.41–7.26 (m, 3H, H-7, H-8 and H-4′), 7.19 (s, 1H, H-3), 4.39 (q, 2H, *J* = 7.20 Hz, OCH_2_), 3.89–3.87 (m, 4H, CH_2_-pip), 3.10–3.05 (m, 2H, CH_2_), 2.79–2.69 (m, 6H, CH_2_ and CH_2_-pip), 1.42 (t, 2H, *J* = 7.20 Hz, CH_3_). ^13^C NMR (CDCl_3_) *δ* 165.7 (CO), 153.6 (C-4), 140.2 (C-1′), 137.9 (C-5a), 133.1 (C-5′), 133.0 (C-7), 130.0 (q, *J* = 30.0 Hz, C-2′), 129.0 (C-9), 127.7 (C-6), 127.6 (C-6′), 127.3 (q, *J* = 5.6 Hz, C-3′), 126.3 (C-3a), 126.0 (q, *J* = 272.2 Hz, CF_3_), 125.6 (C-4′), 121.9 (C-9a), 120.5 (C-2), 119.0 (C-8), 114.9 (C-1), 109.2 (C-3), 61.9 (OCH_2_), 61.6 (NCH_2_), 54.4 (CH_2_-pip), 49.2 (CH_2_-pip), 31.4 (CH_2_), 15.9 (CH_3_). Anal. calcd for C_27_H_27_F_3_N_4_O_2_: C, 65.31; H, 5.48; N, 11.28. Found: C, 65.39; H, 5.29; N, 11.42.

##### 2-Phenyl-4-[4-(2-trifluoromethylphenethyl)piperazinyl]pyrrolo[1,2-*a*]quinoxaline (1u)

Pale-yellow crystals (67%); mp 136 °C. ^1^H NMR (CDCl_3_) *δ* 8.08 (s, 1H, H-1), 7.77 (d, 1H, *J* = 8.10 Hz, H-9), 7.74–7.68 (m, 3H, H-6, H-2′′ and H-6′′), 7.66 (d, 1H, *J* = 7.50 Hz, H-3′), 7.51 (t, 1H, *J* = 7.50 Hz, H-5′), 7.49–7.28 (m, 7H, H-4′, H-6′, H-7, H-8, H-3′′, H-4′′ and H-5′′), 7.06 (s, 1H, H-3), 3.93–3.90 (m, 4H, CH_2_-pip), 3.13–3.07 (m, 2H, CH_2_), 2.84–2.72 (m, 6H, CH_2_ and CH_2_-pip). ^13^C NMR (CDCl_3_) *δ* 153.4 (C-4), 140.2 (C-1′′), 137.3 (C-5a), 135.7 (C-1′), 133.4 (C-5′′), 133.3 (C-7), 130.3 (C-3′ and C-5′), 129.9 (q, *J* = 29.3 Hz, C-2′′), 129.1 (C-9), 128.4 (C-4′), 128.0 (C-6), 127.6 (C-3a), 127.5 (C-2′ and C-6′), 127.4 (C-6′′), 127.0 (C-2), 126.7 (C-3′′), 125.9 (C-8), 125.7 (q, *J* = 272.2 Hz, CF_3_), 125.6 (C-4′′), 122.2 (C-9a), 114.7 (C-2), 112.6 (C-1), 105.7 (C-3), 61.2 (NCH_2_), 53.9 (CH_2_-pip), 48.7 (CH_2_-pip), 30.7 (CH_2_). Anal. calcd for C_30_H_27_F_3_N_4_: C, 71.98; H, 5.44; N, 11.19. Found: C, 72.06; H, 5.53; N, 11.35.

##### 2-Phenyl-4-[4-(3-trifluoromethylbenzyl)piperazinyl]pyrrolo[1,2-*a*]quinoxaline (1v)

Yellow oil (54%). ^1^H NMR (CDCl_3_) *δ* 8.07 (s, 1H, H-1), 7.76 (d, 1H, *J* = 7.80 Hz, H-9), 7.73–7.69 (m, 4H, H-6, H-4′, H-2′′ and H-6′′), 7.63–7.56 (m, 2H, H-7 and H-8), 7.51–7.43 (m, 3H, H-2′, H-3′′, H-5′′), 7.37–7.28 (m, 3H, H-5′, H-6′ and H-4′′), 7.04 (s, 1H, H-3), 3.89–3.86 (m, 4H, CH_2_-pip), 3.67 (s, 2H, NCH_2_), 2.71–2.69 (m, 4H, CH_2_-pip). ^13^C NMR (CDCl_3_) *δ* 153.8 (C-4), 140.3 (C-1′′), 137.4 (C-5a), 135.8 (C-1′), 133.9 (C-6′′), 132.0 (q, *J* = 32.0 Hz, C-3′′), 130.3 (C-3′ and C-5′), 130.2 (C-7), 129.8 (C-3a), 128.9 (C-9), 128.3 (C-4′), 127.4 (C-2′ and C-6′), 127.2 (C-5′′), 127.1 (q, *J* = 3.75 Hz, C-2′′), 126.9 (C-2), 126.7 (C-6), 125.6 (C-8), 125.5 (q, *J* = 3.9 Hz, C-4′′), 125.4 (q, *J* = 271.3 Hz, CF_3_), 122.3 (C-9a), 114.6 (C-2), 112.4 (C-1), 105.8 (C-3), 63.8 (NCH_2_), 54.4 (CH_2_-pip), 49.3 (CH_2_-pip). Anal. calcd for C_29_H_25_F_3_N_4_: C, 71.59; H, 5.18; N, 11.52. Found: C, 71.75; H, 5.08; N, 11.57.

##### 2-Phenyl-4-[4-(4-trifluoromethylbenzyl)piperazinyl]pyrrolo[1,2-*a*]quinoxaline (1w)

Yellow oil (76%). ^1^H NMR (CDCl_3_) *δ* 8.08 (d, 1H, *J* = 0.90 Hz, H-1), 7.79 (dd, 1H, *J* = 7.80 and 1.80 Hz, H-9), 7.71–7.67 (m, 3H, H-6, H-2′′ and H-6′′), 7.64–7.55 (m, 2H, H-7 and H-8), 7.48–7.42 (m, 2H, H-3′′ and H-5′′), 7.35–7.29 (m, 3H, H-2′, H-6′ and H-4′′), 7.02 (d, 1H, *J* = 0.90 Hz, H-3), 3.88–3.85 (m, 4H, CH_2_-pip), 3.68 (s, 2H, NCH_2_), 2.72–2.69 (m, 4H, CH_2_-pip). ^13^C NMR (CDCl_3_) *δ* 153.8 (C-4), 143.8 (q, *J* = 1.0 Hz, C-1′′), 137.5 (C-5a), 135.8 (C-1′), 130.7 (q, *J* = 28.3 Hz, C-4′′), 130.6 (C-7), 130.3 (C-3′ and C-5′), 129.7 (C-3a), 129.0 (C-9), 128.3 (C-4′), 127.4 (C-2′ and C-6′), 126.7 (C-6), 126.6 (q, *J* = 6.3 Hz, C-3′′ and C-5′′), 126.5 (q, *J* = 3.5 Hz, C-2′′ and C-6′′), 125.6 (q, *J* = 267.8 Hz, CF_3_), 125.5 (C-8), 122.4 (C-9a), 114.6 (C-2), 112.4 (C-1), 105.7 (C-3), 63.9 (NCH_2_), 54.5 (CH_2_-pip), 49.4 (CH_2_-pip). Anal. calcd for C_29_H_25_F_3_N_4_: C, 71.59; H, 5.18; N, 11.52. Found: C, 71.72; H, 5.04; N, 11.48.

### X-ray crystallography studies

Crystallographic data of compounds 1a and 1h were collected at 293 K with an Enraf-Nonius CAD-4 diffractometer with monochromatic Cu-Kα radiation (*l* = 1.54178 Å). The collected data were reduced using the NONIUS CAD4 software and all reflections were used for unit-cell refinement. Then the data were corrected for Lorentz and polarization effects and for empirical absorption correction.^51^ The structure was solved by direct methods Shelx 2013 (ref. 52) and refined using Shelx 2013^52^ suite of programs.

Colorless single crystal of 1a was obtained by slow evaporation from dichloromethane/methanol solution (70/30; v/v): triclinic, space group *P*1̄, *a* = 9.5233(14) Å, *b* = 10.8011(19) Å, *c* = 11.8823(16) Å, *α* = 107.688(11)°, *β* = 110.089(10)°, *γ* = 100.552(11)°, *V* = 1035.6(3) Å^3^, *Z* = 2, *δ* (calcd) = 1.259 Mg m^−3^, FW = 392.49 for C_26_H_24_N_4_, *F*(000) = 416. Colorless single crystal of 1h was obtained by slow evaporation from methanol/dichloromethane (20/80; v/v): triclinic, space group *P*1̄, *a* = 9.471(10) Å, *b* = 13.021(6) Å, *c* = 19.887(6) Å, *α* = 77.83(4)°, *β* = 81.76(8)°, *γ* = 87.25(6)°, *V* = 2372(3) Å^3^, *Z* = 2, *δ* (calcd) = 1.266 Mg m^−3^, FW = 904.29 for 2C_29_H_27_FN_4_, 0.1H_2_O, *F*(000) = 955.

Full crystallographic results have been deposited at the Cambridge Crystallographic Data Centre (CCDC-891809, CCDC-891808, respectively), UK, as ESI.†^53^

### Biological studies

#### Yeast strains and growth media

All the yeast strains were grown in yeast extract peptone-dextrose (YEPD) broth (HiMedia) and for agar plates, 2.5% (w/v) Bacto agar (Difco, BD Biosciences) was added to the medium. The *S. cerevisiae* strain used as a heterologous host for the expression of Cdr1 and Mdr1 proteins was AD1-8u^−^, provided by Richard D. Cannon, University of Otago, Dunedin, New Zealand. The host AD1-8u^−^ having seven major ABC transporters deleted was suitably modified to clone GFP-tagged Cdr1protein and its mutant variants.^54^ The yeast strains AD1-8u^−^, AD-CDR1, AD-MDR1 (ref. 55–57) were cultured at 30 °C. No trailing effect of the compound or fluconazole was observed and the false negatives were ruled out as we compare our experimental data with negative control that is AD1-8u^−^ strain (empty vector strain). 15% glycerol stocks of these strains were maintained in −80 °C storage that were freshly revived on YEPD before use.

#### Reagents and media

Nile Red (>98%), curcumin (purity ≥ 99.5%), and verapamil (purity ≥ 99%) were obtained from Sigma Chemical Co. Fluconazole (>98%) was obtained from Ranbaxy and [3*H*]-fluconazole (20 Ci mmol^−1^) was provided by Moravek Biochemicals and Radiochemicals. All routine chemicals were obtained from HiMedia and were of analytical grade.

#### Statistical analysis

Data are the means ± SD from duplicate samples of at least three independent experiments. Differences between the mean values were analyzed by Student's *t* test (GraphPad QuickCalcs: *t*, test calculator), and the results were considered as significant when *p* < 0.05.

#### Transport assays

Transport assays were implemented by following the accumulation of Nile Red by flow cytometry with a FACsort flow cytometer (Becton-Dickinson Immunocytometry Systems) in cells overexpressing MDR transporters CaCdr1p (AD-CDR1) or CaMdr1p (AD-MDR1). Briefly, the cells with an OD_600_ of 0.1 were inoculated, which were allowed to grow at 30 °C with shaking, until the OD_600_ reached 0.25. The cells were then harvested and resuspended as a 5% cell suspension in diluted medium (containing one part of YEPD and two parts of water). Nile Red was added to a final concentration of 7 μM, and the cells were incubated at 30 °C for 30 min in absence or presence of each inhibitor at a concentration 10-fold higher than substrate (70 μM). The cells were then harvested and 10 000 cells were analyzed in the acquisition. The analysis was performed using the CellQuest software (Becton Dickinson Immunocytometry Systems). Efflux of 100% was attributed to the cells not exposed to Nile Red and normalized with the efflux mediated *via* MDR transporters.

#### Confocal microscopy

Confocal imaging of GFP-tagged Cdr1p and Mdr1p was performed with a Bio-Rad confocal microscope (MRC 1024) with a 100× oil immersion. The cells were washed and resuspended in an appropriate volume of 50 mM HEPES (pH 7.0). The cells were placed on the glass slides, and a drop of antifade reagent (Fluoroguard high-performance antifade reagent, Bio-Rad, Hercules, CA, USA) was added to prevent photobleaching.^55^

#### Cytotoxicity and fractional inhibitory concentration index (FICI) determination

Yeast cells (10^4^) were seeded into 96-well plates in either absence or presence of varying concentrations of inhibitors (3–800 μM), and grown for 48 h at 30 °C. The optical density of each strain at 600 nm was measured for the cell growth. The growth in the absence of any inhibitor was considered as 100%, and the concentration producing 80% of cell growth inhibition was taken as the MIC_80_ value; the resistance index (RI) was calculated as the ratio between the MIC_80_ values determined for the strain overexpressing either CaCdr1p (AD-CDR1) or CaMdr1p (AD-MDR1) relative to that of the control strain (AD1-8u^−^). The interaction of the respective inhibitors with fluconazole was evaluated by the checkerboard method^43^ and was expressed as FICI. The ranges of concentrations used were 1.25–65 μM for fluconazole, and 3–800 μM for the inhibitors. FICI values were calculated as the sum of the FICs of each agent (fluconazole and inhibitors). The FIC of each agent was calculated as the MIC_80_ of the agent in combination divided by the MIC_80_ of the agent alone.

## Conflicts of interest

There are no conflicts to declare.

## Supplementary Material

RA-010-C9RA09348F-s001

RA-010-C9RA09348F-s002
