# Supplementary material for: Synthesis of new piperazinyl-pyrrolo[1,2-a]quinoxaline derivatives as inhibitors of Candida albicans multidrug transporters by a Buchwald–Hartwig cross-coupling reaction
Source: RSC Adv. 2020 Jan 15;10(5):2915–31. doi: 10.1039/c9ra09348f (PMC9048445; doi:10.1039/c9ra09348f)
Supplement: RA-010-C9RA09348F-s001 [file RA-010-C9RA09348F-s001.pdf]

Electronic Supplementary Material (ESI) for RSC Advances.
This journal is © The Royal Society of Chemistry 2020

Supplementary Information

Synthesis of new piperazinyl-pyrrolo[1,2-*a*]quinoxaline derivatives as inhibitors of *Candida albicans* multidrug transporters by a Buchwald-Hartwig cross-coupling reaction

Jean Guillon, ‡[a] Shweta Nim, ‡[b] Stéphane Moreau,[a] Luisa Ronga,[a] Solène Savrimoutou,[a] Elisabeth Thivet,[a] Mathieu Marchivie,[c] Attilio Di Pietro,[d] Rajendra Prasad[e] and Marc Le Borgne *[f]

[a]*Univ. Bordeaux, INSERM U1212 – UMR CNRS 5320, ARNA Laboratory, UFR des Sciences Pharmaceutiques, F-33076 Bordeaux cedex, France*

[b]*School of Life Sciences, Jawaharlal Nehru University, 110067 New Delhi, India*

[c]*CNRS, Univ. Bordeaux, Bordeaux INP, ICMCB, UMR 5026, F-33608 Pessac cedex, France*

[d]*DRMP group, IBCP, UMR 5086 (MMSB), CNRS/Lyon I University, 69367 Lyon, France*

[e]*Amity Institute of Integrative Sciences and Health, AMITY University, Education Valley, Gurgaon 122413, India*

[f]*Université de Lyon, Université Claude Bernard Lyon 1, Faculté de Pharmacie - ISPB, EA 4446 Bioactive Molecules and Medicinal Chemistry, SFR Santé Lyon-Est CNRS UMS3453 - INSERM US7, Lyon, France. E-mail:* marc.le-borgne@univ-lyon1.fr

‡ These authors contributed equally to this work.

† Electronic supplementary information (ESI) available. See DOI: 10.1039/xxxxxxxxxx

Table of Contents

Fig. S1 – S45 Spectral Data (1H and 13C RMN) for compounds **1a-w** ***S2-S24***

Table S1 Intrinsic cytotoxicity of piperazinyl-pyrrolo[1,2-*a*]quinoxaline derivatives **1a-w** and **2a-f** ***S25***

Table S2 Ability of piperazinyl-pyrrolo[1,2-*a*]quinoxaline derivatives to sensitize yeast growth to FLC cytotoxicity ***S27***

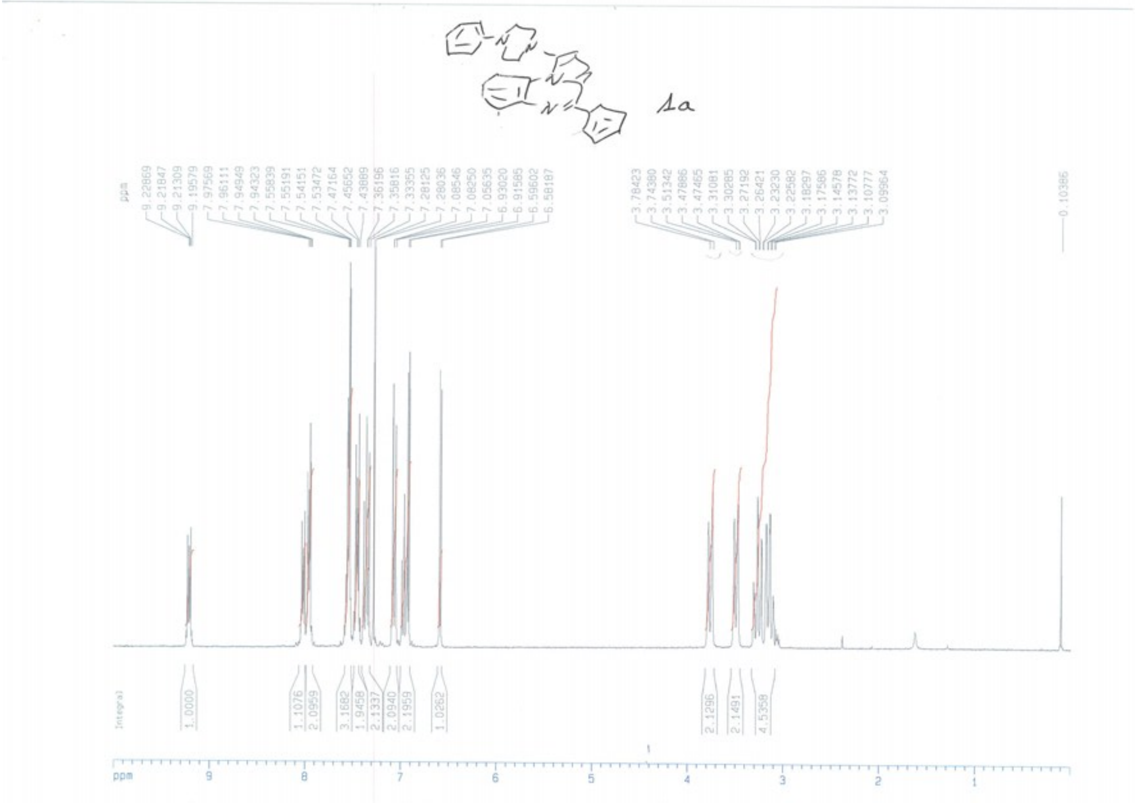

Fig. S1. ^{1}H NMR spectrum of **1a**.

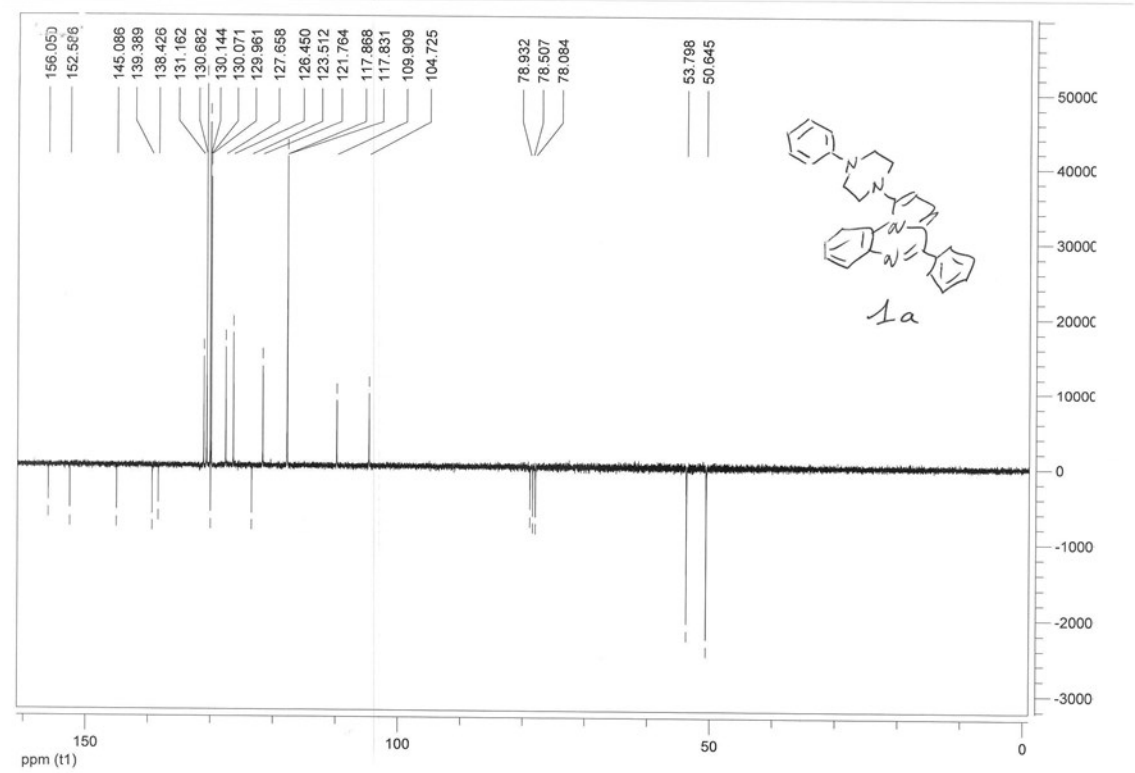

Fig. S2. ^{13}C NMR spectrum of **1a**.

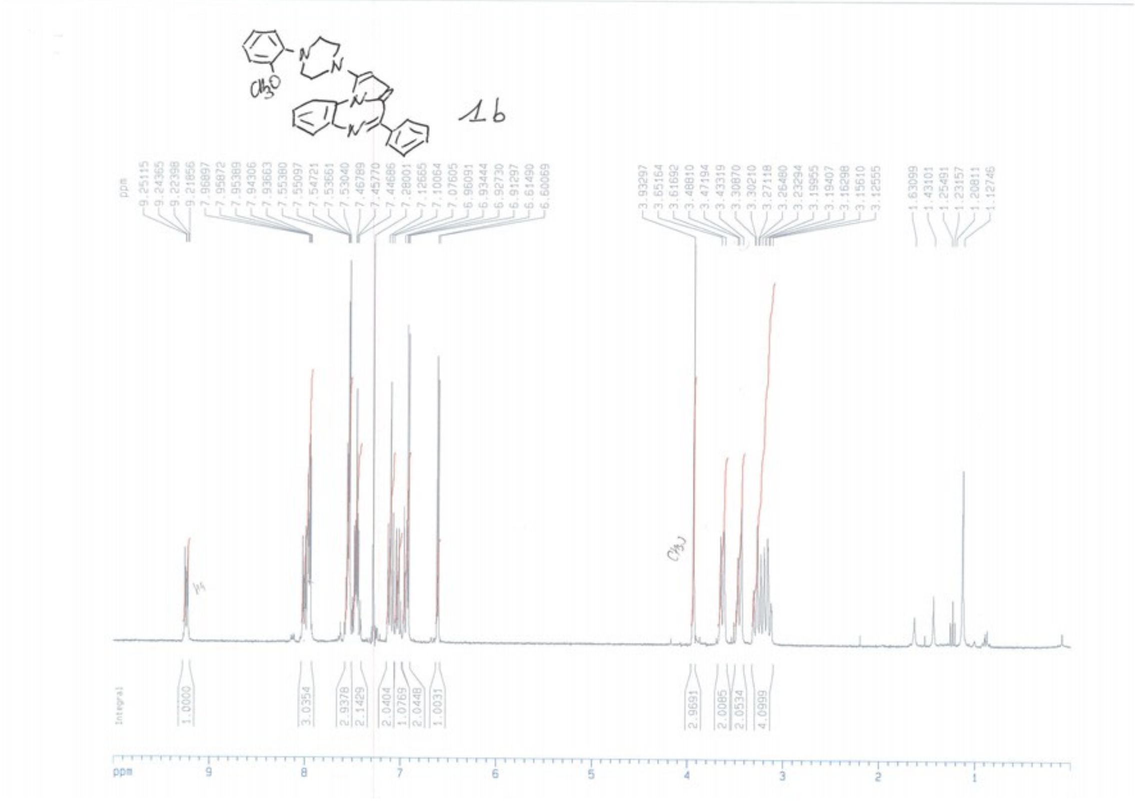

Fig. S3. ^{1}H NMR spectrum of **1b**.

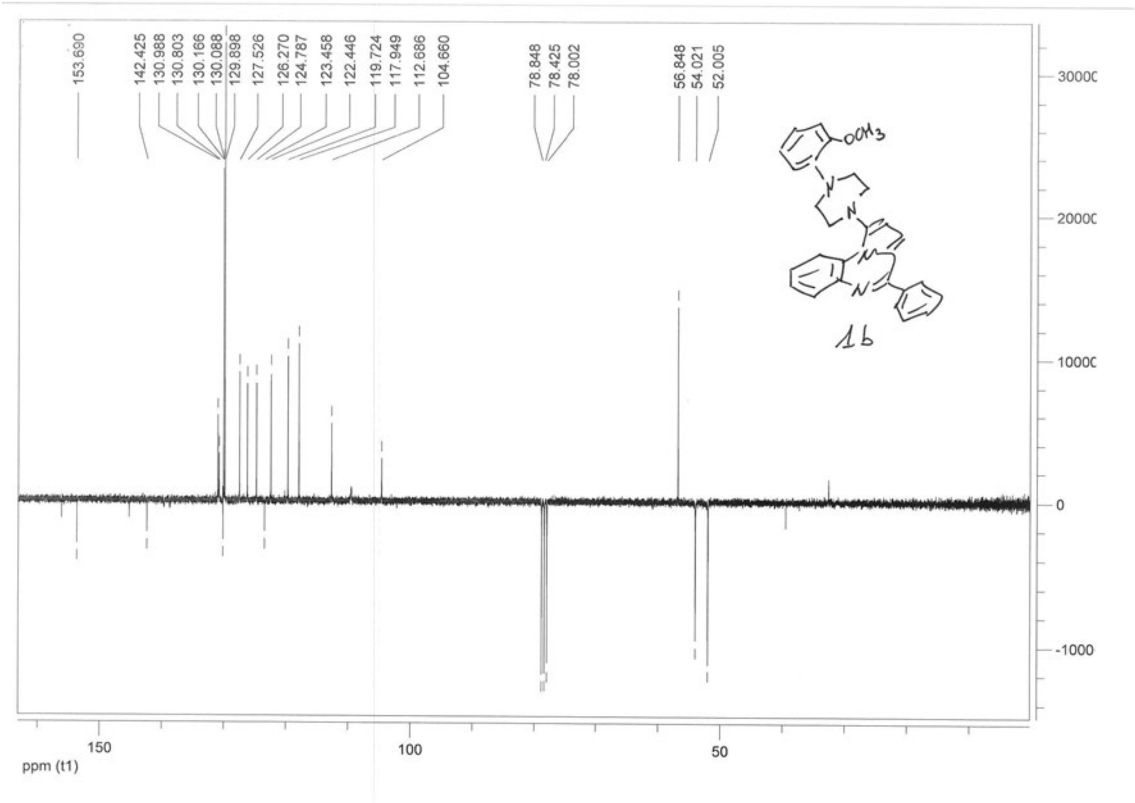

Fig. S4. ^{13}C NMR spectrum of **1b**.

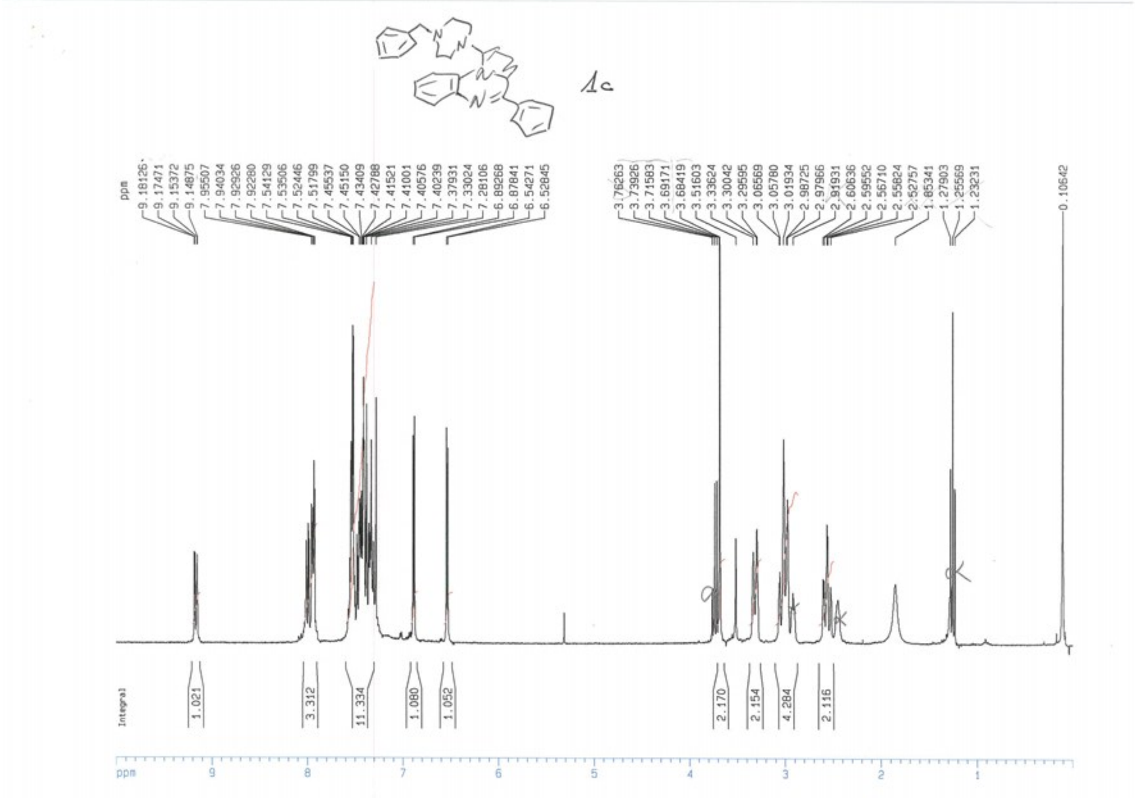

Fig. S5. ^{1}H NMR spectrum of **1c**.

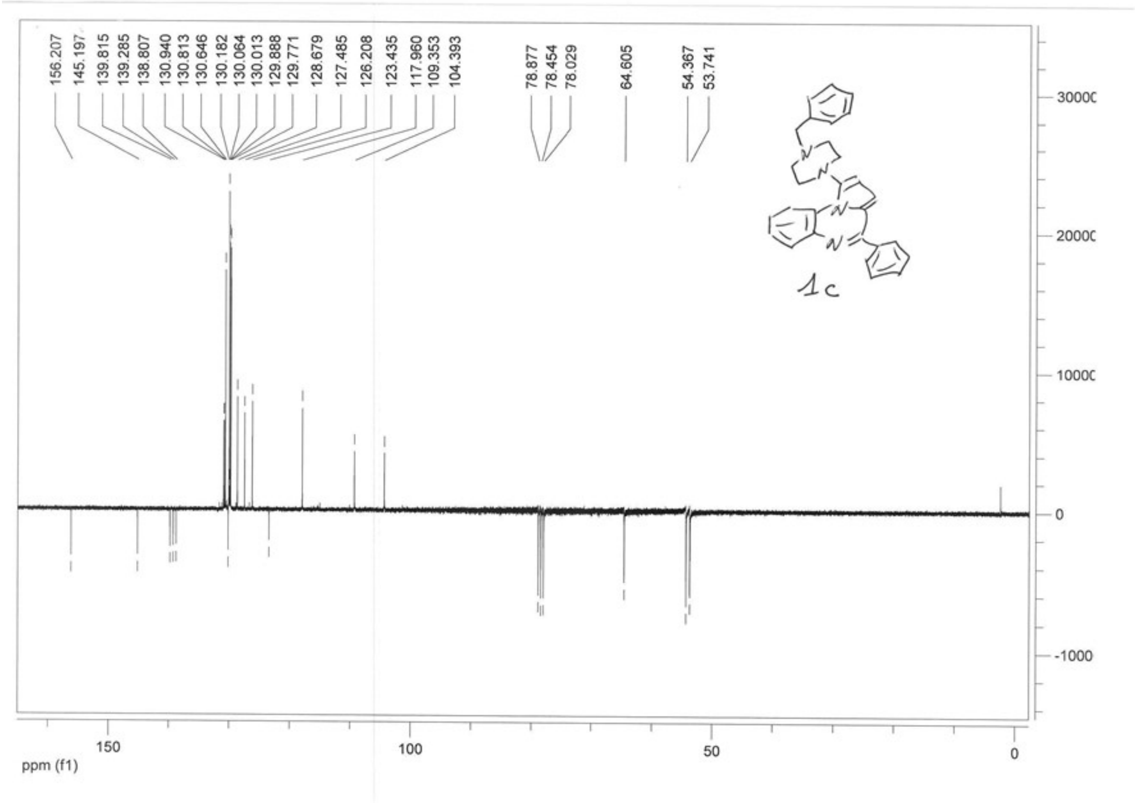

Fig. S6. ^{13}C NMR spectrum of **1c**.

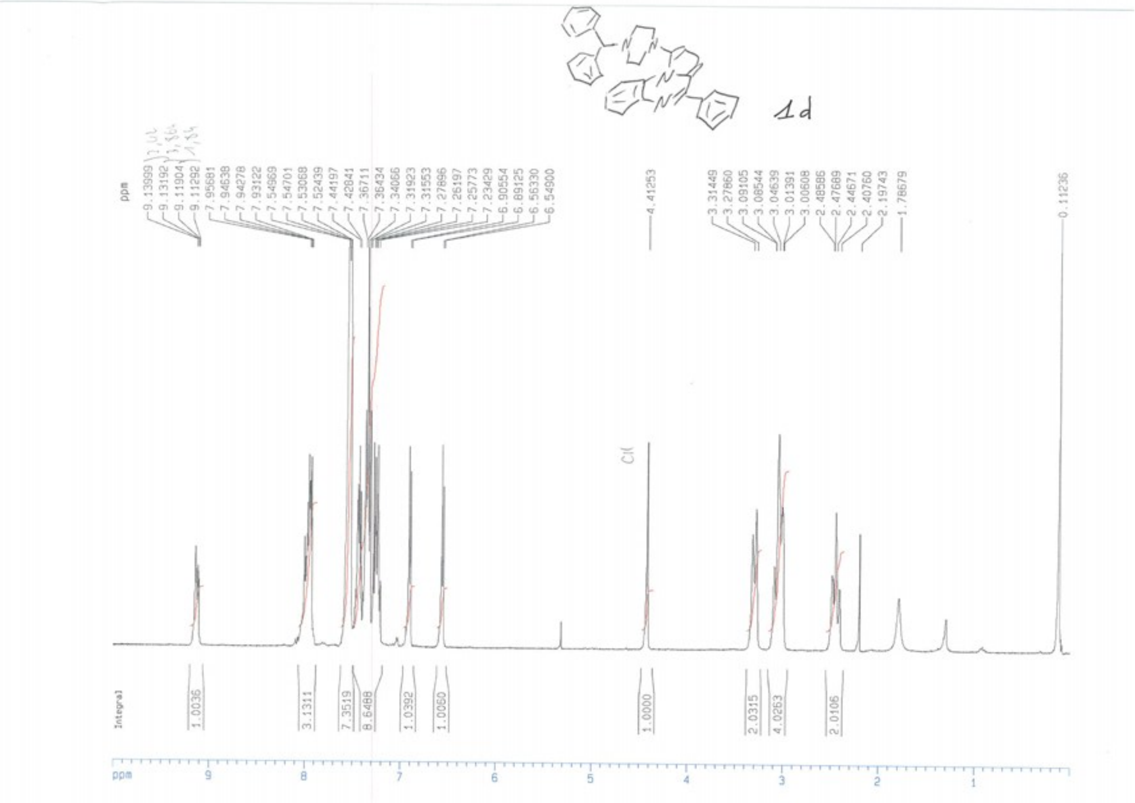

Fig. S7. ^{1}H NMR spectrum of **1d**.

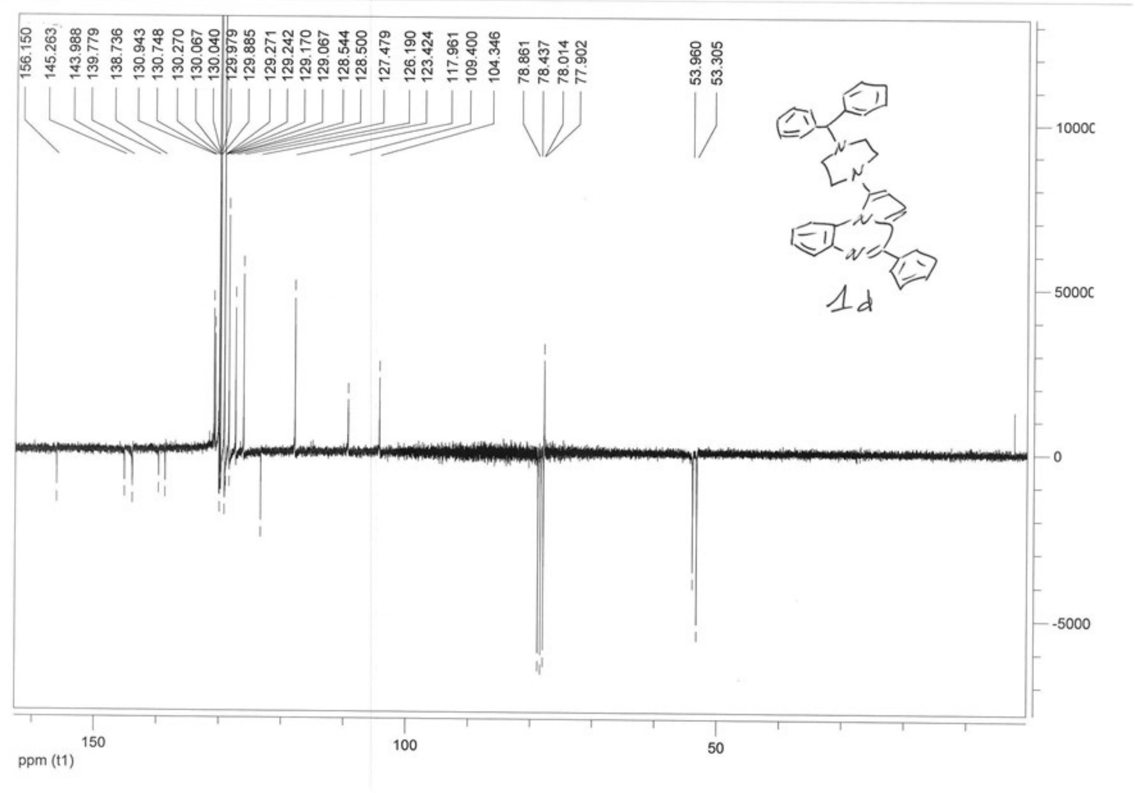

Fig. S8. ^{13}C NMR spectrum of **1d**.

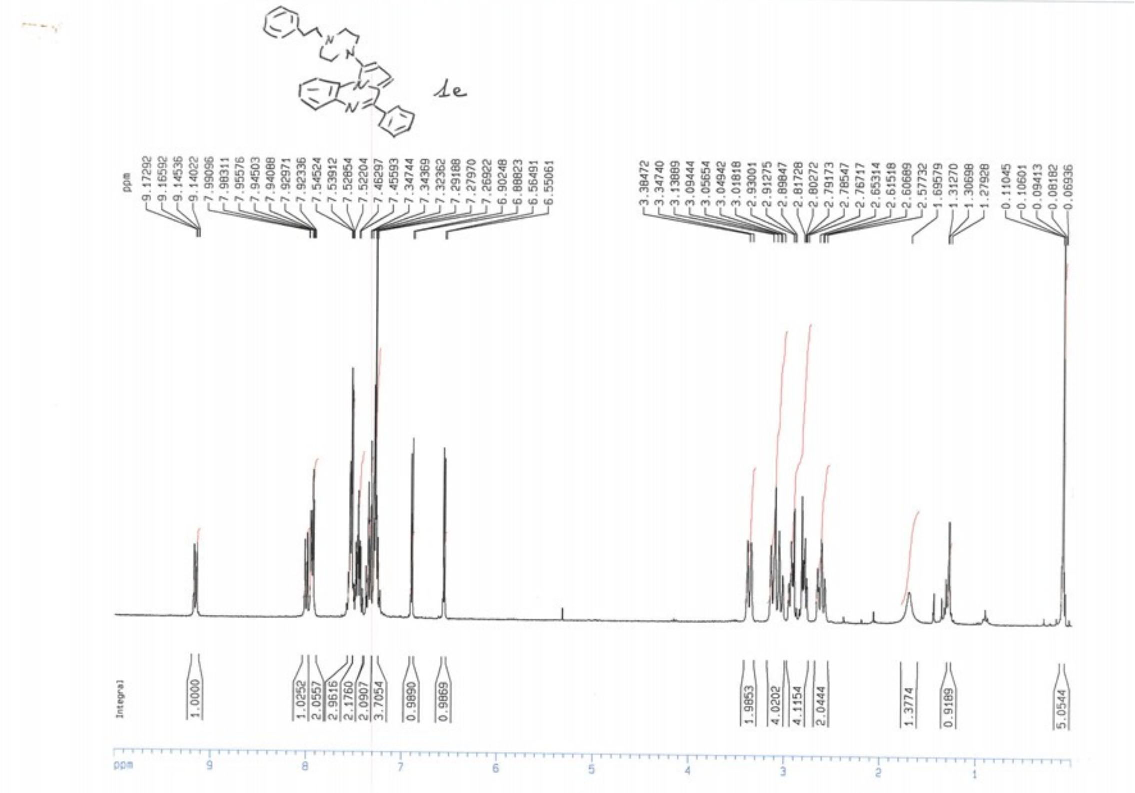

Fig. S9. ^{1}H NMR spectrum of **1e**.

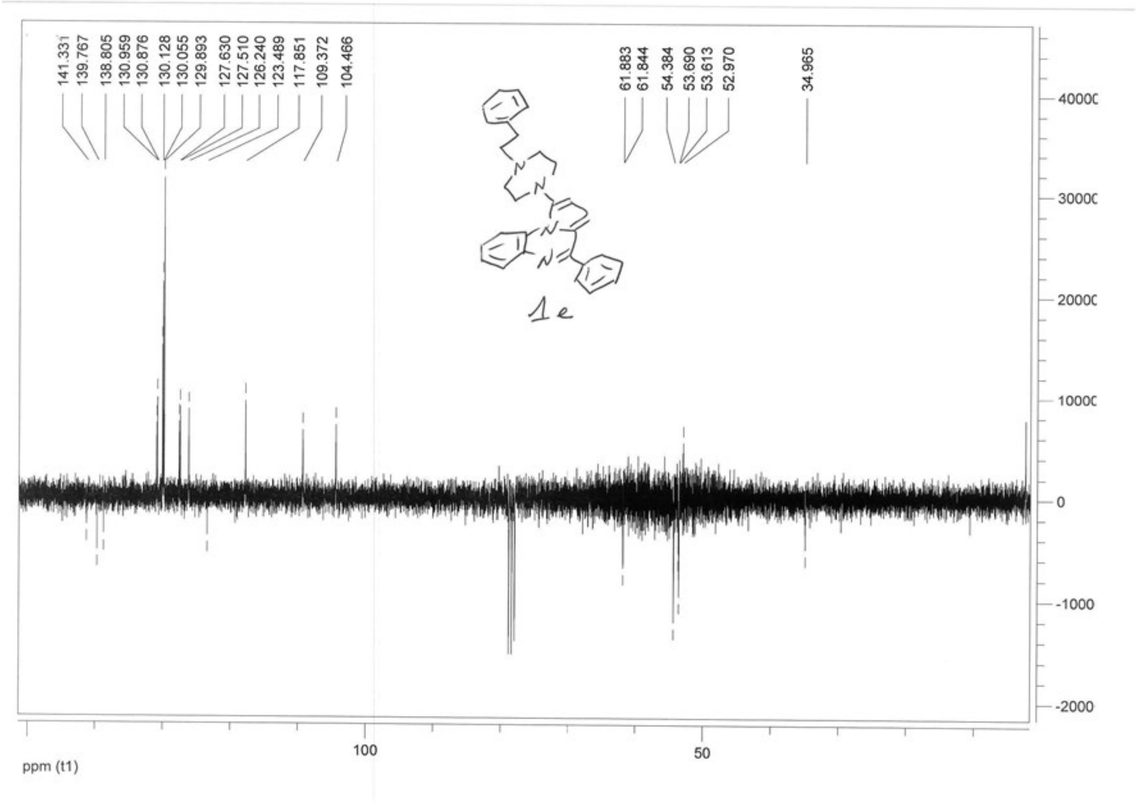

Fig. S10. ^{13}C NMR spectrum of **1e**.

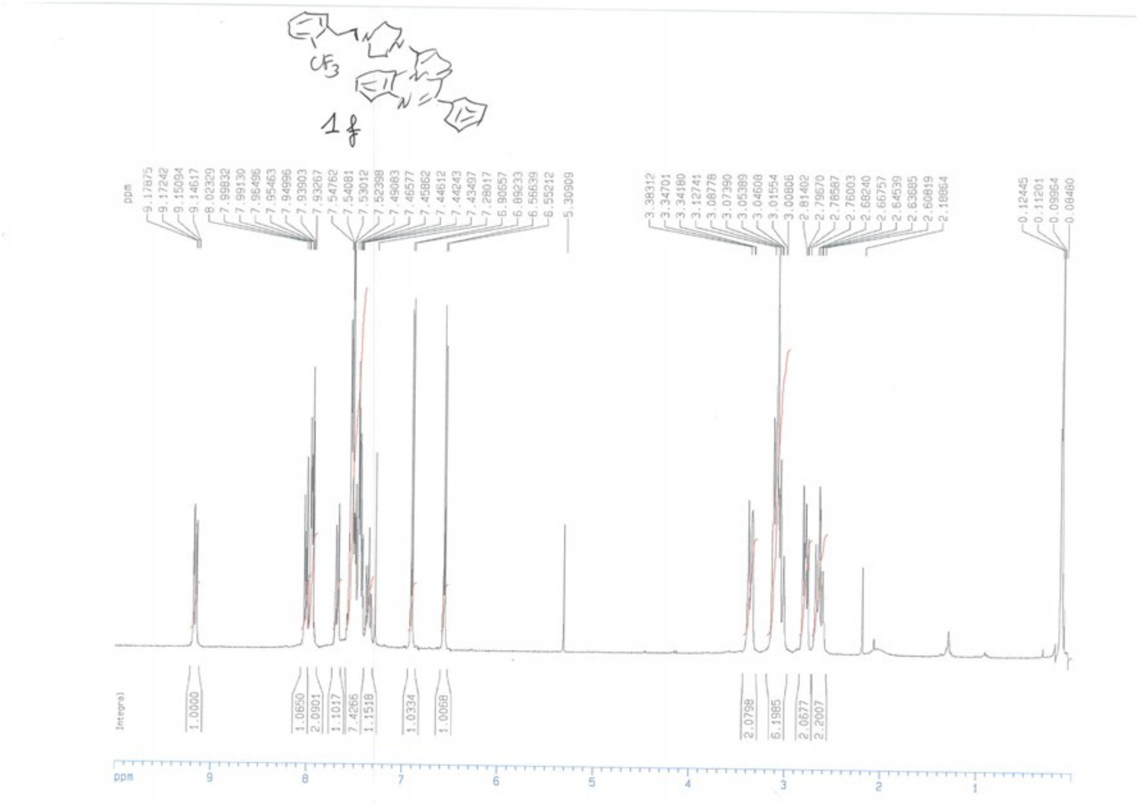

Fig. S11. ^{1}H NMR spectrum of **1f**.

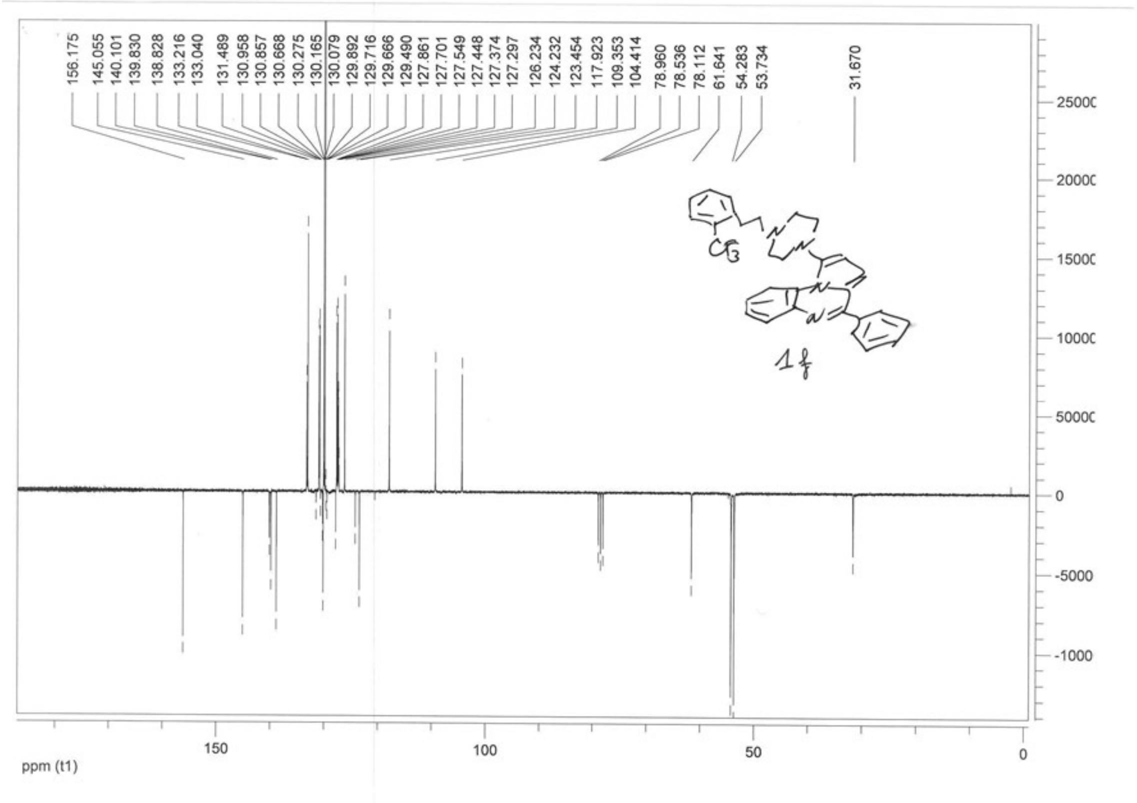

Fig. S12. ^{13}C NMR spectrum of **1f**.

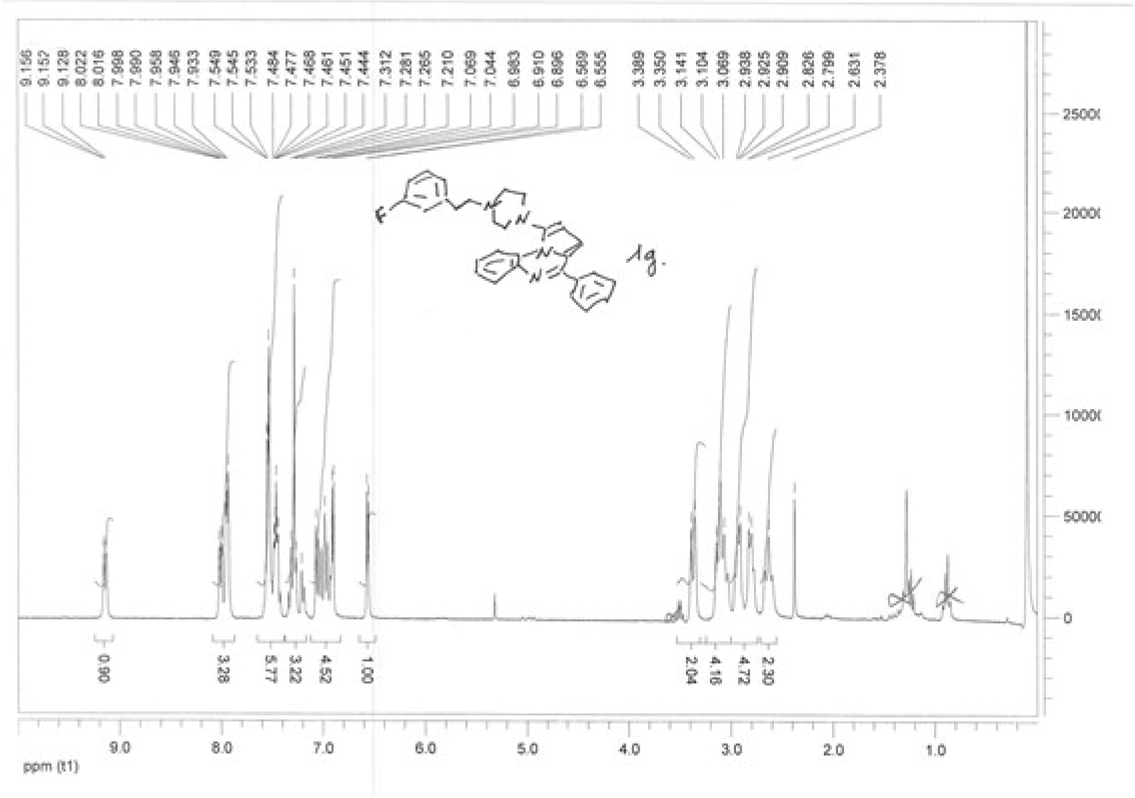

Fig. S13. ^{1}H NMR spectrum of **1g**.

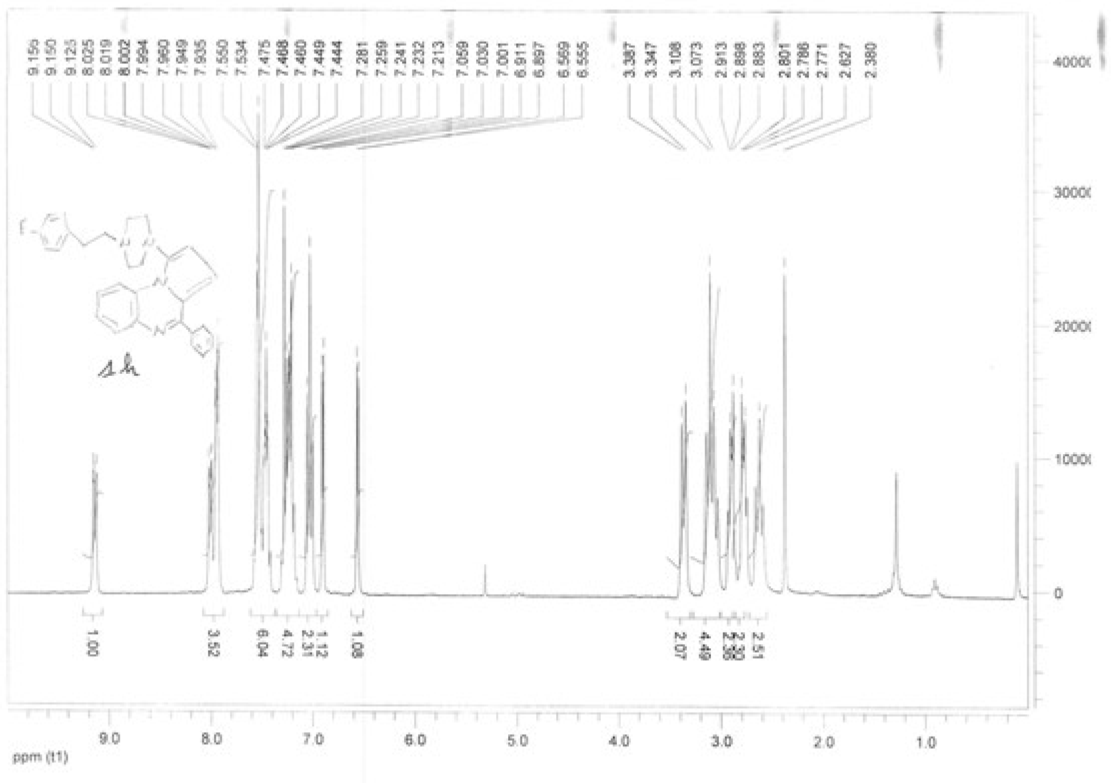

Fig. S14. ^{1}H NMR spectrum of **1h**.

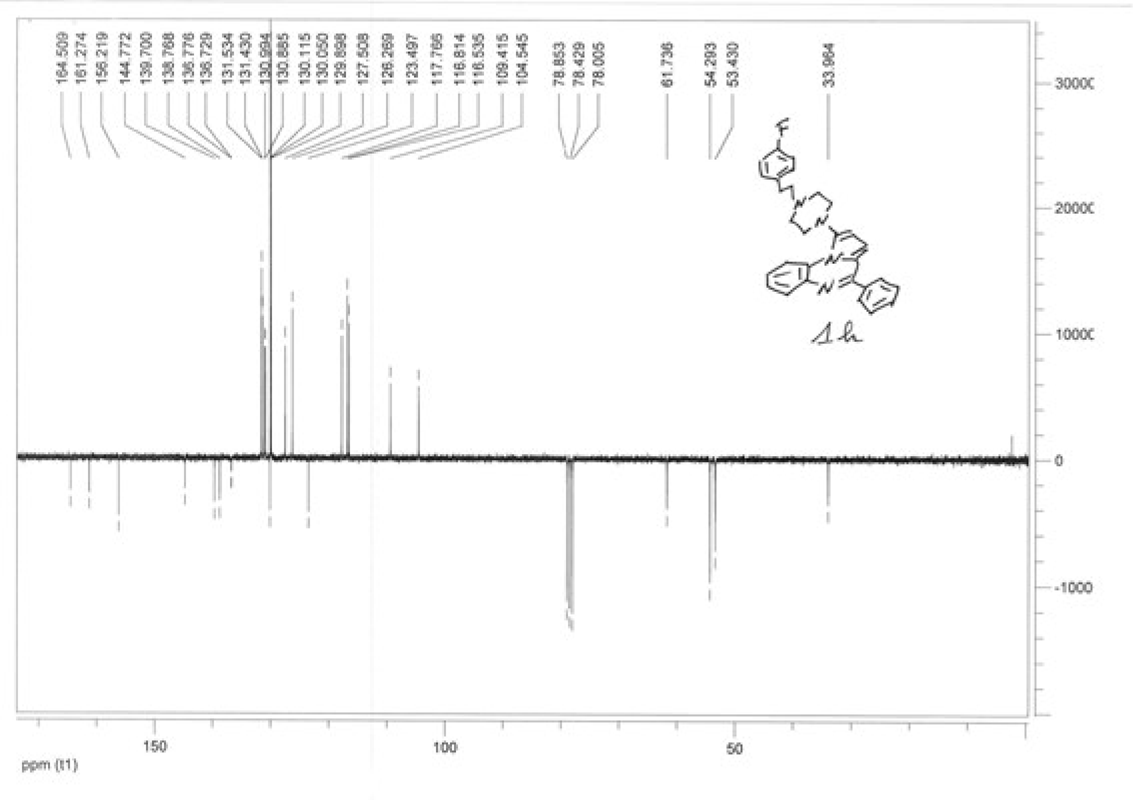

Fig. S15. ^{13}C NMR spectrum of **1h**.

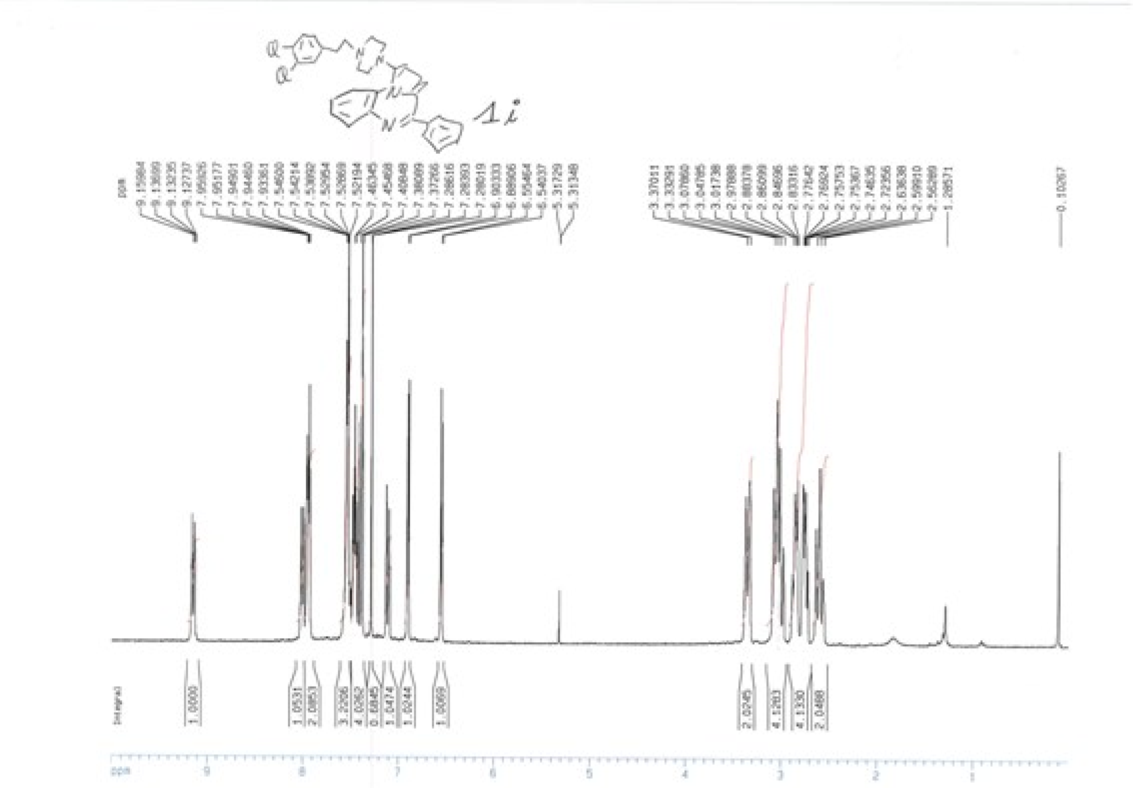

Fig. S16. ^{1}H NMR spectrum of **1i**.

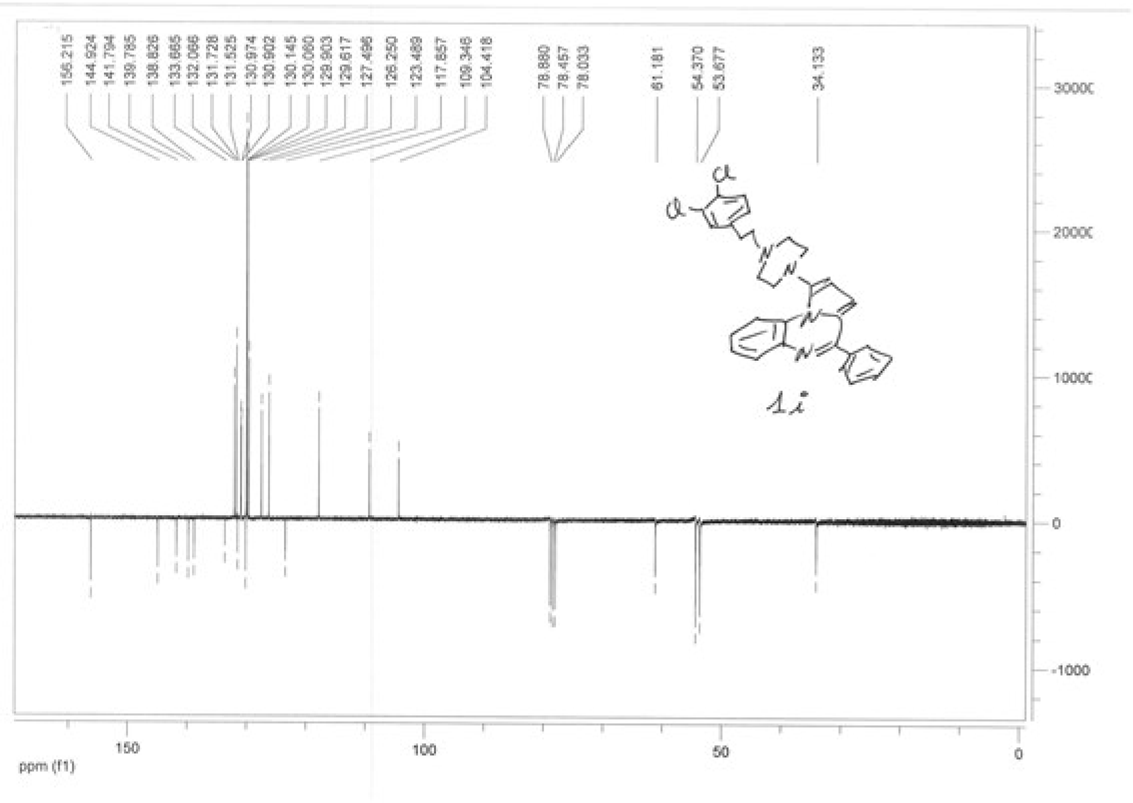

Fig. S17. ^{13}C NMR spectrum of **1i**.

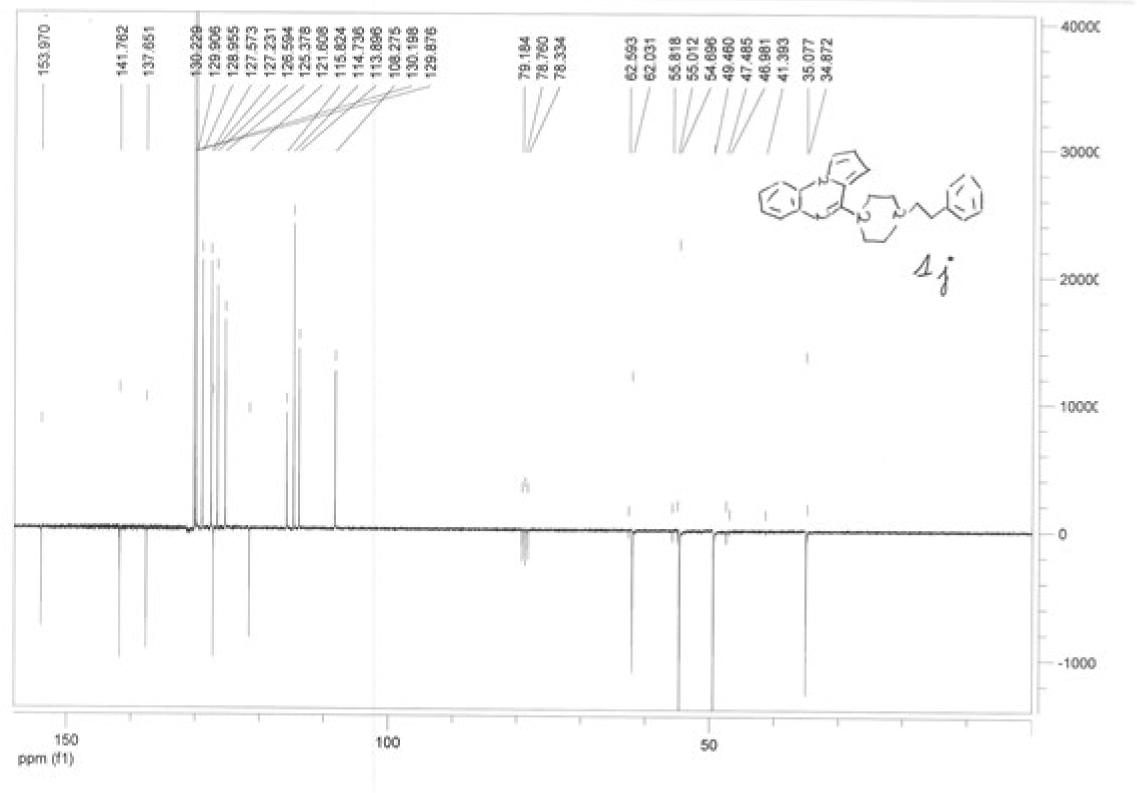

Fig. S18. ^{1}H NMR spectrum of **1j**.

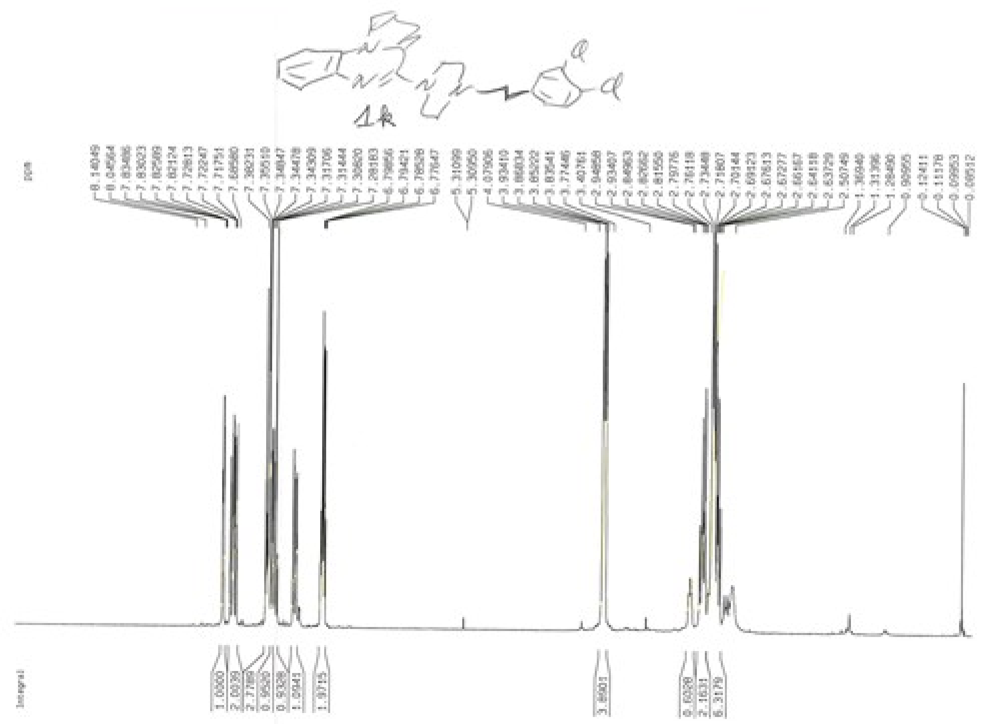

Fig. S19. ^{13}C NMR spectrum of **1j**.

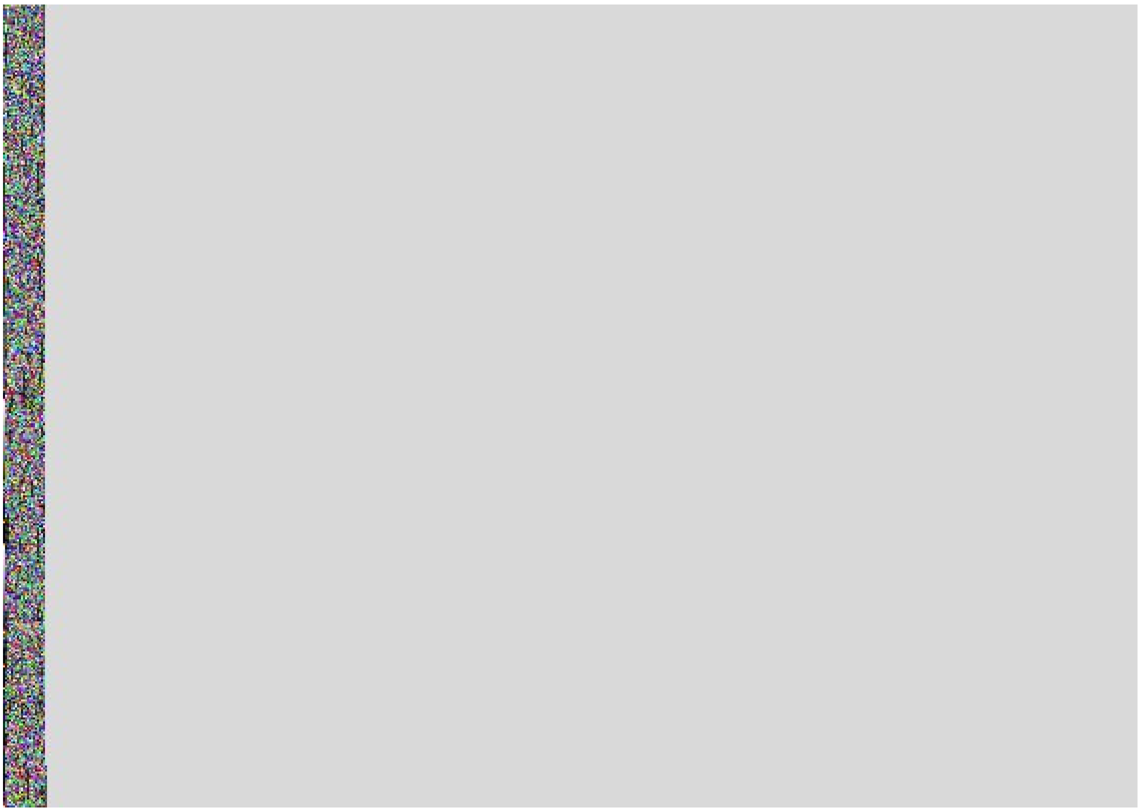

Fig. S20. ^{1}H NMR spectrum of **1k**.

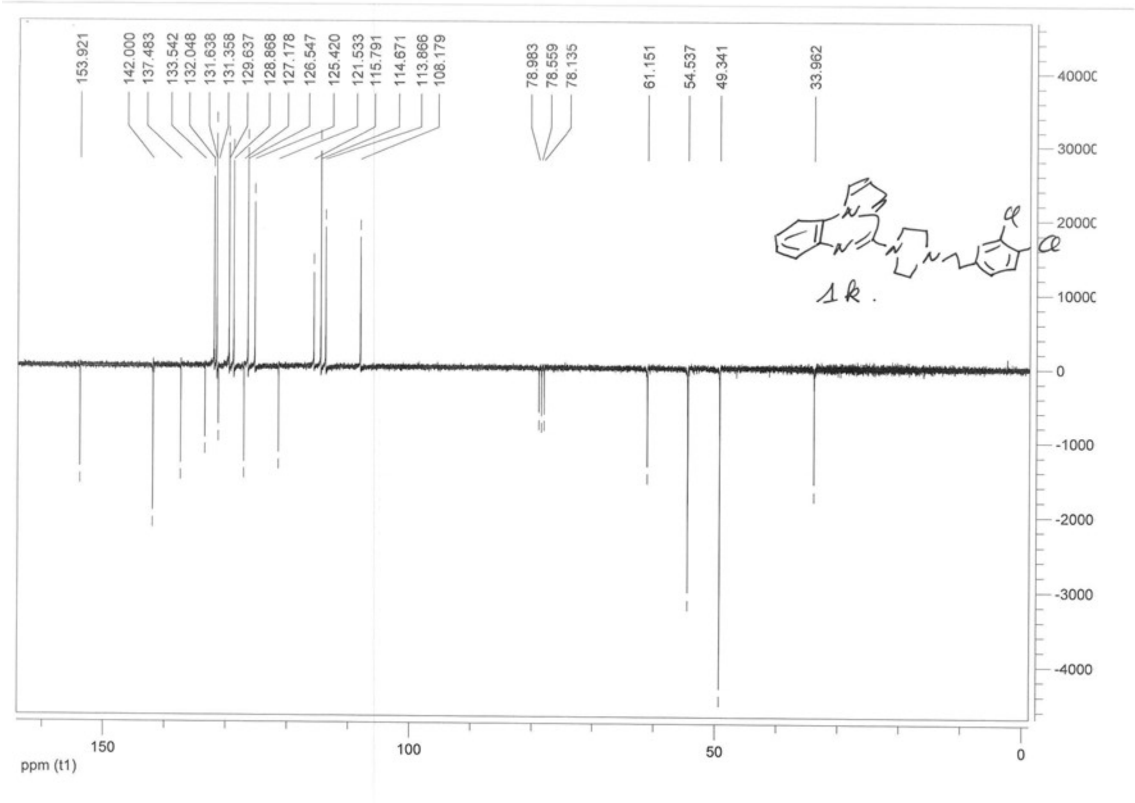

Fig. S21. ^{13}C NMR spectrum of **1k**.

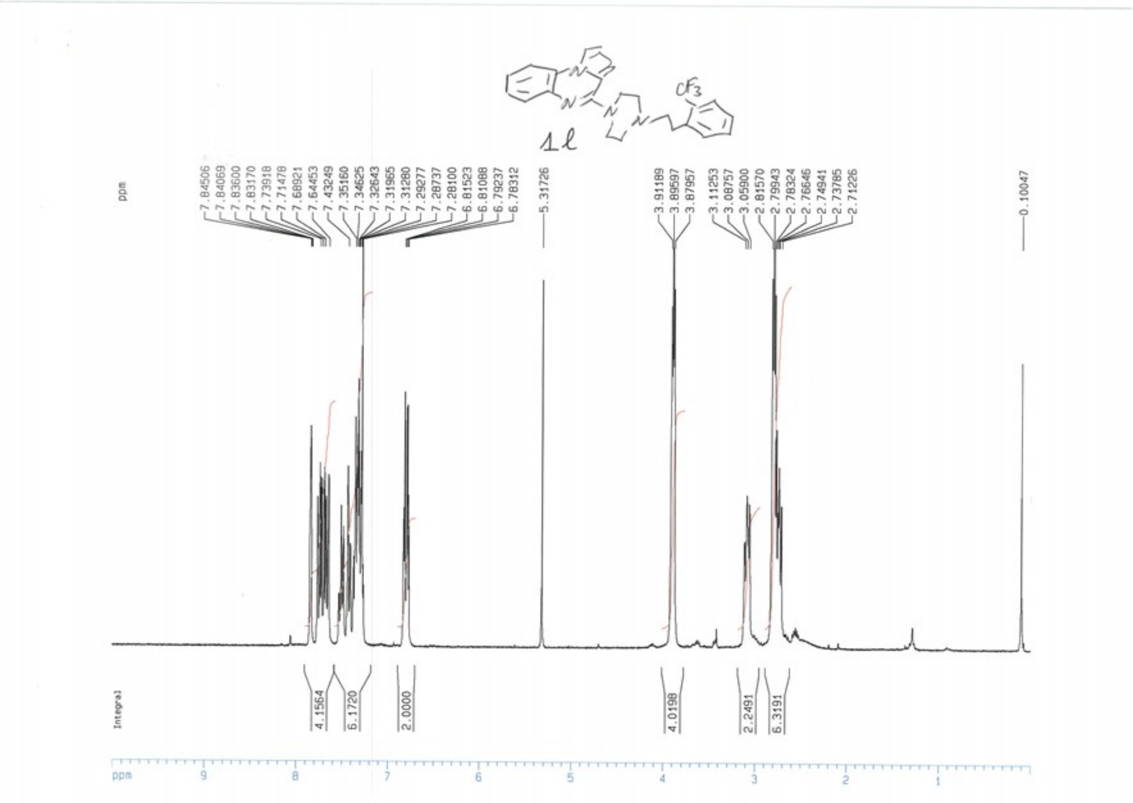

Fig. S22. ^{1}H NMR spectrum of **1l**.

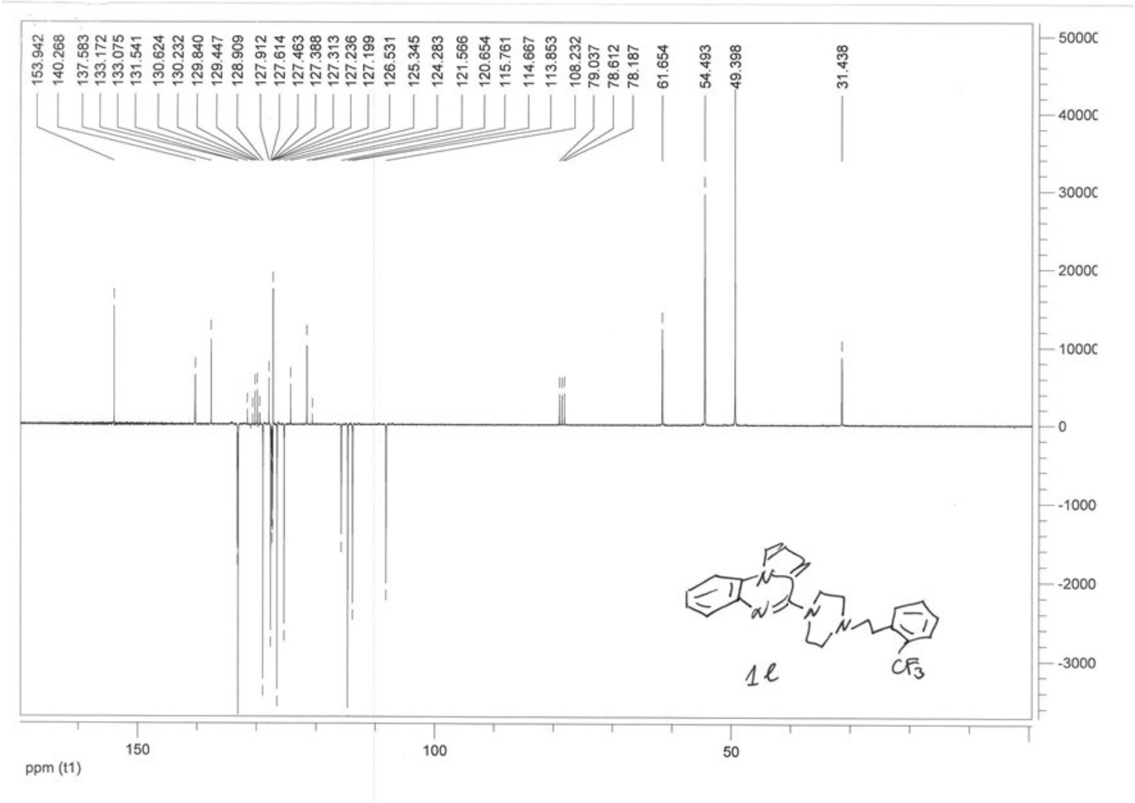

Fig. S23. ^{13}C NMR spectrum of **1l**.

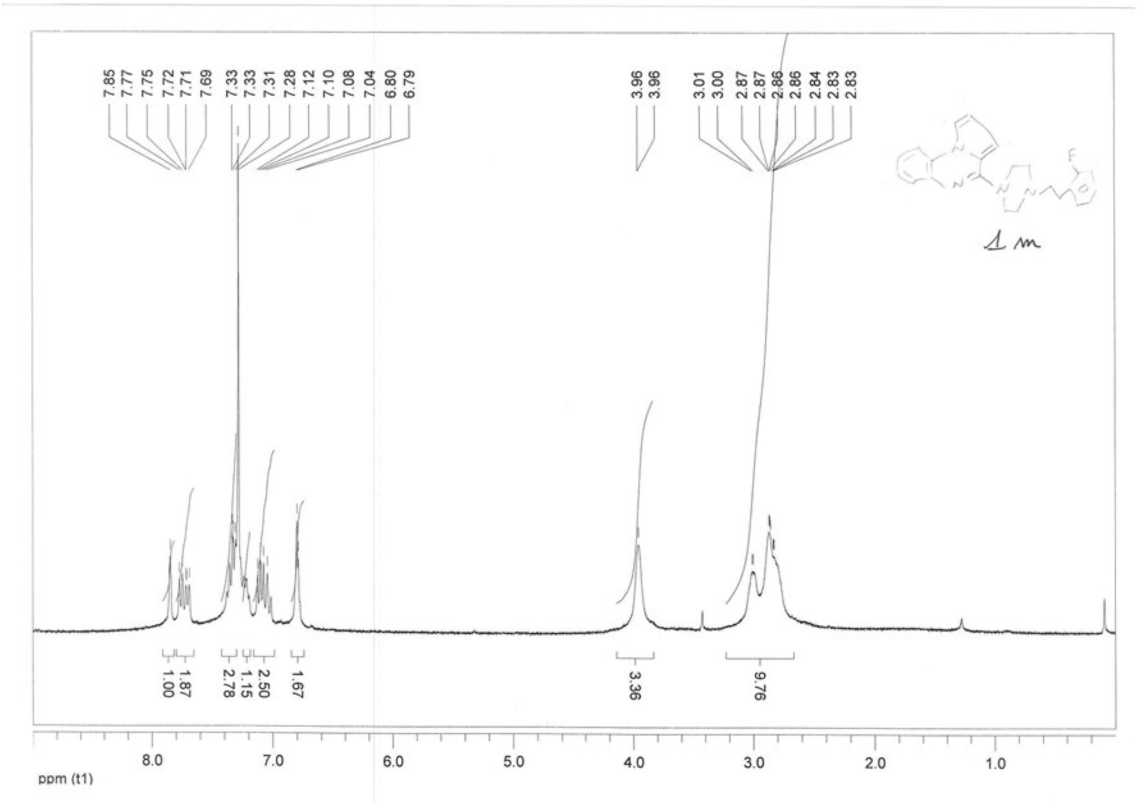

Fig. S24. ^{1}H NMR spectrum of **1m**.

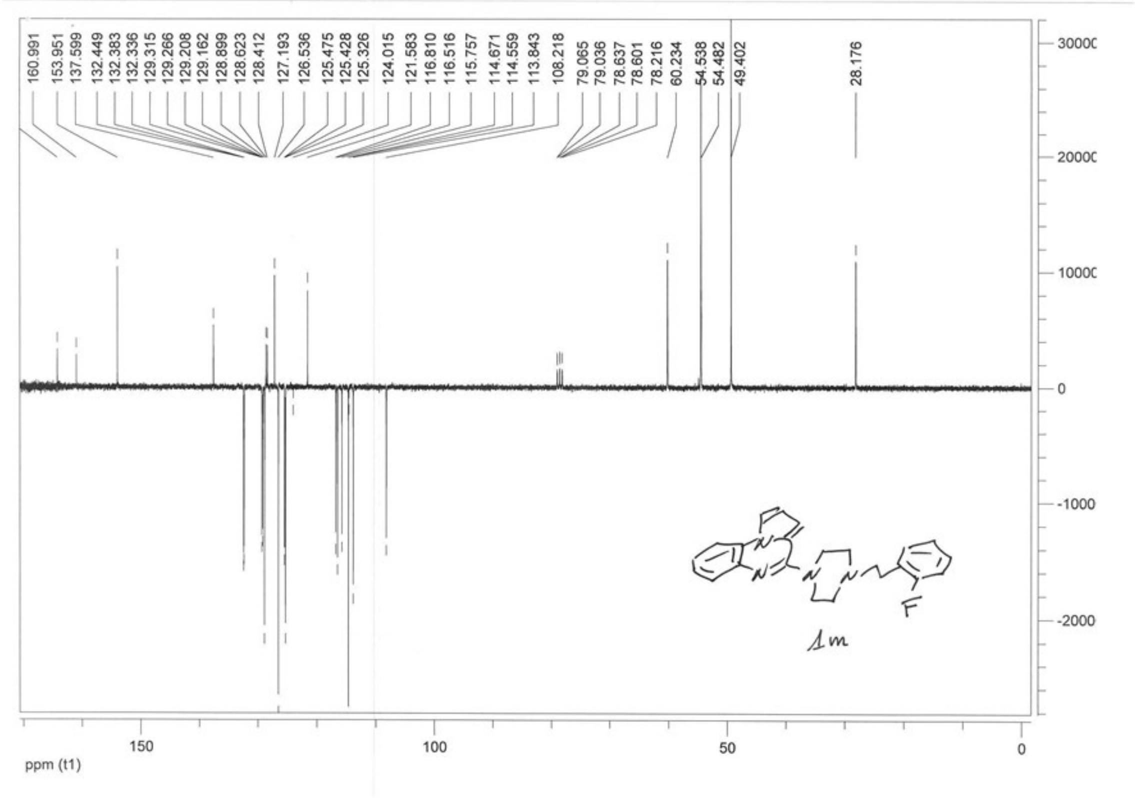

Fig. S25. ^{13}C NMR spectrum of **1m**.

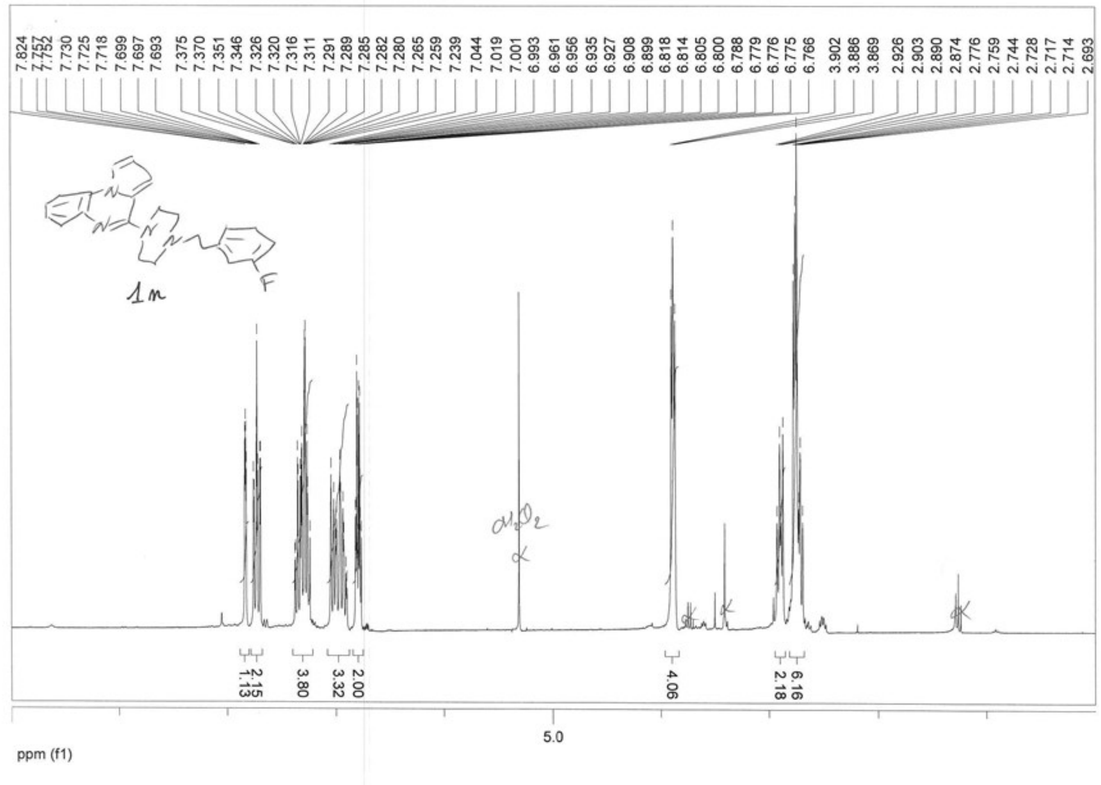

Fig. S26. ^{1}H NMR spectrum of **1n**.

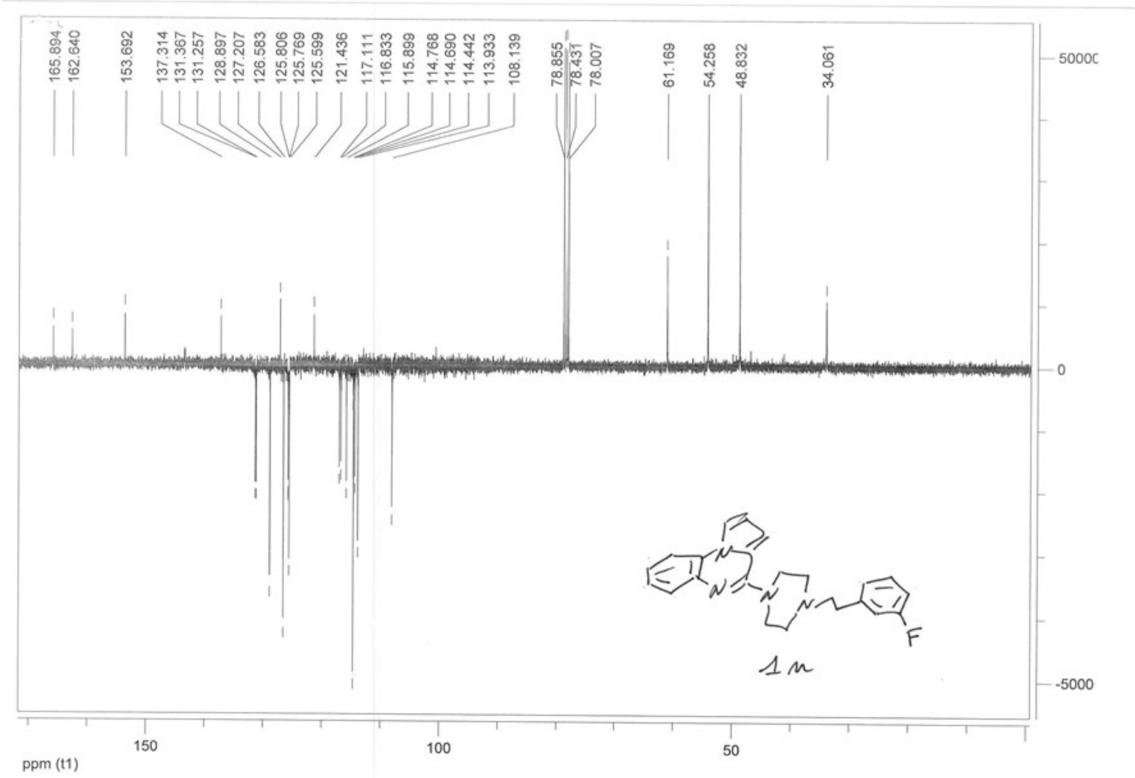

Fig. S27. ^{13}C NMR spectrum of **1n**.

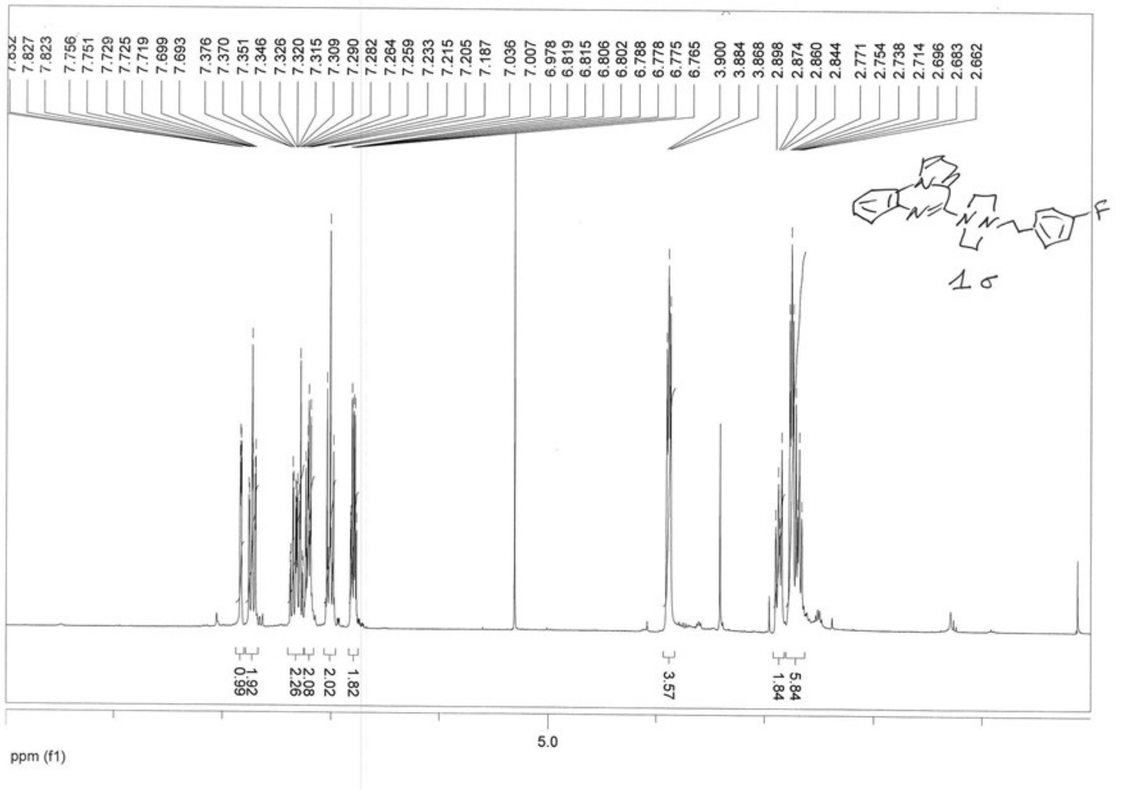

Fig. S28. ^{1}H NMR spectrum of **1o**.

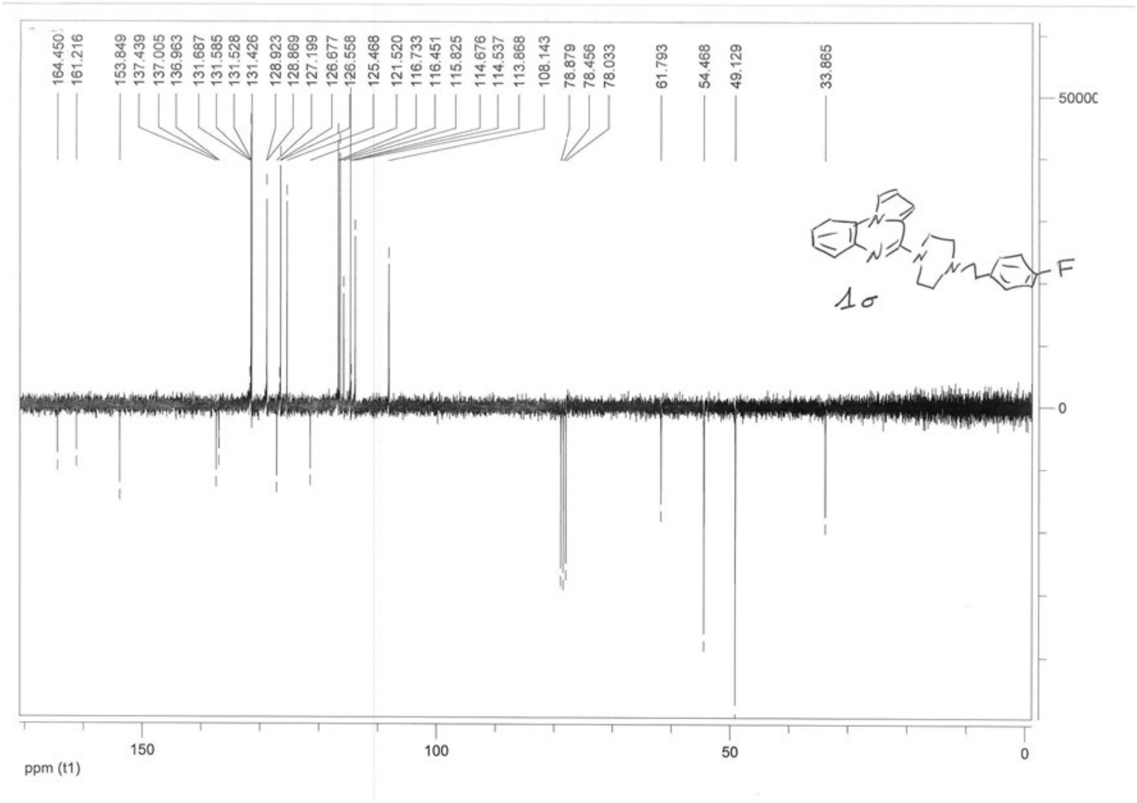

Fig. S29. ^{13}C NMR spectrum of **1o**.

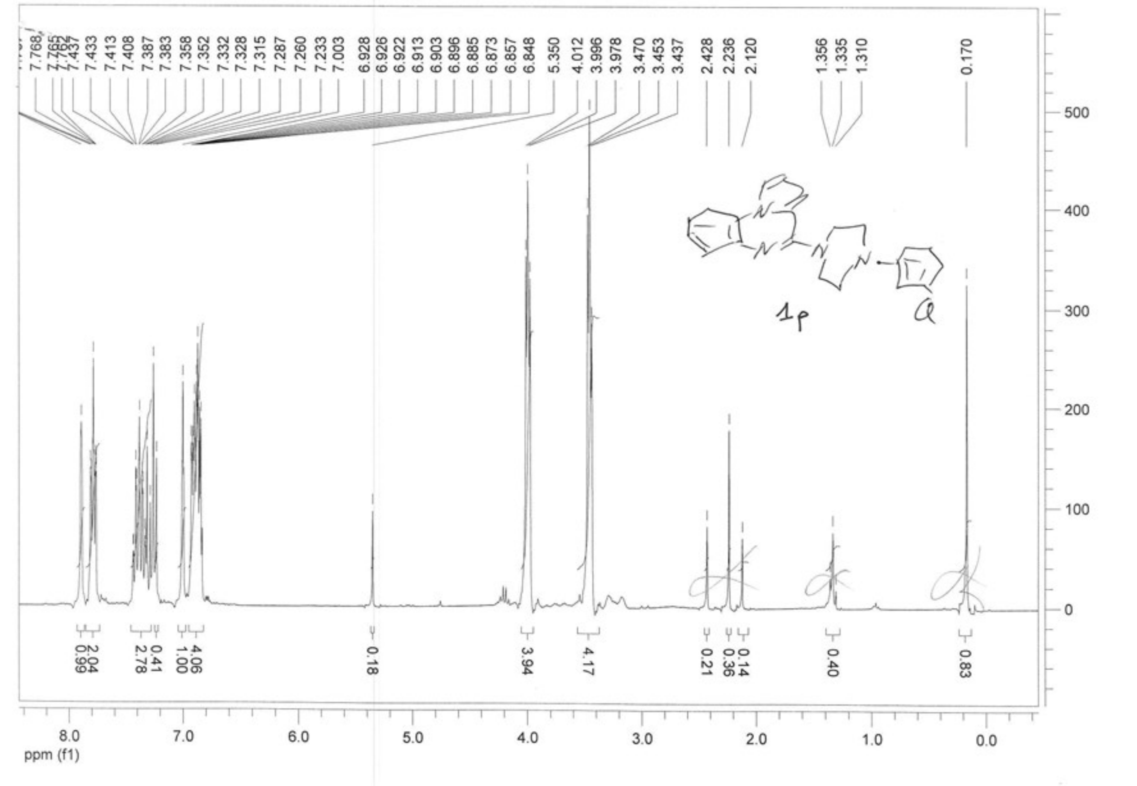

Fig. S30. ¹H NMR spectrum of **1p**.

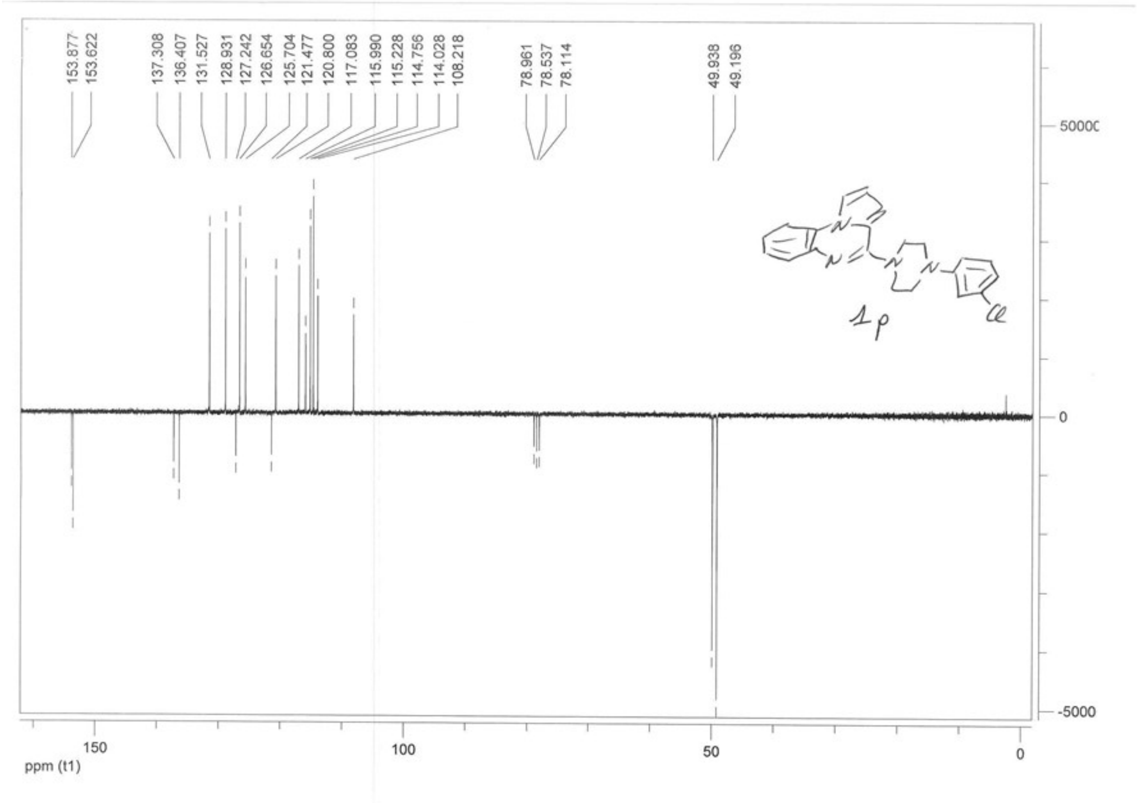

Fig. S31. ^{13}C NMR spectrum of **1p**.

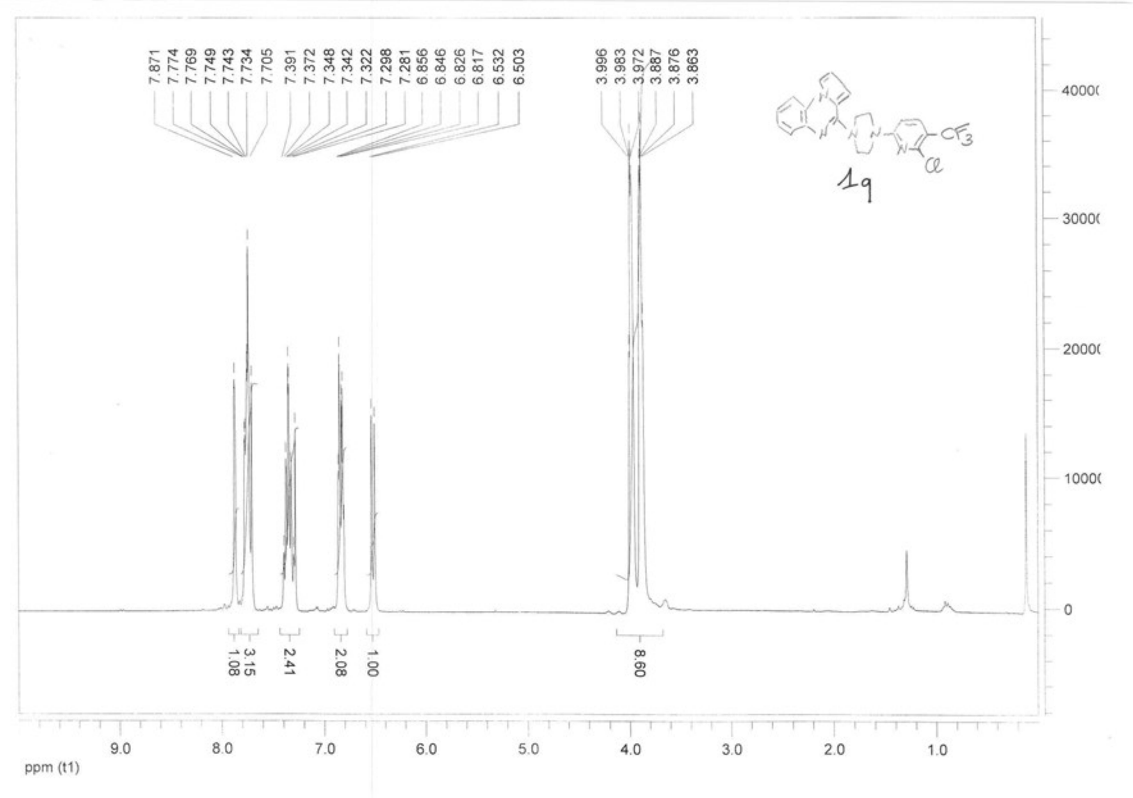

Fig. S32. ^{1}H NMR spectrum of **1q**.

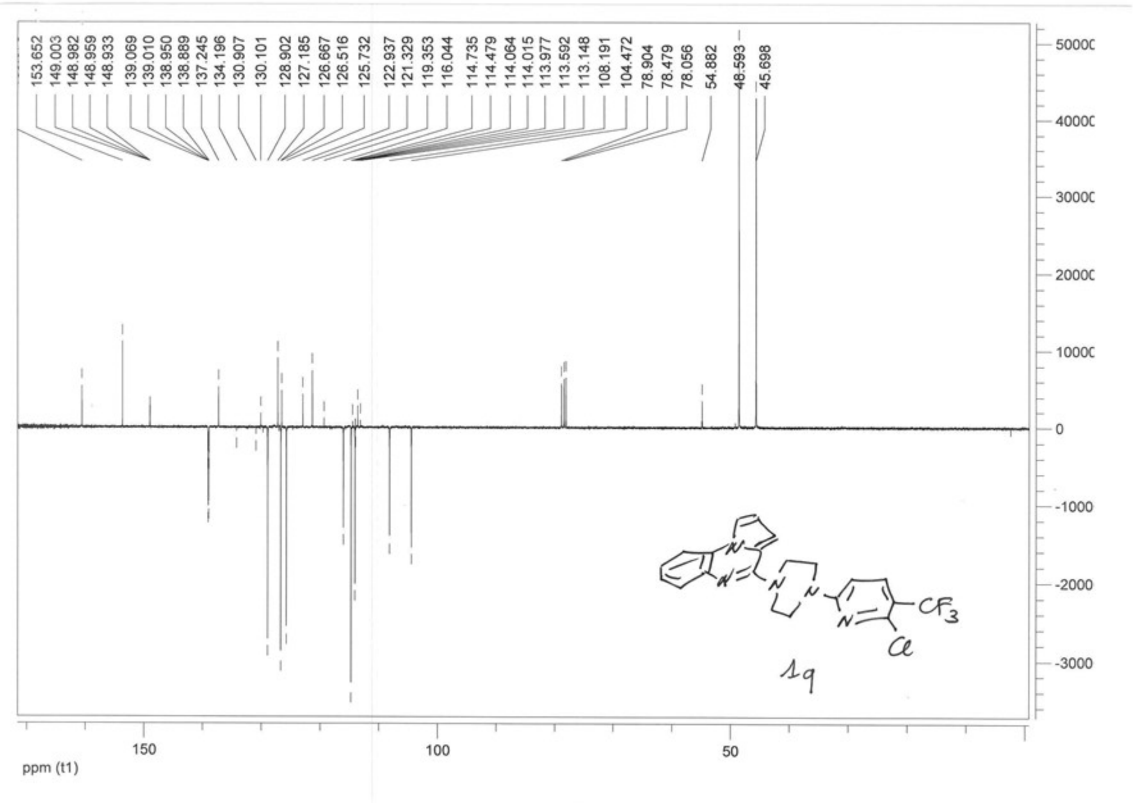

Fig. S33. ^{13}C NMR spectrum of **1q**.

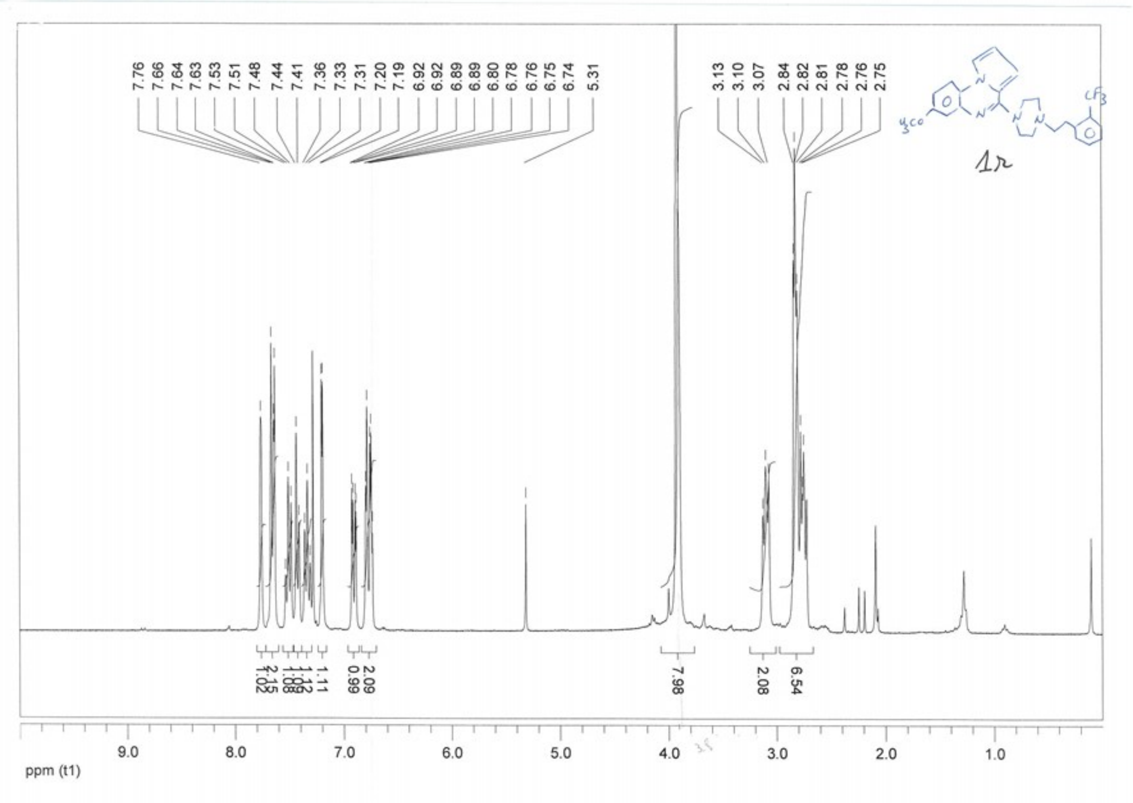

Fig. S34. ^{1}H NMR spectrum of **1r**.

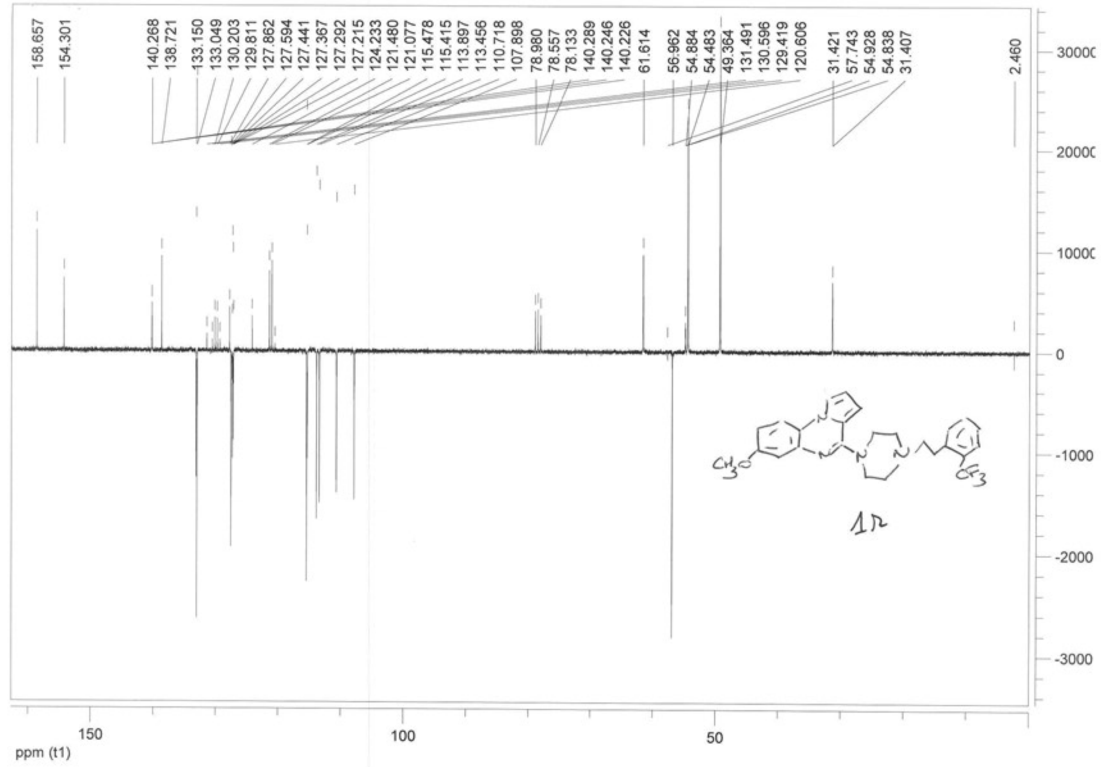

Fig. S35. ^{13}C NMR spectrum of **1r**.

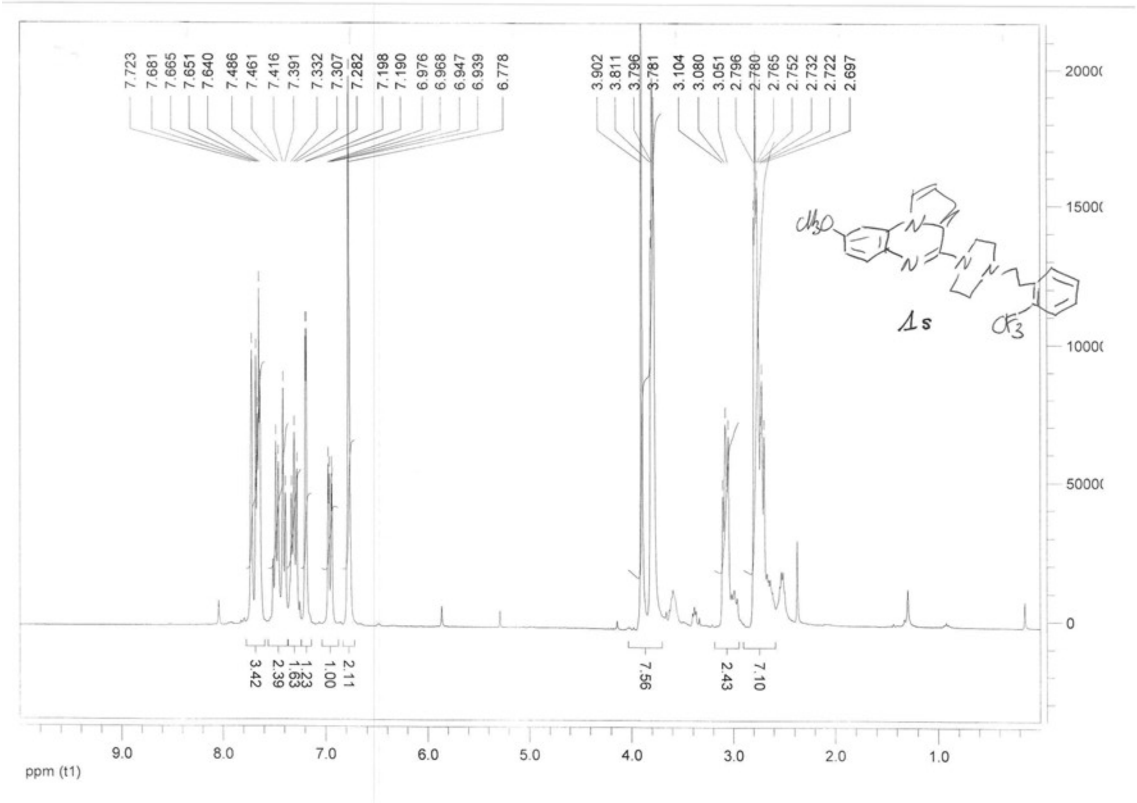

Fig. S36. ^{1}H NMR spectrum of **1s**.

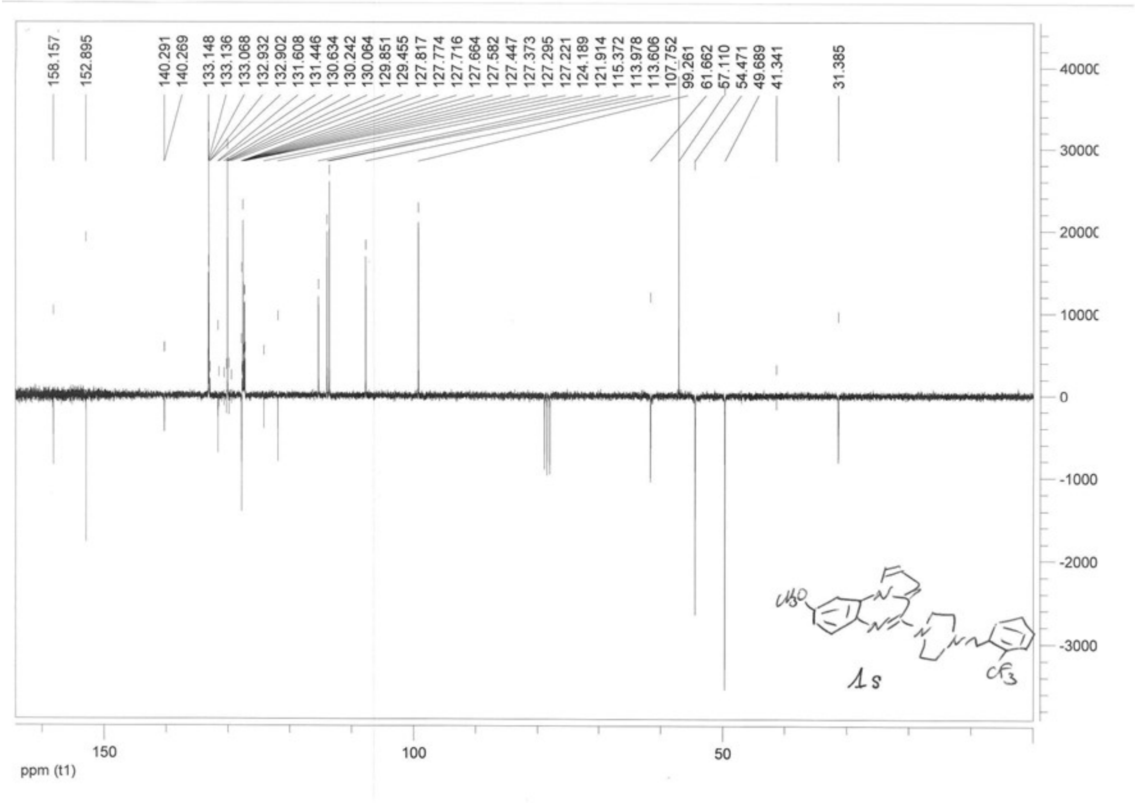

Fig. S37. ^{13}C NMR spectrum of **1s**.

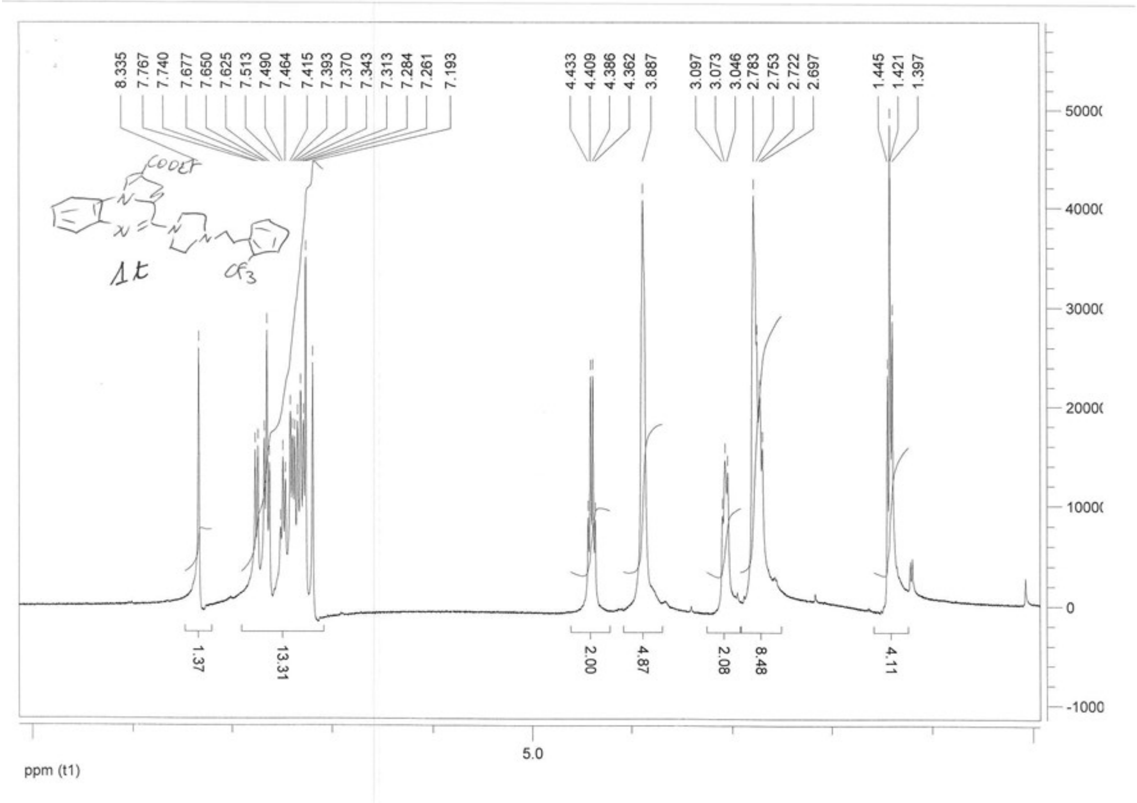

Fig.S38. ^{1}H NMR spectrum of **1t**.

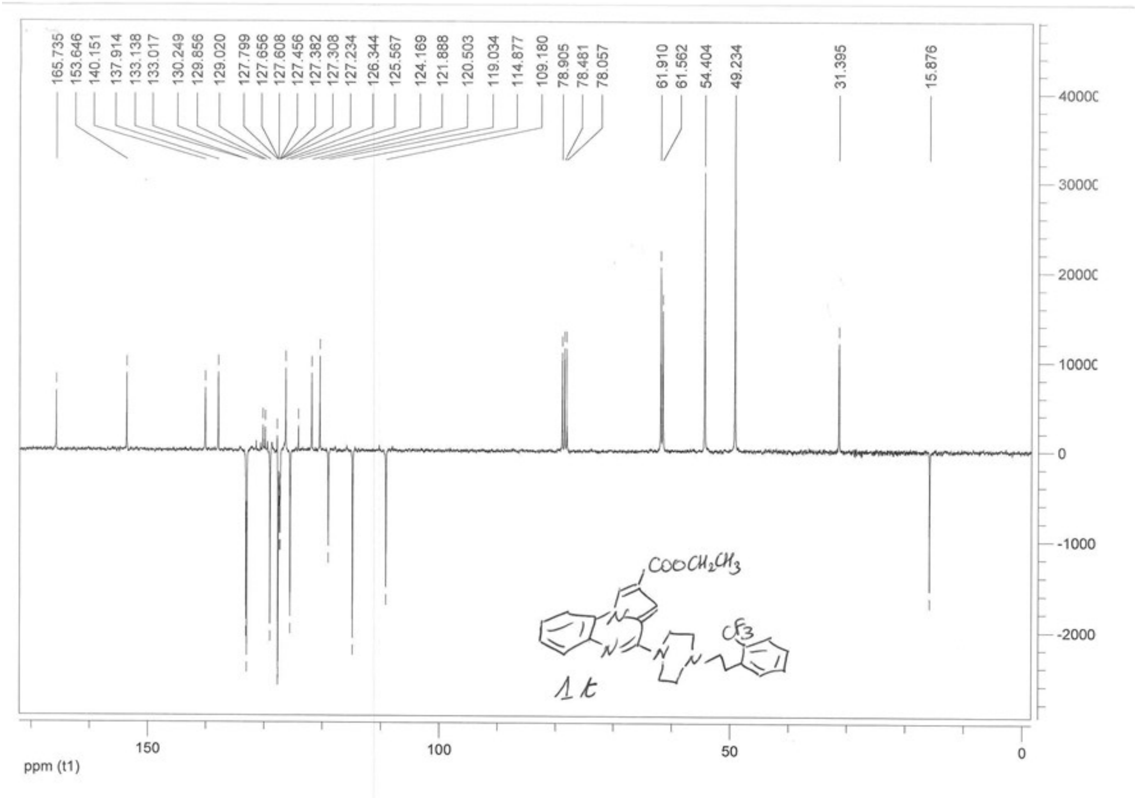

Fig.S39. ^{13}C NMR spectrum of **1t**.

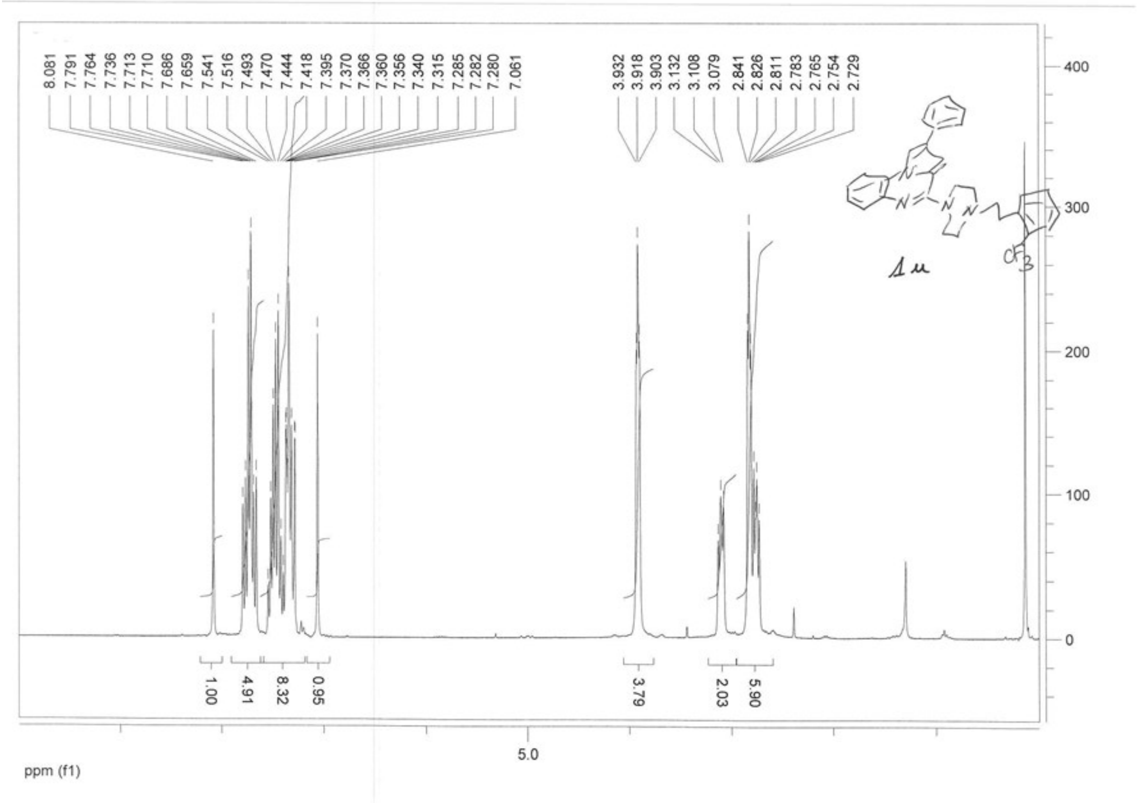

Fig. S40. ^{1}H NMR spectrum of **1u**.

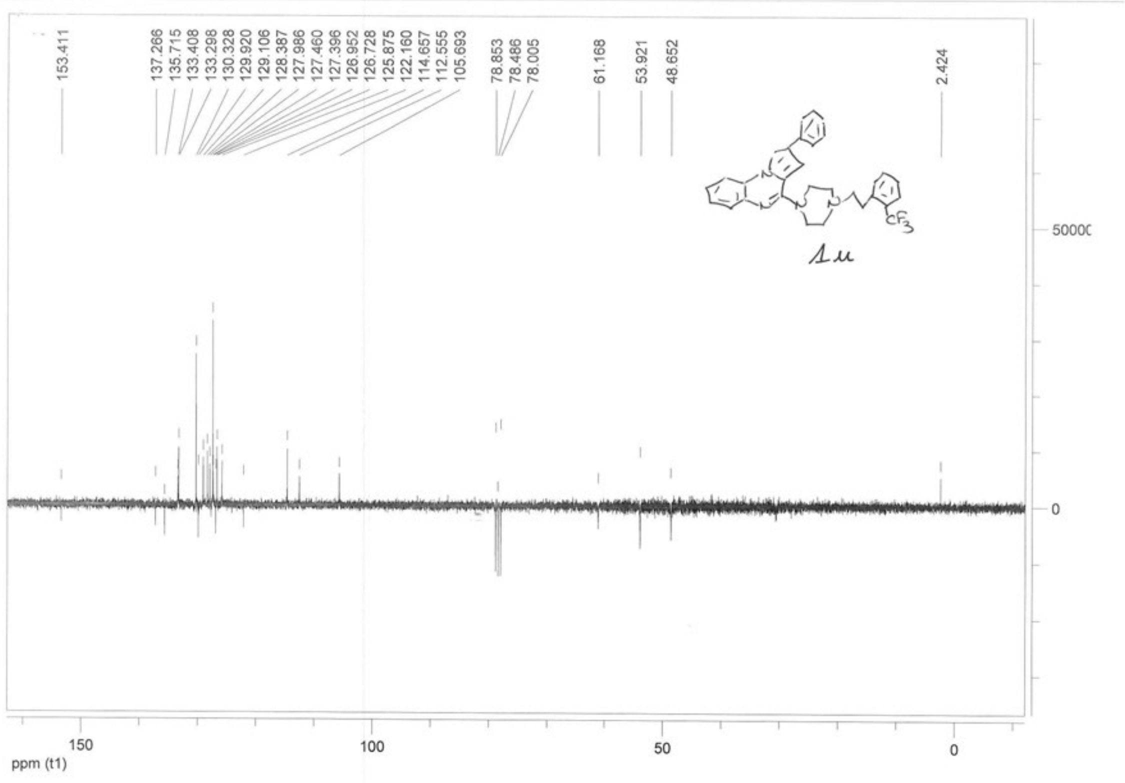

Fig. S41. ^{13}C NMR spectrum of **1u**.

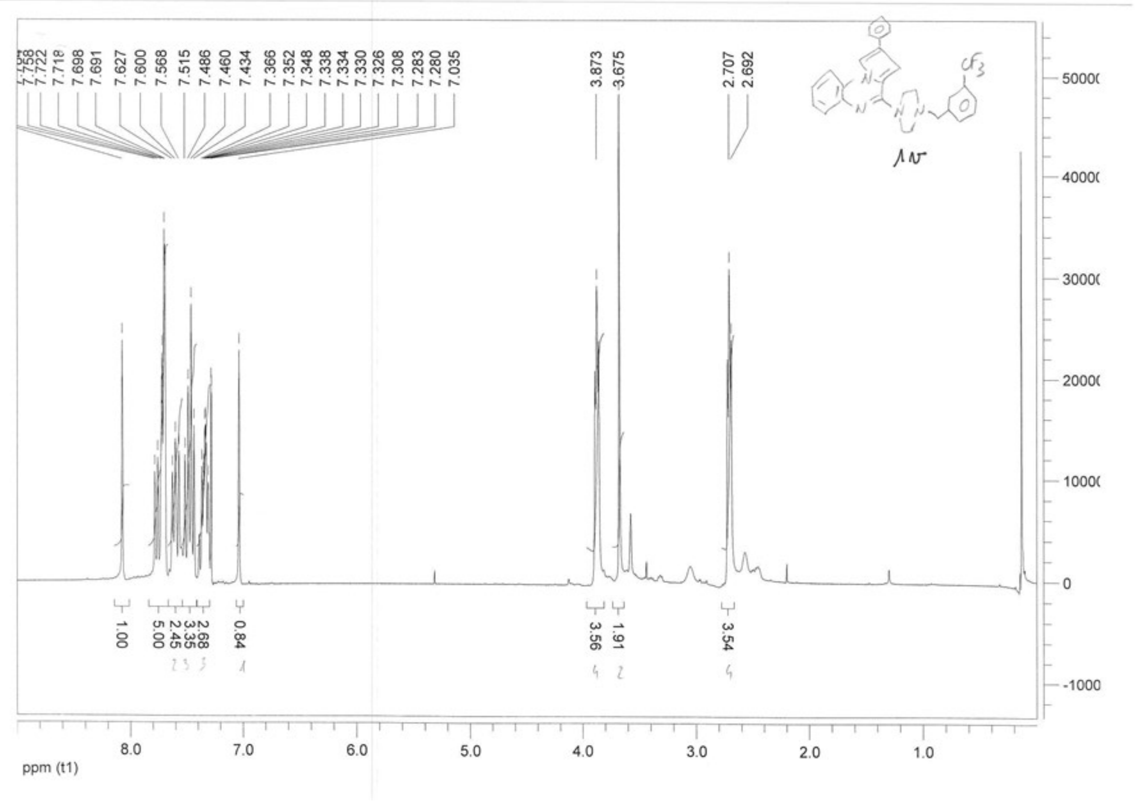

Fig. S42. ^{1}H NMR spectrum of **1v**.

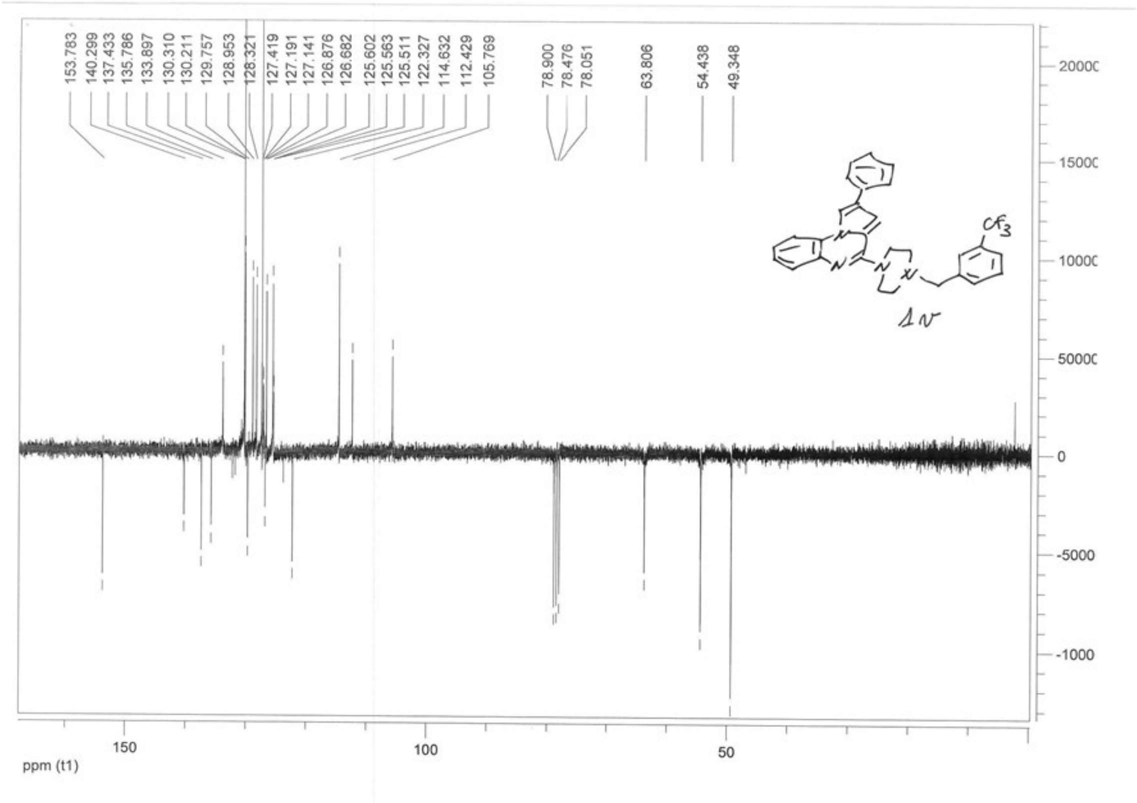

Fig. S43. ^{13}C NMR spectrum of **1v**.

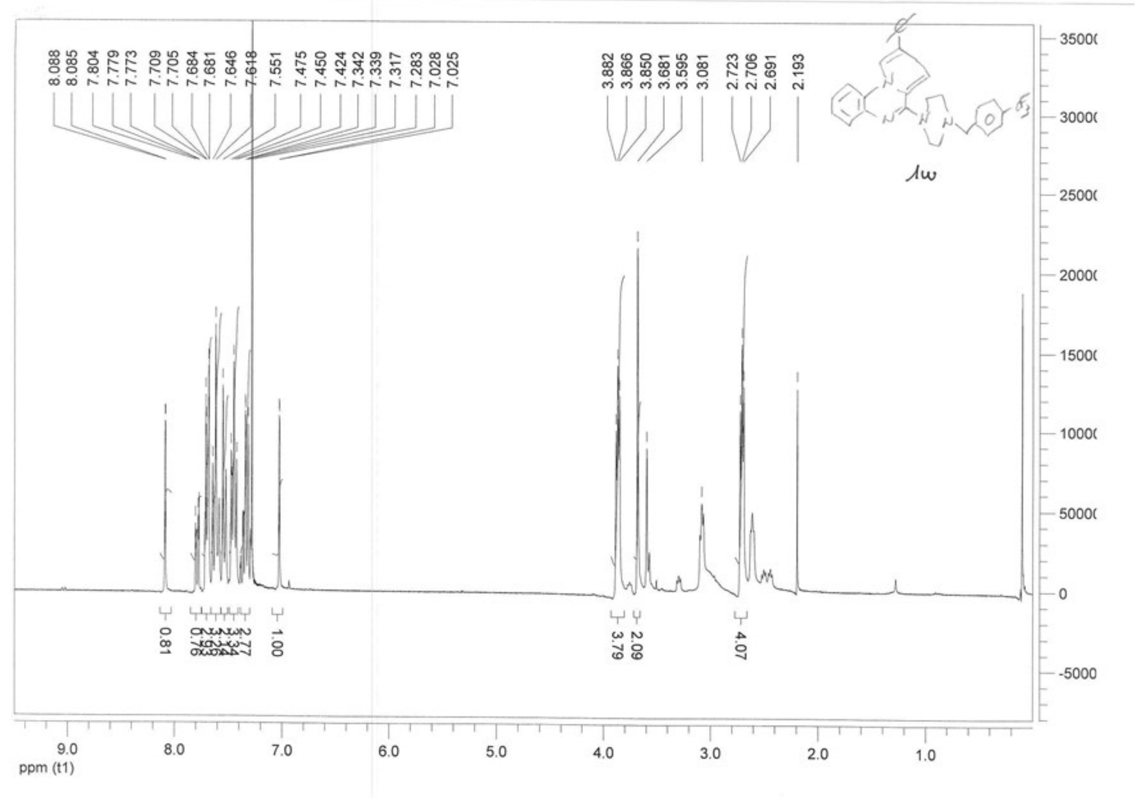

Fig.S44. ^{1}H NMR spectrum of **1w**.

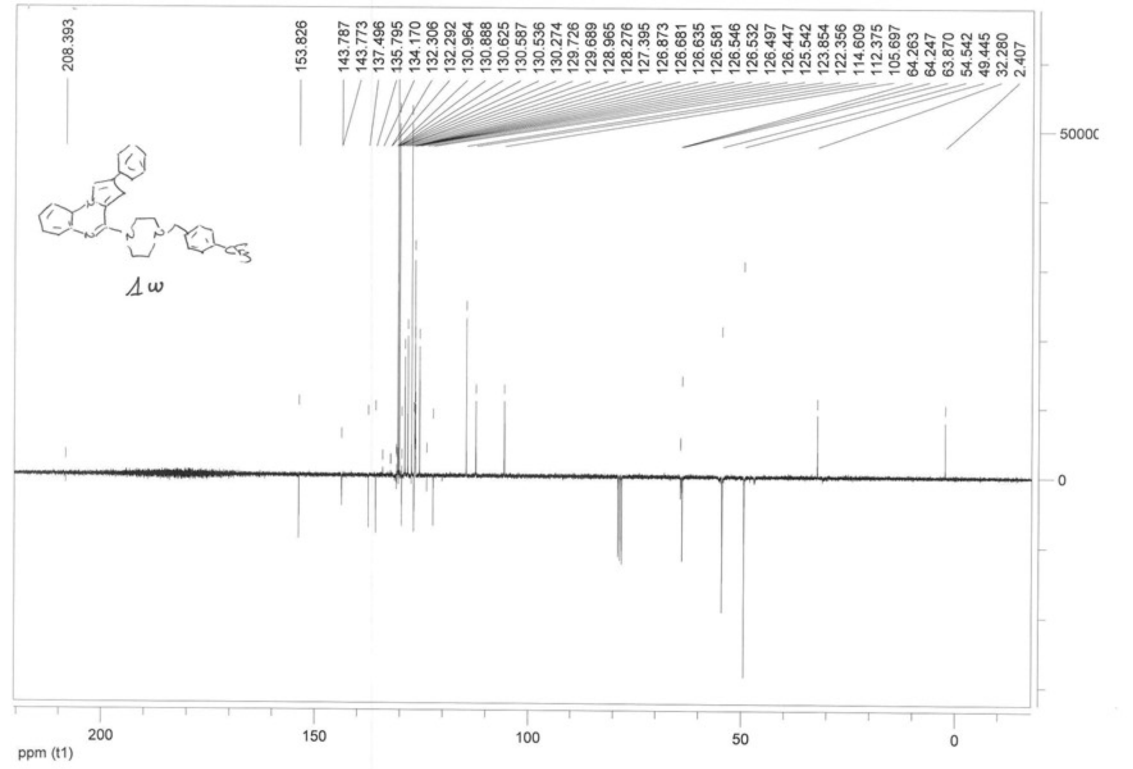

Fig. S45. ^{13}C NMR spectrum of **1w**.

Table S1. Intrinsic cytotoxicity of piperazinyl-pyrrolo[1,2-*a*]quinoxaline derivatives **1a-w** and **2a-f**.

Compounds	Yeast strains	^{a}MIC$_{80}$ (μM)	^{b}RI
	AD1-8u^{-}	810±68	1
**1a**	CDR1	800±86	0.9
	MDR1	789±85	0.97
	AD1-8u^{-}	799±10	1
**1b**	CDR1	817±87	1.07
	MDR1	800±55	1.0

**1c**	AD1-8u¯	99±10	1
	CDR1	817±87	8.25
	MDR1	400±55	4.04
**1d**	AD1-8u¯	781±30	1
	CDR1	791±67	1.01
	MDR1	812±75	1.03
**1e**	AD1-8u¯	12±1.3	1
	CDR1	810±86	67.5
	MDR1	789±85	65.75
**1f**	AD1-8u¯	811±55	1
	CDR1	791±86	0.9
	MDR1	799±75	0.9
**1g**	AD1-8u¯	50±6	1
	CDR1	803±83	16
	MDR1	221±31	4.2
**1h**	AD1-8u¯	782±56	1
	CDR1	811±73	1.03
	MDR1	771±72	0.9
**1i**	AD1-8u¯	823±91	1
	CDR1	801±61	0.9
	MDR1	799±51	0.9
**1j**	AD1-8u¯	3±0.4	1
	CDR1	100±12	33.3
	MDR1	25±3	8.33
**1k**	AD1-8u¯	3±0.2	1
	CDR1	400±49	133.3
	MDR1	12±1	4
**1l**	AD1-8u¯	6±0.5	1
	CDR1	803±102	133.8
	MDR1	12±2	2
**1m**	AD1-8u¯	12±1.6	1
	CDR1	408±52	34
	MDR1	53±6.2	4.41
**1n**	AD1-8u¯	25±3.1	1
	CDR1	400±53	16

	MDR1	50±6.2	2
	AD1-8u^{-}	6±0.71	1
**1o**	CDR1	200±31	33.3
	MDR1	25±3.4	4.1
	AD1-8u^{-}	791±81	1
**1p**	CDR1	799±61	1.01
	MDR1	823±77	1.04
	AD1-8u^{-}	825±65	1
**1q**	CDR1	778±91	0.94
	MDR1	800±78	0.96
	AD1-8u^{-}	6±0.5	1
**1r**	CDR1	776±81	129.3
	MDR1	12±1.7	2
	AD1-8u^{-}	28±1.7	1
**1s**	CDR1	387±47	13.8
	MDR1	59±4.7	2.1
	AD1-8u^{-}	94±7.3	1
**1t**	CDR1	821±75	8.7
	MDR1	102±9.3	1.08
	AD1-8u^{-}	767±81	1
**1u**	CDR1	822±70	1.07
	MDR1	811±92	1.05
	AD1-8u^{-}	816±96	1
**1v**	CDR1	794±71	1.02
	MDR1	801±88	0.98
	AD1-8u^{-}	104±8.4	1
**1w**	CDR1	901±77	8.6
	MDR1	187±16	1.7
	AD1-8u^{-}	47±2.9	1
**2a**	CDR1	412±27	8
	MDR1	91±7.9	1.9
	AD1-8u^{-}	94±110	1
**2b**	CDR1	811±119	8.6
	MDR1	104±121	1.1
**2c**	AD1-8u^{-}	3±0.1	1

	CDR1	817±77	272.3
	MDR1	6±0.2	2
**2d**	AD1-8u⁻	6±0.2	1
	CDR1	831±88	138.5
	MDR1	5±0.2	0.83
**2e**	AD1-8u⁻	3±0.1	1
	CDR1	821±86	273.6
	MDR1	6±0.2	2
**2f**	AD1-8u⁻	6±0.1	1
	CDR1	804±71	134
	MDR1	14±1.2	2.33

[a] The MIC_{80} values of cytotoxicity were determined by measuring the optical density of cultures of each strain in the absence and the presence of a range of concentrations of the different compounds. Yeast growth in the absence of inhibitor was considered as 100%, and the concentration where the growth was decreased to 80% was taken as MIC_{80}. The values are the means ± standard deviations of three independent experiments. [b] The resistance index (RI) was calculated as the ratio between the MIC_{80} values determined for the strain overexpressing the transporter relatively to that of the control strain (AD1-8u⁻).

Table S2. Ability of piperazinyl-pyrrolo[1,2-*a*]quinoxaline derivatives to sensitize yeast growth to FLC cytotoxicity.

Strain	Compound	[a]FIC of fluconazole	[b]FIC of compound	[c]FICI
**AD1-8u⁻**	**1a**	1 (1.5/1.5)	1 (810/810)	2 (1+1)
	**1b**	1 (1.5/1.5)	1 (799/799)	2 (1+1)
	**1c**	1 (1.5/1.5)	1 (99/99)	2 (1+1)
	**1d**	1 (1.5/1.5)	1 (781/781)	2 (1+1)
	**1e**	1 (1.5/1.5)	1 (12/12)	2 (1+1)
	**1f**	1 (1.5/1.5)	1 (811/811)	2 (1+1)
	**1g**	1 (1.5/1.5)	1 (50/50)	2 (1+1)
	**1h**	1 (1.5/1.5)	1 (782/782)	2 (1+1)
	**1i**	1 (1.5/1.5)	1 (823/823)	2 (1+1)
	**1j**	1 (1.5/1.5)	1 (3/3)	2 (1+1)
	**1k**	1 (1.5/1.5)	1 (3/3)	2 (1+1)
	**1l**	1 (1.5/1.5)	1 (6/6)	2 (1+1)
	**1m**	1 (1.5/1.5)	1 (12/12)	2 (1+1)
	**1n**	1 (1.5/1.5)	1 (25/25)	2 (1+1)

	**1o**	1 (1.5/1.5)	1 (6/6)	2 (1+1)
	**1p**	1 (1.5/1.5)	1 (791/791)	2 (1+1)
	**1q**	1 (1.5/1.5)	1 (825/825)	2 (1+1)
	**1r**	1 (1.5/1.5)	1 (6/6)	2 (1+1)
	**1s**	1 (1.5/1.5)	1 (28/28)	2 (1+1)
	**1t**	1 (1.5/1.5)	1 (94/94)	2 (1+1)
	**1u**	1 (1.5/1.5)	1 (767/767)	2 (1+1)
	**1v**	1 (1.5/1.5)	1 (816/816)	2 (1+1)
	**1w**	1 (1.5/1.5)	1 (104/104)	2 (1+1)
	**2a**	1 (1.5/1.5)	1 (47/47)	2 (1+1)
	**2b**	1 (1.5/1.5)	1 (94/94)	2 (1+1)
	**2c**	1 (1.5/1.5)	1 (3/3)	2 (1+1)
	**2d**	1 (1.5/1.5)	1 (6/6)	2 (1+1)
	**2e**	1 (1.5/1.5)	1 (3/3)	2 (1+1)
	**2f**	1 (1.5/1.5)	1 (6/6)	2 (1+1)
**AD1-CDR1**	**1a**	0.7 (163/209)	1 (800/800)	1.7 (0.7+1)
	**1b**	0.7 (163/209)	1 (817/817)	1.7 (0.7+1)
	**1c**	0.3 (81/209)	1 (817/817)	1.3 (0.3+1)
	**1d**	0.3 (81/209)	1 (791/791)	1.3 (0.3+1)
	**1e**	0.7 (167/209)	1 (810/810)	1.7 (0.7+1)
	**1f**	0.1 (40/209)	1 (791/791)	1.1 (0.1+1)
	**1g**	0.3 (81/209)	1 (803/803)	1.3 (0.3+1)
	**1h**	0.7 (163/209)	1 (811/811)	1.7 (0.7+1)
	**1i**	0.7 (163/209)	1 (801/801)	1.7 (0.7+1)
	**1j**	0.09 (20/209)	1 (100/100)	1.09 (0.09+1)
	**1k**	0.7 (163/209)	1 (400/400)	1.7 (0.7+1)
	**1l**	0.3 (81/209)	1 (803/803)	1.3 (0.3+1)
	**1m**	0.1 (40/209)	1 (408/408)	1.1 (0.1+1)
	**1n**	0.7 (163/209)	1 (400/400)	1.7 (0.7+1)
	**1o**	0.7 (163/209)	1 (200/200)	1.7 (0.7+1)
	**1p**	0.3 (81/209)	1 (799/799)	1.3 (0.3+1)
	**1q**	0.7 (167/209)	1 (778/778)	1.7 (0.7+1)
	**1r**	0.3 (81/209)	1 (776/776)	1.3 (0.3+1)
	**1s**	0.7 (163/209)	1 (387/387)	1.7 (0.7+1)
	**1t**	0.3 (81/209)	1 (821/821)	1.3 (0.3+1)
	**1u**	0.1 (40/209)	1 (822/822)	1.1 (0.1+1)
	**1v**	0.7 (163/209)	1 (794/794)	1.7 (0.7+1)
	**1w**	0.1 (40/209)	1 (901/901)	1.1 (0.1+1)

	**2a**	0.3 (81/209)	1 (412/412)	1.3 (0.3+1)
	**2b**	0.7 (163/209)	1 (811/811)	1.7 (0.7+1)
	**2c**	0.7 (163/209)	1 (817/817)	1.7 (0.7+1)
	**2d**	0.09 (20/209)	1 (831/841)	1.09 (0.09+1)
	**2e**	0.7 (163/209)	1 (821/821)	1.7 (0.7+1)
	**2f**	0.1 (40/209)	1 (804/804)	1.1 (0.1+1)
**AD1-MDR1**	**1a**	0.6 (40/65)	1 (789/789)	1.6 (0.6+1)
	**1b**	0.3 (20/65)	1 (800/800)	1.3 (0.3+1)
	**1c**	0.3 (20/65)	1 (400/400)	1.3 (0.3+1)
	**1d**	0.15 (10/65)	0.0076 (6.25/812)	0.15
	**1e**	0.6 (40/65)	1 (789/789)	1.6 (0.6+1)
	**1f**	0.15 (10/65)	0.25 (200/799)	0.4 (0.15+0.25)
	**1g**	0.6 (40/65)	1 (221/221)	1.6 (0.6+1)
	**1h**	0.6 (40/65)	1 (771/771)	1.6 (0.6 +1)
	**1i**	0.6 (40/65)	1 (799/799)	1.6 (0.6+1)
	**1j**	0.6 (40/65)	1 (25/25)	1.6 (0.6+1)
	**1k**	0.6 (40/65)	1 (12/12)	1.6 (0.6+1)
	**1l**	0.6 (40/65)	1 (12/12)	1.6 (0.6+1)
	**1m**	0.6 (40/65)	1 (53/53)	1.6 (0.6 +1)
	**1n**	0.6 (40/65)	1 (50/50)	1.6 (0.6+1)
	**1o**	0.3 (20/65)	1 (25/25)	1.3 (0.3+1)
	**1p**	0.3 (20/65)	1 (823/823)	1.3 (0.3+1)
	**1q**	0.6 (40/65)	1 (800/800)	1.6 (0.6+1)
	**1r**	0.6 (40/65)	1 (12/12)	1.6 (0.6+1)
	**1s**	0.3 (20/65)	1 (59/59)	1.3 (0.3+1)
	**1t**	0.3 (20/65)	1 (102/102)	1.3 (0.3+1)
	**1u**	0.3 (20/65)	1 (811/811)	1.3 (0.3+1)
	**1v**	0.6 (40/65)	1 (801/801)	1.6 (0.6 +1)
	**1w**	0.6 (40/65)	1 (187/187)	1.6 (0.6+1)
	**2a**	0.3 (20/65)	1 (91/91)	1.3 (0.3+1)
	**2b**	0.3 (20/65)	1 (104/104)	1.3 (0.3+1)
	**2c**	0.6 (40/65)	1 (6/6)	1.6 (0.6+1)
	**2d**	0.6 (40/65)	1 (5/5)	1.6 (0.6+1)
	**2e**	0.3 (20/65)	1 (6/6)	1.3 (0.3+1)
	**2f**	0.3 (20/65)	1 (14/14)	1.3 (0.3+1)
**F2**	**1d**	1 (13/13)	1 (618/618)	2 (1+1)
	**1f**	0.5 (7.5/13)	1 (400/400)	1.5 (0.5+1)
**F5**	**1d**	0.4 (200/418)	0.2 (150/720)	0.6 (0.2+0.4)
	**1f**	0.35 (150/418)	0.43 (350/799)	0.78 (0.35+0.43)

[a] Evaluated by the checkerboard method, and expressed as the fractional inhibitory concentration (FIC) values for the fluconazole (= MIC_{80} of fluconazole in combination/MIC_{80} of fluconazole alone) and [b] each compound (= MIC_{80} of compound in combination/MIC_{80} of compound alone). The values in brackets are expressed in μM. [c] FIC index (FICI) value ≤ 0.5 indicates synergistic interaction between the compound and the fluconazole.
